# XXVI^e^ Actualités du Pharo. La santé sexuelle et reproductive dans les pays du Sud. 6-8 octobre 2021, Marseille, France

**DOI:** 10.48327/mtsi.2021.180

**Published:** 2021-11-30

**Authors:** Comité d'organisation: GISPE (Groupe d'intervention en santé publique et épidémiologie)

**Keywords:** Santé sexuelle et reproductive, Droits, Médecine tropicale, Pays en développement Keywords: Sexual and reproductive health, Rights, Tropical medicine, Developing countries

## Éditorial

### La santé sexuelle et reproductive dans les pays du Sud. De l’émergence du concept à celle du droit

Jean-Paul BOUTIN

GISPE, 82 boulevard Tellène, 13008 Marseille, France, www.gispe.orgboutin.jeanpaul@gmail.com

Fort du succès de l’édition de 2019 consacrée au concept « One Health, vers une seule santé », le Comité scientifique des Actualités du Pharo a décidé de continuer d'explorer l'actualité des grands thèmes transversaux de développement de la santé mondiale sous l'angle de leur réalité en zone tropicale. C'est ainsi qu'en 2020 fut choisi comme thème principal pour la XXVI^e^ édition des Actualités du Pharo les progrès en matière de droits à la « Santé sexuelle et reproductive » 26 ans après que la Conférence du Caire de septembre 1994 en ait édicté les principes.

Mais nous n’étions sûrement pas prêts pour affronter un tel sujet, et c'est probablement pourquoi un petit coronavirus émergent nous a contraints à remettre notre ouvrage sur le métier et à attendre ce mois d'octobre 2021 pour vous proposer un programme plus complet et encore plus pertinent sur un si vaste sujet.

Comme le définissait la Conférence du Caire, avoir droit à une bonne santé sexuelle et reproductive « suppose qu'une personne [puisse] mener une vie sexuelle satisfaisante en toute sécurité, qu'elle [soit} capable de procréer et libre de le faire aussi souvent ou aussi peu souvent qu'elle le désire… [qu'elle ait] le droit d’être informée et d'utiliser la méthode de planification familiale de son choix, … [qu'elle ait] le choix des méthodes de régulation des naissances qui ne soient pas contraires à la loi, … sûres, efficaces, abordables et acceptables, … [qu'elle ait] le droit d'accéder à des services de santé qui permettent aux femmes de mener à bien grossesse et accouchement et donnent aux couples toutes les chances d'avoir un enfant en bonne santé. … On entend également par cette expression la santé en matière de sexualité, qui vise à améliorer la qualité de la vie et des relations interpersonnelles, et non à se borner à dispenser conseils et soins relatifs à la procréation et aux maladies sexuellement transmissibles. »

Pour le GISPE, organisateur des Actualités du Pharo, il paraît essentiel d'encourager le développement du droit à la Santé sexuelle et reproductive dans les pays francophones du Sud, en proposant à Marseille un lieu d’échange d'expériences, en favorisant des rencontres ou dorénavant des contacts à distance, en restant un agitateur d'idées. Bon congrès et bonne lecture!

**Figure 1:**
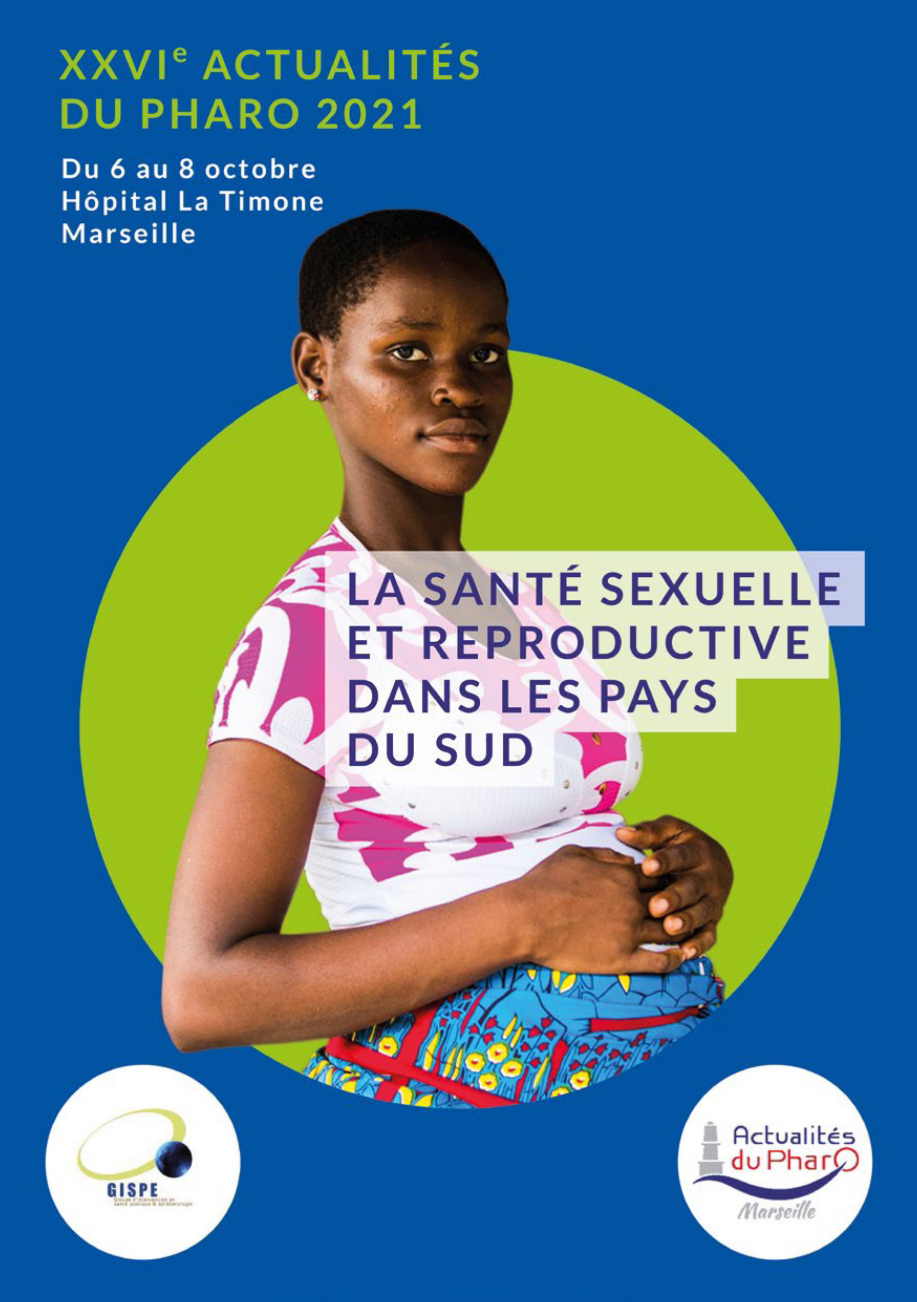
Affiche des XXVI^e^ Actualités du Pharo. La santé sexuelle et reproductive dans les pays du Sud Poster of the XXVI^th^ News of the Pharo. Sexual and reproductive health in the South

