# Supplementary material for: XXVIth News of the Pharo. Sexual and reproductive health in the South. 6-8 October 2021, Marseille, France
Source: Med Trop Sante Int. 2021 Nov 30;1(4):mtsi.2021.180. [Article in French] doi: 10.48327/mtsi.2021.180 (PMC9128466; doi:10.48327/mtsi.2021.180)
Supplement: Related texts

# Research article: Droits à la santé sexuelle et reproductive: généalogie d'un concept et défis actuels

**Published:** 2021-11-30

**Authors:** Aurélie Gal-Regniez, Fatou Sow

**Affiliations:** Equipop Bureau de Paris, 6 rue de la Plaine 75020 Paris, France

**Keywords:** DSSR, Sexualité, Droits humains, Santé, Procréation

Les droits et la santé sexuels et reproductifs (DSSR) sont « les droits de toute personne de prendre des décisions libres, informées et responsables et d'exercer un contrôle entier sur les aspects élémentaires de sa vie privée - corps, sexualité, santé, relations avec autrui, liberté de se marier ou non, d'avoir ou non des enfants, et le cas échéant, choix du moment et du partenaire - sans s'exposer à quelle que forme de discrimination, de stigmatisation, de contrainte ou de violence que ce soit. Ceci recouvre le droit de jouir de sa propre sexualité et de l'exprimer, de pouvoir - sans ingérence extérieure - prendre des décisions personnelles concernant sa sexualité et la procréation, ainsi que d'avoir accès à l'information, à l’éducation et aux services en matière de santé sexuelle et procréative »^1^. Le concept de DSSR, largement mobilisé aujourd'hui, s'est formalisé à partir du dernier quart du XX^e^ siècle sous l'influence d'une pluralité d'actrices et d'acteurs animés par des préoccupations féministes, sanitaires ou politiques. En rupture avec les approches de contrôle démographique et dépassant le cadre exclusif de la santé maternelle, il entérine finalement la possibilité de dissociation entre sexualité et fécondité. D'un point de vue de santé publique, les DSSR envisagent l'individu dans son cycle de vie, en considérant un ensemble de problématiques (de la maternité aux cancers gynécologiques en passant par la prévention du VIH ou encore le traitement de l'infécondité) sous l'angle aussi bien de l'accès aux soins que de l'accès à l'information, l’éducation à la sexualité en étant un élément important. Mais les DSSR mettent surtout l'accent sur les droits humains tout en rendant visibles les liens entre santé et inégalités de genre dans nos sociétés patriarcales. Réifiés au rang de norme internationale, grâce notamment au Programme d'Action du Caire de 1994 puis à celui de Pékin en 1995, les DSSR ont dès lors été appropriés ou détournés, défendus ou combattus. Plus de 25 ans après l’établissement de ces fiches de route internationales, où en sommes-nous de leur mise en œuvre? Quelle place pour les revendications des femmes et pour les pensées féministes dans le champ de la santé? Pourquoi est-il toujours légitime de se poser la question: « À qui appartient le corps des femmes »? Quels sont les contours de ce questionnement dans des régions comme l'Afrique de l'Ouest francophone? En quoi est-il crucial d'intégrer plus fortement les énonciations des mouvements féministes sur ces questions pour renouveler les approches de promotion des DSSR?

# Research article: La santé des femmes en situation de conflit: du paternalisme vers une maïeutique humanitaire. Changement de paradigme?

**Published:** 2021-11-30

**Authors:** Nelly Staderini

**Affiliations:** Médecins Sans Frontières (MSF), Rue de Lausanne 78, 1202 Genève, Suisse

**Keywords:** Santé, Femmes, Innovation, Autonomisation, Autogestion, Conflit

Médecins Sans Frontières a 50 ans. Cinquante ans d'action dans l'urgence et auprès de populations dites vulnérables. La santé des femmes y occupe une grande partie avec notamment plus de 300 000 accouchements assistés annuellement.

Au-delà du suivi de grossesses et de la période du post-partum, MSF met un accent particulier sur l'offre de contraception, et la gestion des avortements et de leurs complications. Un investissement plus récent s'est fait sur la prévention du cancer du col de l'utérus et sur les maladies sexuellement transmissibles au-delà du VIH.

En dehors d'un élargissement de son offre de soin, MSF s'est adapté aux contraintes d'accès aux populations en développant de nouvelles approches centrées sur les patients.

Les difficultés d'accès aux soins, la simplification de certains outils diagnostiques et autres protocoles de prise en charge permettent actuellement de nouveaux modèles de soins, les rendant potentiellement plus accessibles comme l'autogestion.

Elle peut désigner la prise d'une pilule contraceptive, la palpation de la poitrine à la recherche de grosseurs, le diagnostic d'une grossesse en cours ou la prise en charge d'un avortement. C'est aussi effectuer son propre dépistage du cancer du col de l'utérus via l'auto-prélèvement pour le test du papillomavirus humain ou autotest salivaire à domicile pour le VIH.

Avec le soutien nécessaire de personnes expérimentées, l'auto-prise en charge permet aux populations de reprendre le contrôle de leur santé. Et surtout, elle repose sur des méthodes fiables et favorables au bien-être des populations.

Pour les femmes et jeunes filles qui ne peuvent accéder facilement aux soins, l'auto-prise en charge, pratiquée avec ou sans l'assistance d'un professionnel de santé, peut les aider à rester en bonne santé et savoir comment s'informer sur les questions de santé auprès d'une personne de confiance ou sur une application numérique.

Ces nouvelles perspectives transforment les relations soignants-patients et vont vers plus d'autonomie dans les soins tout en garantissant une qualité similaire. Une perspective bienvenue pour les femmes.

Pour effectuer ce changement de paradigme, la formation du personnel soignant, ainsi que le transfert de savoir et de compétences aux femmes est nécessaire. Faire confiance aux populations et mettre à disposition une offre de soin plus proche de la communauté est le défi à relever aujourd'hui pour répondre aux enjeux humanitaires de demain.

# Research article: Stratégie mondiale d’élimination du cancer du col de l'utérus: opportunités et défis pour la vaccination HPV

**Published:** 2021-11-30

**Authors:** Paul Bloem

**Affiliations:** Organisation mondiale de la Santé (OMS), Avenue Appia 20, 1202 Genève, Suisse

**Keywords:** Stratégie, Élimination, Vaccination, HPV, Papillomavirus

Le cancer du col de l'utérus est une maladie évitable. Il est également guérissable s'il est détecté tôt et traité de manière adéquate. Pourtant, il reste l'un des cancers et l'une des causes de décès liés au cancer les plus fréquents chez les femmes du monde entier.

Le nombre annuel de nouveaux cas de cancer du col de l'utérus devrait passer de 570 000 à 700 000 entre 2018 et 2030, et le nombre annuel de décès devrait passer de 311 000 à 400 000. Plus de 85% des personnes touchées sont des femmes jeunes et sous-éduquées qui vivent dans les pays les plus pauvres du monde. Beaucoup sont également mères de jeunes enfants dont la survie est ensuite tronquée par le décès prématuré de leur mère.

Peu de maladies reflètent autant les inégalités mondiales que le cancer du col de l'utérus. Dans les pays à revenu faible et intermédiaire, son incidence est presque deux fois plus élevée et ses taux de mortalité trois fois plus élevés que dans les pays à revenu élevé. Il existe des mesures éprouvées et rentables pour éliminer le cancer du col de l'utérus, mais à ce jour, elles n'ont pas été largement mises en œuvre dans les régions du monde où la charge de la maladie est la plus élevée. Pour une efficacité optimale, ces mesures doivent être adaptées au niveau national et mises en œuvre au moyen de plates-formes de services de santé sensibles aux besoins des femmes, à leur situation sociale et aux besoins des femmes, à leur situation sociale et aux obstacles personnels, culturels, sociaux, structurels et économiques qui entravent leur accès aux services de santé.

Il est essentiel que les services de santé soient intégrés et centrés sur les personnes, et qu'ils respectent et défendent les droits et la dignité des femmes. Une action urgente et audacieuse est nécessaire pour intensifier et pérenniser la mise en œuvre des interventions fondées sur des données probantes (vaccination contre le papillomavirus humain (HPV), dépistage du cancer du col de l'utérus et prise en charge de la maladie détectée) afin d’éliminer le cancer du col de l'utérus en tant que problème de santé publique, mais cette action doit être stratégique.

Cette stratégie mondiale d’élimination du cancer du col de l'utérus propose:

- une vision d'un monde où le cancer du col de l'utérus est éliminé en tant que problème de santé publique;
- un seuil de 4 pour 100 000 années-femmes pour l’élimination en tant que problème de santé publique;
- les objectifs 90-70-90 suivants, qui doivent être atteints d'ici 2030 pour que les pays soient sur la voie de l’élimination du cancer du col de l'utérus

Les tendances en matière d'introduction du vaccin contre le VPH dans le monde au cours des dernières années et les perspectives pour la prochaine décennie, ainsi que les progrès et les défis liés à l'obtention d'une couverture élevée seront au cœur de cette présentation.

# Research article: Les mutilations génitales féminines: prévenir, repérer, orienter

**Published:** 2021-11-30

**Authors:** Claire Tantet

**Affiliations:** Hôpital Avicenne, 125, rue de Stalingrad, 93000 Bobigny, France

**Keywords:** Mutilations génitales féminines, Violences sexuelles, Accompagnement holistique, Prévention en médecine des voyages, France

Aujourd'hui en France, les mutilations génitales féminines (MGF) telles que l'excision touchent à la fois des filles et des femmes migrantes ou nées en France de parents originaires de pays où elles sont pratiquées. Pour la majorité, les mutilations génitales féminines sont réalisées lors d'un retour au pays pour les vacances. Or, beaucoup de familles se rendent dans les centres de vaccinations internationales avant un voyage, que ce soit pour la prévention liée aux risques sanitaires, le paludisme ou la mise à jour vaccinale.

L'objectif de cette présentation est de transmettre des connaissances théoriques sur les MGF et aussi d'aborder brièvement des outils pratiques de consultation.

# Research article: Prise en charge chirurgicale des mutilations génitales féminines/excision

**Published:** 2021-11-30

**Authors:** Sébastien Madzou

**Affiliations:** CHU Angers, 4 rue Larrey - 49 933 Angers Cedex 9, France

**Keywords:** Excision, Mutilations génitales féminines, Reconstruction, Chirurgie

Les mutilations génitales féminines sont considérées comme une violation des droits des jeunes filles et des femmes (OMS).

La prise en charge chirurgicale de ces mutilations permet, (i) une réhabilitation de la femme sur le plan juridique, anatomique, sensoriel, sexuel et psychologique; (ii) une réduction de la douleur; (iii) une prévention des complications obstétricales et une participation à la lutte contre la pratique de la mutilation.

Il est important que les professionnels de santé soient formés à la prise en charge des séquelles de l'excision et qu'il y ait une accessibilité à cette prise en charge pour les femmes qui en sont victimes.

# Research article: Fécondité et planification familiale en Afrique: tendances récentes et facteurs associés

**Published:** 2021-11-30

**Authors:** Jean-François Kobiane

**Affiliations:** Institut Supérieur des Sciences de la Population (ISSP), Université Ouagadougou, 03 BP 7118, Blvd Charles De Gaulle, Ouagadougou, Burkina Faso

**Keywords:** Fécondité, Contraception, Âge au mariage, Urbanisation, Éducation, Afrique subsaharienne

Le taux de croissance démographique du continent africain est passé d'un pic de 2,82% en 1980-85, à 2,44% en 2000-2005, puis 2,51% pour la période 2015-2020, selon les statistiques de la Division de la population des Nations unies. Malgré une tendance à la baisse, la croissance démographique sur le continent sera toujours la plus élevée au monde en 2050 (1,74% contre 0,53% au niveau mondial). Cette forte croissance démographique masque des disparités régionales, avec des taux de croissance démographique plus élevés en Afrique centrale, occidentale et orientale, et une croissance démographique plus faible en Afrique du Nord et australe. Comme la tendance générale, les variations régionales sont le reflet des tendances de la mortalité et de la fécondité. Conséquence des progrès des mesures de santé publique et de la technologie médicale, l'espérance de vie a fortement progressé sur la période 1990-1995/2015-2020, passant de 51,9 ans à 62,7 ans. Contrairement à la mortalité, la fécondité en Afrique a baissé à un rythme plus lent: sur une période de 25 ans, l'indice synthétique de fécondité (ISF) est passé de 5,72 enfants en moyenne par femme (contre une moyenne mondiale de 3,01) en 1990-1995 à 4,44 enfants en moyenne par femme (2,47 au niveau mondial) en 2015-2020. Les niveaux de fécondité restent élevés en Afrique centrale et occidentale, tandis qu'en Afrique de l'Est, la baisse de la fécondité au cours des deux dernières décennies est comparable à la moyenne continentale. En revanche, l'Afrique australe et l'Afrique du Nord avaient des niveaux de fécondité plus faibles au début de la période, et ceux-ci continuent de baisser.

Ces évolutions contrastées de la fécondité sur le continent sont la résultante de progrès différents en matière de déterminants de la fécondité, notamment les variables intermédiaires directement liées à la procréation (âge au premier mariage, recours à la contraception moderne, infertilité…) et les variables socio-culturelles telles que l'urbanisation et l’éducation, ainsi que les politiques en matière de population. L'ancrage des politiques de planification familiale (date de démarrage et engagement au niveau politique) est une dimension importante de cette évolution. Toutefois, les progrès dans l’éducation, notamment celle des femmes, particulièrement au-delà du primaire - un facteur important de l'autonomisation de la femme - est l'un des déterminants majeurs des changements dans les comportements procréateurs, notamment en matière d’âge d'entrée en union et d'utilisation de la contraception moderne.

# Research article: Mobilisation citoyenne et politique en faveur des DSSRAJ en Afrique de l'Ouest: comment soutenir l'action de la société civile?

**Published:** 2021-11-30

**Authors:** Fatima Kandine, Sané Ndiaye

**Affiliations:** 1 Organisation ouest africaine de la santé, 01 BP 153 Bobo-Dioulasso 01, Burkina Faso; 2 Association Malienne pour la Protection et la Promotion de la Famille (AMPPF)

**Keywords:** Société civile, Accompagnement, Empowerment, Redevabilité, Jeunes, Afrique de l'Ouest

La communication portera sur un mécanisme de financement de la société civile de l'Organisation ouest africaine de la santé soutenant des actions de mobilisation citoyenne et politique de défense des droits sexuels et reproductifs (DSSR): FONDEMSAN. En quoi ce mécanisme est-il audacieux? En quoi appuie-t-il la réalisation de l'approche par les droits? Quels en sont les effets après trois ans de mise en œuvre?

Pourquoi miser sur les organisations de santé communautaires (OSC)?

L'engagement des OSC constitue un élément décisif pour l'amélioration durable des DSSR. En effet, elles jouent un rôle central dans l’évolution des politiques publiques en identifiant les besoins des populations et en les relayant auprès des différentes autorités. Elles jouent également un rôle de vigie en suivant de près la mise en œuvre des engagements pris par les États ainsi que la qualité des programmes. Elles constituent par ailleurs des partenaires précieux tant dans le changement des normes sociales que dans la mise en œuvre des plans et programmes gouvernementaux.

Comment accompagner leur développement et leur pérennisation?

Pour remplir leurs différents rôles, les OSC sont confrontées à trois défis majeurs:

1. Le manque de temps et d'outils pour élaborer des projets innovants.
2. Savoir appréhender les changements sociaux et politiques intervenus pendant le temps du projet.
3. Assurer leur développement organisationnel pour inscrire leur action dans la durée.

L'innovation de FONDEMSAN repose sur la mise en place d'un mécanisme d'accompagnement de ces trois aspects: l'appui à l'innovation, à la gestion des apprentissages et au développement organisationnel. Depuis sa création, ce fonds a permis de toucher 23 OSC organisées en consortium au Burkina Faso, Bénin, Guinée, Mali et Niger. La communication reviendra sur les apprentissages tirés de trois années d'accompagnement personnalisé.

Pour quels projets et quels changements?

Les projets portés par les OSC bénéficiaires de FONDEMSAN font le pari de mettre en pratique l'approche par les droits et le principe de redevabilité en matière de DSSRAJ. Partant du modèle socio-écologique du changement, et dans le but de faire évoluer les politiques et programmes nationaux de DSSRAJ, les projets financés combinent actions d'empowerment et de participation des jeunes avec construction de dialogues structurés entre autorités et citoyens/citoyennes. La communication présentera notamment l'un des projets financés au Mali ayant permis la création d'une plateforme en ligne de suivi de la mise en œuvre des engagements: le Layidu-mètre.

# Research article: Connaissances attitudes et pratiques des adolescents et jeunes dans les départements du Couffo et de l'Atlantique au Bénin

**Published:** 2021-11-30

**Authors:** Yassinmè Elysée Somasse

**Affiliations:** Enabel, Lot A1, Quartier Les cocotiers, 02 BP 8118 Cotonou, Bénin

**Keywords:** Connaissances, Droits sexuels et reproductifs, Tolérance, Genre, Diversité sexuelle, Adolescents, Jeunes, Bénin, Afrique subsaharienne

Au Bénin, les indicateurs de la santé sexuelle et reproductive des adolescents et jeunes ne sont pas reluisants. Pour contribuer à redresser la situation le programme santé de l'Agence belge de développement a lancé une étude afin d’évaluer les connaissances, attitudes et pratiques des adolescents et jeunes de 10-24 ans en matière de santé sexuelle et reproductive dans les départements de l'Atlantique et du Couffo.

Nous avons mené une enquête transversale avec un sondage aléatoire stratifié par commune et à deux degrés: les unités primaires étant les établissements scolaires et centres d'apprentissages, les unités secondaires, les classes. Les données ont été recueillies avec un questionnaire structuré digitalisé sur KoboCollect et administré en face-à-face aux sujets de 10-24 ans. Une analyse de régression logistique multivariée a été réalisée pour déterminer les facteurs associés à la connaissance des droits sexuels et de la physiologie reproductive ainsi qu’à l'attitude à l’égard du genre et des différences de sexualité.

Sur 1 038 adolescents et jeunes enquêtés, seuls 18,6% avait une bonne connaissance des droits sexuels (évaluée sur une série de 21 questions relatives aux droits sexuels); 19,9% avait une bonne connaissance de la physiologie reproductive, 6,5% avait une bonne connaissance des IST et 65,2% connaissait les méthodes contraceptives. Au niveau des attitudes, 15,6% avait une attitude positive à l’égard de l’égalité genre, seuls 7,5% avait une bonne tolérance à l’égard de l'homosexualité et des LGBTQI et 9,8% avorteraient une grossesse non désirée. Un peu moins de la moitié des sujets (46,3%) avaient un partenaire sexuel au moment de l'enquête dont 15% deux partenaires ou plus, 42,3% avaient déjà utilisé une méthode de contraception moderne mais dans 94,1% des cas, il s'agissait du préservatif masculin. La fréquentation d'un centre convivial (Ora= 2,5 p<0,001), une discussion entre amis sur la sexualité (Ora=2 p=0,001) et l'accès régulier à internet (ORa=2 p=0,01) étaient associés à une meilleure connaissance des droits sexuels. L'accès régulier à internet était associé à une meilleure tolérance des différences de sexualités (ORa =3,3 p<0,001), de même que l'appartenance au sexe féminin (ORa=2,0 p=0,009).

Les adolescents et jeunes des deux départements enquêtés ont une faible connaissance des droits sexuels, de la physiologie reproductive et des IST. La tolérance à l’égard des différences de sexualité est très faible et l'utilisation des méthodes de contraception doit être améliorée pour lutter contre les grossesses non planifiées.

# Research article: Assistance médicale à la procréation pour les couples africains en France

**Published:** 2021-11-30

**Authors:** Sylvie Epelboin

**Affiliations:** Hôpital Bichat - Claude-Bernard, 46 rue Henri-Huchard, 75018 Paris, France

**Keywords:** Assistance médicale à la procréation, Stérilité, Représentations culturelles, Anthropologie de la parenté, Afrique subsaharienne, France

La souffrance des couples en mal d'enfant est ubiquitaire, mais s'exprime de façon diverse selon la culture d'origine. L'expérience clinique au sein d'un centre d'Assistance Médicale à la Procréation (AMP) parisien dont les prises en charge concernent, pour un tiers de l'activité, des couples originaires d'Afrique subsaharienne, à parité d'Afrique de l'Ouest ou centrale, permet de mettre en exergue quelques spécificités de leur parcours d'AMP, tant sur le plan de son vécu que celui de considérations médicales ou économiques. Cette réflexion s'appuie également sur une expérience africaniste depuis 1972, dont un terrain de thèse sur les représentations de la fertilité des femmes Peules Bandé au Sénégal Oriental.

Les spécificités du parcours d'AMP combinent de multiples facteurs, dont l'association est pertinente, alors qu'aucun d'entre eux ne caractérise isolément la situation. De façon non exhaustive, on peut les énumérer ainsi:

- Le projet d'enfant se conçoit souvent dans une perspective hypergénésique.
- Le fait de devenir parent représente la seule voie d'insertion et légitimité sociales et de rupture d'isolement pour les femmes migrantes en France.
- Le contexte de l'infection virale pour le VIH ou les hépatites B et C peut se surajouter à la problématique de stérilité, le projet procréatif étant une revanche sur la honte, la discrimination dont les femmes ont fait l'objet.
- La confrontation entre projet d'enfant, critères de stabilité du couple et encadrement réglementaire de la procréation assistée en France, génère bien des situations d'aporie entre les couples et les équipes soignantes.
- Les représentations traditionnelles de la conception, variables selon les cultures d'origine, mal connues ou mal comprises, conditionnent l'acceptation de l'annonce de la stérilité de l'homme, les choix thérapeutiques, la congélation embryonnaire, etc.
- L'acceptation d'une politique de transfert embryonnaire mesurée, visant à éviter les grossesses multiples, y compris gémellaires, est difficile dans un contexte culturel où la gémellité est synonyme de chance, de richesse, de puissance de l'homme, de victoire sur le malheur / stérilité.
- Le recours au don de gamètes, ou à l'inverse la perspective du don d'embryons à un autre couple, se heurte à de multiples réticences, dont la crainte de rupture généalogique, à mettre en regard avec les systèmes de parenté et de dons de la société d'origine.

Ces représentations se confrontent à celles de la pensée culturelle des soignants, dont la prise de conscience de la réalité de la structuration de la « pensée traditionnelle » est éminemment variable.

# Research article: Grossesse et accouchement à haute altitude: avantage adaptatif de certaines populations – Un regard historique

**Published:** 2021-11-30

**Authors:** Dominique Jean

**Affiliations:** International Society for Mountain Medicine, 111 Rue Roy E, Montréal, Quebec, Canada

**Keywords:** Grossesse, Altitude, Hypoxie, Andes, Tibet

Au XVI^e^ siècle, quand les conquistadores fondèrent la ville de Potosi à 4000 m d'altitude, tous les nouveau-nés d'ascendance espagnole mouraient en période néo-natale. Les femmes espagnoles prirent l'habitude d'aller accoucher en basse altitude et ne revenaient à Potosi que quand l'enfant avait un an. Ce n'est que 53 ans après la fondation de la ville, soit plus d'une génération, que le premier enfant d'origine espagnole né à Potosi survécut. L'hypoxie d'altitude était certainement le facteur environnemental prédominant dans cette incapacité des immigrants à se reproduire sur place.

Les pathologies de la grossesse sont plus fréquentes en altitude, en particulier chez les femmes non acclimatées. Le principal risque est la prééclampsie, pouvant aboutir à une éclampsie, un hématome rétro-placentaire, une mort fœtale. L'altitude expose également à des difficultés d'adaptation néonatale.

La baisse du poids de naissance (PN) avec l'altitude est liée à un retard de croissance intra-utérin (RCIU) du 3^e^ trimestre, attribué à une majoration de l'hypoxie fœtale en altitude. Elle est en moyenne de 100 g par 1 000 m d'altitude et peut s'observer dès 1 500 m. Elle varie selon l'ancienneté d’établissement des populations en altitude, évoquant une adaptation au fil des générations: plus marquée au Colorado et chez les Han au Tibet, intermédiaire dans les Andes, absente chez les Tibétains.

Des mécanismes compensateurs existent chez la mère et le fœtus:

- L'hyperventilation maternelle d'origine hormonale, majorée en altitude, permet d'améliorer SaO_2_ et de préserver le contenu artériel en oxygène chez les femmes résidentes qui ont une Hb augmentée (Andes, Colorado). La concentration d'Hb n'est pas augmentée chez les Tibétaines mais elles ont des adaptations vasculaires plus marquées.
- La redistribution du flux sanguin pelvien vers les artères utérines augmente pendant la grossesse, mais elle est plus marquée et se maintient jusqu'au terme chez les populations anciennement établies en altitude, alors qu'elle diminue en fin de grossesse chez les populations plus récentes.
- L'index placentaire (rapport du poids placentaire au PN) est augmenté en altitude et la morphologie placentaire est différente: les villosités sont plus fines avec une plus grande densité de capillarisation, permettant d'améliorer l'apport d'O_2_ au fœtus. Dans les Andes, ces caractéristiques sont plus marquées chez les femmes d'ascendance indienne que chez celles d'ascendance européenne, suggérant une adaptation génétique.
- On observe au sang du cordon une augmentation de la concentration d'Hb ainsi que du pourcentage d'Hb foetale, d'affinité augmentée pour l'O_2_.

L'arrivée de la génomique permet d'avancer dans la connaissance d'adaptations différentes dans les populations andines et tibétaines.

# Research article: Prise en charge des hémorragies du post partum

**Published:** 2021-11-30

**Authors:** Jean-Eric Pontiès

**Affiliations:** École du Val-De-Grâce, 1 Pl. Alphonse Laveran, 75005 Paris, France

**Keywords:** Mortalité maternelle, Agonie utérine, Hémorragie de la délivrance, Kit de prise en charge des HPP, Organisation médico-chirurgicale des urgences du post-partum

La mortalité maternelle malgré des avancées encourageantes reste encore très élevée dans le monde.

Les pays en voie de développement paient 99% de ce tribut.

La connaissance des causes des hémorragies du post-partum, la précocité du diagnostic, la réaction adaptée de l’équipe médico-chirurgicale permet de réduire de 90% le taux de mortalité.

Ainsi, la délivrance dirigée, la révision utérine, l'utilisation d'ocytocine, la mise en place d'un ballonnet de Bakri, permettent dans 80% des cas de sauver la parturiente ou de mettre en place une stratégie thérapeutique soit par radiologie interventionnelle soit par une chirurgie d'urgence offrant des alternatives à l'hystérectomie d'hémostase.

L'apprentissage des personnels, l'organisation des salles de naissance, la mise en place d'un kit « hémorragies de la délivrance » sont des dispositions faciles à mettre en place, peu onéreuses et très efficaces pour parvenir aux objectifs de l'OMS de faire descendre la mortalité maternelle en dessous de 70 pour 100 000 naissances.

# Research article: Prise en charge des fistules vésico-vaginales obstétricales

**Published:** 2021-11-30

**Authors:** Ludovic Falandry

**Keywords:** Fistule vésico-vaginale, Fistule recto-vaginale, Incontinence urinaire, Procédures de chirurgie reconstructive

L'auteur ayant une expérience de plus de trois décennies des fistules obstétricales (FO) rappelle les principes de la prise en charge chirurgicale de la FO secondaire à un travail prolongé (classiquement plus de deux jours) cause principale des FO dans les pays pauvres.

Une classification clinique permet de classer ces fistules obstétricales en trois groupes de difficulté croissante. Si la technique de base est simple les difficultés dépendent des lésions tissulaires (sclérose), du siège de la fistule, des lésions associées, (atteinte sphinctérienne, fistules de la filière ano-rectale associées dans un tiers des cas). Si les FO du groupe I peuvent être traitées avec succès (moins de 20%) en un seul temps opératoire, les FO graves, complexes, du groupe II ou III nécessitent deux ou plusieurs interventions pour obtenir une guérison ou amélioration. La qualité des soins post-opératoires, la recherche clinique et chirurgicale permettront l'amélioration des résultats globaux, ce qui est encore loin d’être le cas dans les conditions actuelles. La prévention (soins obstétricaux d'urgence, ressources humaines) est possible puisque cette conséquence dramatique de la dystocie a pratiquement disparu dans les pays riches.

# Research article: Avortements provoqués en Afrique: un fléau persistant et majeur sur les décès maternel

**Published:** 2021-11-30

**Authors:** Patrick Thonneau

**Affiliations:** Université Senghor, Al Mansheyah Al Kubra, Qesm Al Mansheyah, Alexandria Governorate, Égypte

**Keywords:** Avortement, Décès maternels, Santé sexuelle et reproductive, Afrique

Plus de 240 millions de femmes sont enceintes chaque année, et un tiers de ces grossesses se terminera par une fausse couche, un enfant mort-né ou un avortement provoqué – ainsi, on estime à 60 millions le nombre annuel le nombre d'avortements.

L'OMS estime à 300 000 décès le nombre annuel de décès maternels, avec un taux global de mortalité maternelle de 211 décès maternels pour 100 000 naissances vivantes, variant entre 5 à 10/100 000 pour les pays industrialisés (Europe de l'Ouest, Amérique du Nord) a plus de 500/100 000 dans la plupart des pays d'Afrique subsaharienne – on peut dire que la probabilité de mourir d'une maternité est de 1 sur 25 dans les pays d'Afrique de l'Ouest (Tchad, Centrafrique, Mali), comparée à moins de 1/10 000 en Europe de l'Ouest.

Parmi les principales causes de décès maternels et après les hémorragies (un tiers), les hypertensions gravidiques (un quart), on retrouve les complications d'avortement qui représentent un sixième des cas de décès maternels. La très grande majorité des décès maternels ont lieu dans les pays en développement, avec une contribution particulièrement forte des pays d’’Afrique subsaharienne (on estime entre 15 et 20 000 le nombre de décès maternels annuels en Afrique qui sont liés à des complications d'avortement). En Afrique, le nombre annuel d'avortements estimé à 8,2 millions au cours de la période 2010–2014, est en hausse par rapport aux 4,6 millions estimés en 1990–1994, en raison de la croissance de la population des femmes en âge de procréer.

À partir de ces données épidémiologiques et démographiques, nous aborderons les aspects en lien avec les éléments déterminants que sont les grossesses non désirées et la disponibilité/acceptation des méthodes contraceptives, les avancées en matière de sécurisation des avortements et l'utilisation de nouvelles méthodes et, enfin, les aspects légaux et sociétaux.

# Research article: Projet NENGO: une initiative internationale d'envergure pour les victimes de violences sexuelles en République centrafricaine

**Published:** 2021-11-30

**Authors:** Florence Delcher

**Affiliations:** Fondation Pierre Fabre, Domaine d'en Doyse, route de Saint Sulpice, 81500 Lavaur, France

**Keywords:** Violences sexuelles, Soutien holistique, Autonomisations, Droits humains, République centrafricaine, Afrique subsaharienne

Inspiré d'un modèle préconisé et éprouvé en République Démocratique du Congo par le Dr Denis Mukwege, « l'homme qui répare les femmes », Prix Nobel de la Paix 2018, le programme NENGO (« DIGNITE » en langue Sango) a pour objectif de transformer la souffrance en force chez les victimes de violences sexuelles et basées sur le genre en République centrafricaine. Fondé sur un transfert de compétences entre les acteurs congolais et les acteurs centrafricains, NENGO a également pour objectif de faire de la sensibilisation et de la prévention auprès des populations. Ce programme de prise en charge globale des victimes est appuyé par l'Agence Française de Développement et mis en œuvre par un consortium de partenaires internationaux, dont la Fondation Pierre Fabre est cheffe de file avec sa double casquette de bailleur et d'opérateur sur le terrain.

NENGO propose aux victimes de Bangui et des provinces un parcours gratuit, coordonné et complet, grâce à la mise en place d'un « Guichet unique » ou « One Stop Center ». Le programme est construit autour de quatre piliers complémentaires:

- Médical: pour soigner les victimes de viols, d'agressions physiques et de problèmes gynécologiques graves (mutilations génitales, fistules, prolapsus).
- Psychologique: pour soutenir les victimes et les aider à dépasser leurs traumatismes.
- Socio-économique: pour accompagner les victimes vers l'autonomisation en les aidant à mettre en place une activité génératrice de revenus ou en leur donnant accès à l’éducation.
- Juridique: pour aider les victimes à obtenir justice.

# Research article: Dépistage et prise en charge du cancer du col de l'utérus en Afrique de l'Ouest

**Published:** 2021-11-30

**Authors:** Hassane Nouhou

**Affiliations:** Faculté des sciences et de la santé, Université Abdou Moumouni Niamey, Niger

**Keywords:** Cancer, Col de l'utérus, Dépistage, Niger, Afrique subsaharienne

Le cancer du col utérin est fréquent, de diagnostic tardif par manque de dépistage régulier dans la plupart des pays africains, pose un problème de santé publique. La connaissance de son histoire naturelle permet de le prévenir par dépistage. Plusieurs méthodes de dépistage existent, dont la plus connue et la plus courante est le frottis cervico-vaginal (FCV), mais relativement onéreux, non accessible à une majorité de femmes à bas niveau économique, vivant surtout en zones rurales. Il nécessite une logistique complexe, une formation avancée pour la pratique technique; or ces conditions ne sont réunies que dans les grandes villes des pays aux ressources limitées. En 2000, l'OMS et le CIRC en collaboration avec la Fondation Bill et Melinda Gates lançaient, dans une étude multicentrique, une méthode alternative au frottis: il s'agit d'une Inspection Visuelle du col utérin après application d'Acide acétique 5% (IVA) et de Lugol fort (IVL). Cette méthode apparaît moins onéreuse, de pratique facile, même en campagne de dépistage de masse. Les résultats de l'examen sont instantanés, basés sur l'aspect du col après application de l'acide acétique 5% et du Lugol. La sensibilité et la spécificité des tests sont d'environ 95%. Une biopsie est éventuellement pratiquée pour examen histologique. Le traitement consiste, selon le résultat histologique, soit en une électrocoagulation ou cryothérapie, une RAD/conisation, une chirurgie radicale ou une radiothérapie.

Le Niger et certains pays africains (Burkina Faso, Mali, Guinée Conakry, Congo Brazza) ont participé comme pays pionniers à cette étude multicentrique: formation préliminaire au CIRC (Lyon) de responsables sanitaires des pays.

L'expérience du Niger est présentée. Après la formation en 2001 de deux médecins au Centre international sur le cancer à Lyon, la formation de médecins et d'agents de santé a démarré à Niamey. De 2002 à 2006, près de 4 000 femmes ont été dépistées chaque année dans la capitale du pays. Puis de 2006 à 2009 les dépistages du cancer du col ont été étendus dans six régions du pays. Au total plus de 22 000 dépistages ont été effectuées. Durant cette période, plus de 700 lésions ont été diagnostiquées dont 26 cas de cancer invasif. Des traitements ont pu être proposées aux femmes dépistées (électrocoagulation, conisation chirurgie et radiothérapie).

# Research article: Impact de la Covid-19 sur la santé sexuelle et reproductive en Afrique

**Published:** 2021-11-30

**Authors:** Didier Ekouevi

**Affiliations:** Université de Lomé, Boulevard Gnassingbé Eyadema, 01BP1515 Lomé Togo

**Keywords:** Covid-19, Santé sexuelle reproductive, Lomé, togo, Afrique subsaharienne

La pandémie de Covid-19 a eu un impact dévastateur sur les systèmes de santé à travers le monde. Elle a également eu un impact sur le plan socio-économique, en limitant les moyens de subsistance et le bien-être de tous.

La Covid-19 touche les femmes et les hommes différemment. La pandémie aggrave les inégalités chez les femmes et les filles, ainsi que la discrimination chez les personnes vivant dans l'extrême pauvreté ainsi que d'autres groupes marginalisés.

Depuis le début de la pandémie, les projections des agences des Nations unies avertissent d'une limitation sévère de l'accès aux services essentiels de santé sexuelle et reproductive notamment ceux de la planification familiale et des consultations prénatales.

L'ONUSIDA signale une diminution de 5% des consultations prénatales et une diminution de l'accès des mères et des enfants aux services de santé essentiels, ce qui présage une hausse de la mortalité chez les enfants de moins de 5 ans. Des projections faites par modélisation signalent qu'une interruption des services de la PTME entraînerait une augmentation spectaculaire des nouvelles infections à VIH chez les enfants en Afrique subsaharienne (162% au Malawi, 139% en Ouganda, 103% au Zimbabwe et 83% au Mozambique).

Partout dans le monde, et particulièrement en Afrique les sages-femmes, les infirmières et les agents de santé communautaire sont indispensables pour contenir la Covid-19, tâche pour laquelle ils ont besoin de formation et d’équipement de protection individuelle. Les grossesses et les accouchements sûrs dépendent de tout ce personnel de santé, d'installations sanitaires adéquates et d'une stricte adhésion au protocole de prévention des infections. Les maladies respiratoires chez les femmes enceintes, en particulier celles attribuables à la Covid-19, doivent être traitées en priorité en raison du risque accru de survenue d'issue défavorable de grossesse. Dans le cadre la riposte contre la Covid-19, le maintien des services de soins de santé primaires est essentiel et fait partie intégrante de la stratégie de riposte définie par l'OMS. Les gouvernements doivent donc garantir un accès complet et sans entrave à tous les services de santé sexuelle et reproductive pour toutes les femmes et les filles.

# Research article: Les politiques en santé mondiale et la mortalité/morbidité materno-infantile civile?

**Published:** 2021-11-30

**Authors:** Joan Valadou

**Affiliations:** Ministère de l'Europe et des Affaires étrangère, Hôtel du ministre des Affaires étrangères, 37 quai d'Orsay, 75007 Paris, France

**Keywords:** Muskoka, Priorité santé mondiale, Actions de la France, Diplomatie sanitaire

La promotion de la santé des populations dont l'un des objectifs est l'amélioration de la santé maternelle, néonatale, infantile et des adolescents et adolescentes est une priorité de la France en santé mondiale. Les droits et santé sexuels et reproductifs constituent à cet égard une dimension essentielle de cette approche et de la diplomatie féministe de la France. La France mène dans ce contexte des actions concrètes en matière d'aide au développement à travers plusieurs programmes notamment le Fonds français Muskoka, le Fonds mondial pour les survivant(e)s de violences sexuelles liées aux conflits, ainsi que les programmes de l'AFD mis en oeuvre sur le plan bilatéral. Dans le cadre du Forum génération égalité (FGE), la France a pris la tête de la coalition d'action en faveur des droits et santé sexuels et reproductifs (DSSR) et s'est engagée à financer et soutenir plusieurs fonds, notamment: (i) *UNFPA supplies,* pour améliorer l'accès à des contraceptifs modernes, à la santé maternelle, (ii) un programme innovant pour favoriser un accès équitable aux produits de santé sexuelle et reproductive (*Shaping Equitable Market Access for Reproductive Health* – SEMA) ainsi que (iii) l'Organisation pour le dialogue en faveur de l'avortement sécurisé (ODAS) en Afrique de l'Ouest et du Centre portée par l'ONG Ipas. En 2022, la France renouvellera sa stratégie en santé mondiale, ainsi qu'en matière de DSSR et de genre. La santé et les droits à la santé des populations, y compris les DSSR, continueront à constituer une priorité pour la France.

# Research article: L'AFD et les droits et la santé sexuels et reproductifs

**Published:** 2021-11-30

**Authors:** Anne Roos-Weil

**Affiliations:** Agence française de développement (AFD), 5 rue Roland Barthes, 75 598 Paris Cedex 12, France

**Keywords:** AFD, DSSR, Aide au développement, Stratégie

Déclinant la stratégie française, l'AFD a fait de l'approche par les droits en matière de santé sexuelle et reproductive l'un des axes clés de son action en faveur de l'autonomisation des femmes.

Entre 2016 et 2020, l'AFD a octroyé 199 M€ à des activités en lien avec les droits et la santé sexuels et reproductifs, principalement dans les pays pauvres prioritaires de l'APD française, dont 52 M€ octroyés aux organisations de la société civile française et leurs partenaires locaux via le dispositif « Initiatives OSC ». Elle finance des projets et des acteurs qui agissent pour améliorer l'accès à des services de santé sexuelle et reproductive complets et de qualité avec un focus sur les jeunes et adolescents/adolescentes.

Historiquement engagée sur la santé sexuelle et reproductive dans le cadre du continuum de soins mère-enfant, l'AFD a progressivement élargi son champ d'intervention pour intégrer les enjeux des droits et la lutte contre les inégalités de genre dans ses interventions en lien avec le positionnement de plus en plus fort de la France sur ces enjeux. L'AFD structure son action autour de trois thématiques clés:

- Garantir une offre de services de santé sexuelle et reproductive complète et de qualité, adaptée aux adolescents et adolescentes;
- Promouvoir la demande et accompagner le changement de normes et de comportements;
- Lutter contre les violences basées sur le genre et améliorer leur prise en charge.

L'AFD accompagne les États dans l’élaboration et la mise en œuvre de leurs politiques nationales. Elle soutient les organisations de la société civile, notamment grâce au dispositif « Initiatives OSC », et contribue au renforcement de leurs capacités. Elle promeut également des approches régionales en appuyant des organisations interétatiques.

Le dialogue partenarial est au cœur de l'action de l'AFD qui collabore étroitement avec des organisations régionales, des partenaires financiers, de nombreuses organisations de la société civile ainsi que des instituts de recherche. La production des connaissances joue en effet un rôle essentiel pour accompagner le changement de normes.

# Research article: Les 10 ans du Fonds français Muskoka – bilan et perspectives

**Published:** 2021-11-30

**Authors:** Fleur Vernat

**Affiliations:** Fonds français Muskoka, Fonds des Nations unies pour l'Enfance – Unicef, Bureau régional pour l'Afrique de l'Ouest et du Centre, BP 29720 – Dakar, Sénégal

**Keywords:** Muskoka, SRMNIA-Nut (Santé Reproductive, Maternelle, Néonatale, Infantile, des Adolescents et adolescentes, et de la nutrition), FGE (Forum Génération Egalité), Partenariats, IHI (Interventions à Haut Impact)

Depuis 10 ans, le Fonds français Muskoka (FFM) œuvre pour améliorer la santé et le bien-être des femmes, des nouveau-nés, des enfants et des adolescents et adolescentes dans neuf pays d'Afrique de l'Ouest et Centrale: Bénin, Burkina Faso, Côte d'Ivoire, Guinée, Mali, Niger, Sénégal, Tchad et Togo.

Basé sur un mécanisme de coordination novateur maximisant les mandats de quatre agences des Nations unies (OMS, ONU Femmes, UNFPA et UNICEF), le FFM met en œuvre des Interventions a Haut Impact (IHI) basées sur une approche multisectorielle pour une réponse à la fois efficace et durable.

En matière de santé sexuelle et reproductive, les interventions du FFM incluent:

- le développement de services à base communautaire;
- la mise à disposition de gammes complètes de contraceptifs modernes;
- l'information sur la planification familiale et les questions de genre par des actions de sensibilisation via différents canaux (radio, TV, campagnes communautaires, mobilisation sociale);
- la mise en place de services de santé sexuelle et reproductive, incluant la planification familiale, en milieux scolaire et extrascolaire et le développement de l’éducation complète à la sexualité (ECS), et
- la sensibilisation aux violences basées sur le genre et aux inégalités de genre.

En dépit d'un contexte difficile, le FFM a ainsi permis d'atteindre des résultats remarquables depuis 10 ans. En voici quelques exemples en matière de santé sexuelle et reproductive plus spécifiquement:

- en Côte d'Ivoire, le FFM a contribué à la diffusion et à la supervision de cours d'ECS auprès de 71,4% des nouveaux élèves du secondaire, ainsi qu’à l'adoption d'un document national de référence sur la santé et l'hygiène menstruelles;
- au Togo, la prévalence de la contraception moderne est passée de 16,3% en 2012 à 23,2% en 2017. Le succès a été tel que le ministère de la Santé togolais a élaboré un Plan stratégique des interventions à base communautaire.

Les témoignages de nos bénéficiaires restent cependant les plus éloquents. Ainsi ce message de Roubatou Tchagaou, élève au Togo: « Au centre des jeunes, on m'a appris que j'ai des droits et qu’à 15 ans, je n’étais pas encore majeure pour me marier. Ils m'ont appris mes droits que je ne connaissais pas. Moi, ce que je connaissais, à la maison, c'est la soumission. Maintenant on va se marier au moment opportun. Ce n'est plus un mariage forcé ni un mariage précoce. Et grâce au centre, on va combattre les grossesses indésirées ».

# Research article: Le Forum génération égalité - Des engagements pour l'avenir

**Published:** 2021-11-30

**Authors:** Stéphanie Nadal

**Affiliations:** UNICEF WCARO, Fonds des Nations unies pour l'enfance Bureau régional Afrique de l'Ouest et Afrique centrale, Immeuble Maimouna III, Route de la Pointe des Almadies en face l'Hotel Meridien President Dakar, Sénégal

**Keywords:** Muskoka, SRMNIA-Nut (Santé Reproductive, Maternelle, Néonatale, Infantile, des Adolescents et adolescentes, et de la nutrition), FGE (Forum Génération Egalité), Partenariats, IHI (Interventions à Haut Impact)

Le Forum génération égalité (FGE) s'est déroulé à Mexico en mars, puis à Paris du 30 juin au 2 juillet 2021.

Organisé autour de six coalitions d'action, ce Forum a lancé un plan d'action quinquennal pour réaliser des progrès irréversibles en matière d’égalité entre les femmes et les hommes qui repose sur une série d'actions concrètes, ambitieuses et transformatrices et comprend des engagements financiers pour un montant de 40 milliards de dollars.

Dès avant l'ouverture du FGE, le Président Macron mettait en avant les résultats du FFM sur son compte Twitter en tant qu'exemple des réalisations concrètes que la politique de coopération internationale et de développement de la France permettent de réaliser. À ce titre, il est important de rappeler que le Président Macron a identifié le FFM comme outil de mise en œuvre privilégié de la grande cause de son quinquennat (l’égalité entre les femmes et les hommes).

Dans le cadre de la coalition d'action sur la « Liberté à disposer de son corps et la santé et les droits sexuels et reproductifs », le FFM a soumis trois engagements en matière de Communication pour le changement social et comportemental (CCSC).

La France a également dédié un évènement de haut niveau au FFM intitulé « Muskoka: 10 ans d'action pour la santé des femmes et des enfants » le 1^er^ juillet. Celui-ci aura vu la participation de quatre ministres (France, Guinée et Togo) et permis à la France d'annoncer le renouvellement de son engagement jusqu’à fin 2025 et à hauteur de 50 millions d'euros.

À la fin du FGE, le ministère de l'Europe et des Affaires Étrangères a de nouveau fait référence au FFM dans un dernier tweet indiquant que la France consacrerait 260 M€ aux DSSR sur les cinq prochaines années dans le cadre de sa politique de développement solidaire et du Fonds français Muskoka.

# Research article: Renforcement des capacités des coiffeuses sur les méthodes de planning familial de courte durée au Mali

**Published:** 2021-11-30

**Authors:** Assitan Baya Sidibe

**Affiliations:** Direction régionale de la santé, Sikasso, 8878+QW2, Sikasso, Mali

**Keywords:** Renforcement, Coiffeuses, Planning familial, Mali, Afrique subsaharienne

La planification familiale (PF) reconnue depuis longtemps en Afrique subsaharienne comme étant un moyen essentiel pour maintenir la santé et le bien-être des femmes et de leur famille.

Le Mali a adhéré aux mouvements mondiaux en plus de ses politiques internes, pour l'amélioration de la santé maternelle et infantile, tels que la Campagne d'accélération de la réduction de la mortalité maternelle et néonatale en Afrique (CARMMA), le Partenariat de Ouagadougou (PO) et l'Initiative FP2020, dans le but de garantir une meilleure accessibilité fondée sur les droits à des services PF de qualité.

Aussi dans le cadre du repositionnement de la PF, sur le plan international un accent particulier a été mis sur la Jeunesse à la conférence de Londres consacrée à la planification familiale.

Dans le domaine de la santé de la reproduction des jeunes de moins de 18 ans, soit 53% de la population au Mali, la situation est alarmante. Le mariage précoce et l’éducation sexuelle insuffisante constituent de grands défis entraînant des taux élevés de grossesses précoces, des avortements clandestins et de nombreux décès.

Par ailleurs, les services de SR/PF ne sont pas adaptés aux jeunes la plupart du temps. L'accès des jeunes aux services et produits PF demeure un défi en raison de l'accès limité aux outils de communication sociale pour le changement en matière de PF. À ces facteurs, on peut associer la persistance des pesanteurs socioculturelles et religieuses, la faiblesse du dialogue communautaire sur la PF, le manque de dialogue parents-enfants et enfin le faible plaidoyer en faveur des droits en santé sexuelle et reproductive des jeunes.

Selon la sixième enquête démographique et de Santé Mali 2018, l'utilisation des méthodes de contraception moderne est de 16%.

La région de Sikasso, deuxième région peuplée où les indicateurs PF sont aussi faibles tant dans l'utilisation des services (19,2%) qu'au niveau des besoins non satisfaits (23%).

Face à ces nombreux défis, le Mali a développé son Plan d'Action Nationale Budgétisé (PANB 2019-2023) qui prend en compte tous les défis identifiés et les engagements régionaux et internationaux dont l'objectif annoncé dans ce plan est 30% vers l'horizon 2023.

C'est ainsi qu'au niveau régional avec l'appui du Fonds français Muskoka, un plan de travail annuel est élaboré par l'ensemble des acteurs du secteur (santé, développement social et promotion de la femme) et l'une des activités innovantes est le renforcement des capacités des coiffeuses sur les méthodes de planning familial de courte durée pour booster l'utilisation des services PF par les jeunes.

# Research article: L'approche des espaces sûrs à Koutiala, région de Sikasso, Mali

**Published:** 2021-11-30

**Authors:** Boubacar Traore

**Affiliations:** Association de Soutien au Développement des Activités de Population (ASDAP), J333+HPG, Bamako, Mali

**Keywords:** Espaces sûrs, Santé reproductive, Genre, Mali, Afrique subsaharienne

À l'instar du reste du Mali, dans la région de Sikasso certaines pratiques traditionnelles empêchent les femmes de jouir de leurs droits. Le choix de Koutiala s'est justifiait par un fort ancrage culturel de ces pratiques, la forte croyance aux mythes et rumeurs, la méconnaissance des femmes et des filles de leurs droits; la persistance des tabous sur la sexualité, la forte résistance au changement.

L'approche des espaces sûrs, basée sur des espaces sécurisés d’échanges et d'apprentissage où la parole est libre entre un mentor femme et des filles de 10 à 24 ans organisées en petits groupes, en est une réponse. Elle a pour objectifs d'améliorer l'accès aux informations et services de santé de qualité, développer les compétences de vie courante, et favoriser l'autonomisation économique par l'apprentissage de métiers. Les composantes sont: santé sexuelle et reproductive; genre et droits humains; compétences de vie courante; soutien scolaire; alphabétisation et gestion simplifiée en entreprenariat. Puis l'initiative des Clubs des maris et futurs maris a été introduite afin d'offrir aux jeunes maris et futurs maris des connaissances et compétences sur l’égalité des genres et la masculinité positive. Ce manuel aborde six thématiques, la dynamique du genre et du pouvoir, les droits de santé sexuelle et reproductive, la prévention de la violence basée sur le genre et la répartition de tâches non-rémunérées. L'animation est faite en 40 semaines pour les filles et 20 pour les garçons. Les bénéficiaires indirects sont les autres membres de la communauté à travers les activités de communication.

Des résultats obtenus depuis 2018:

- 24 espaces sûrs pour les filles et les garçons;
- 3 466 mentorées, 532 maris et 700 futurs maris ont terminé les sessions, soit respectivement 96,28%, 100% et 97,63%;
- 296 mentorées candidates à l'examen ont bénéficié de soutien scolaire avec un taux de réussite de 65,54% supérieur aux taux local 56,31%;
- 32 032 personnes touchées;
- 835 nouvelles et 1 839 anciennes utilisatrices de planning familial.

Cependant, des défis demeurent, le retard dans l'enrôlement et aussi le faible taux de fréquentation des bénéficiaires pour cause d'activités domestiques.

Les perspectives sont de formaliser les nouvelles compétences des filles par la formation professionnelle; de soutenir les facteurs d'autonomisation économique et sociale, et de faire d'elles et des membres des clubs, des champions du changement dans leurs communautés en relayant le concept des espaces sûrs et de la masculinité positive.

# Research article: L'Edutainment, une approche innovante pour surmonter les défis en matière santé et de bien être en Afrique

**Published:** 2021-11-30

**Authors:** Mbathio Diaw

**Affiliations:** RAES, 118 rue Loulou, Fann-Hock Dakar, Sénégal

**Keywords:** Éducation par le divertissement, Edutainment, Santé, Bien-être, Afrique

Malgré les progrès obtenus au cours des dernières années en Afrique de l'ouest et du centre sur la mobilisation en faveur de la Santé et des Droits Sexuels et Reproductifs, le développement de stratégies efficaces, englobantes et innovantes reste encore prégnant pour (i) améliorer l'offre et la demande de services en SSR pour les adolescentes, les jeunes et les femmes, (ii) contribuer à la transformation des normes, attitudes et pratiques sociales qui font obstacle à la réalisation des droits de ces jeunes, iii) favoriser un plaidoyer local et régional porté par les populations elles-mêmes avec l'implication de toutes les catégories d'acteurs.

L’éducation par le divertissement, utilisé par le RAES, a connu un regain d'intérêt croissant afin de compléter les interventions classiques dans le champ de la promotion de la santé. Cette approche a montré des résultats prometteurs en Afrique sur les thèmes du planning familial, de la santé sexuelle des ados et des jeunes, des violences basées sur le genre, du VIH, de la gouvernance démocratique ou encore plus récemment de la Covid-19.

Cette stratégie d'edutainment permet de renforcer les connaissances avec du contenu informatif culturellement adapté et scientifiquement juste, de favoriser une prise de conscience individuelle et collective, d'engager des débats communautaires ou politiques pour mettre en place des actions et solutions locales de Droits, de justice et de santé, de plaider pour un bien-être, un ancrage du genre et de l’égalité des sexes. L'objectif de ces campagnes est d'amener les communautés, l’État, les prestataires de santé, les décideurs, les OSC, le secteur privé à croiser le regard et s'engager vers des dynamiques de transformation durables.

Ce processus SBCC passe par de la recherche formative, la création de contenus et messages culturellement adaptés alimentant des campagnes médias, digitales ou communautaires pour faire face aux défis identifiés et faire évoluer les normes sociales par les populations elles-mêmes.

Cela passe aussi par un dispositif de suivi, évaluation et capitalisation rigoureux pour tirer des leçons des campagnes et mesurer les effets sur les connaissances et attitudes des cibles.

Force est de reconnaître que l'approche de l'Edutainment est adaptée au public africain utilisant les médias classiques, le digital et les espaces communautaires. Tous ces différents canaux sont utilisés par le RAES pour créer le changement par et pour les communautés.

Le RAES s'ouvre aujourd'hui aux espaces de mobilisation populaire comme le sport pour encore favoriser la mobilisation sociale et politique pour le changement.

# Research article: Promotion des droits et de la santé sexuels et reproductifs des jeunes et des adolescents et adolescentes au Niger

**Published:** 2021-11-30

**Authors:** Aurélie Musca Philipps, Mahamoudou Balarabé, Roubanatou Abdoulaye-Mamadou

**Affiliations:** Solidarité thérapeutique et initiatives pour la santé (Solthis), 34 Avenue Jean Jaures - 75019 Paris - France

**Keywords:** Jeunes, Adolescents, Droits, Santé, Empowerment, Niger, Afrique subsaharienne

Au Niger où près de 70% de la population a moins de 25 ans, l'amélioration de la santé sexuelle et reproductive des adolescents/adolescentes et jeunes (SSRAJ) est un enjeu important. Le pays se caractérise par une faible prévalence contraceptive, un taux important de grossesses précoces et une incidence du VIH supérieure chez les jeunes à celle de la population générale. Les représentations socioculturelles défavorables concernant la sexualité des jeunes et adolescentes contribuent à entraver l'accès à l'information et aux services pour ces publics. L'offre de services peu adaptée, souffre d'un manque de ressources et de formation des soignants/soignantes.

Le projet Jeunes et adolescents/adolescentes en Santé mis en œuvre à Niamey et Maradi par Solthis en partenariat avec Lafia Matassa et Équilibre et Population, vise à améliorer l'accès des adolescents/adolescentes et jeunes à des services de SSR de qualité et à renforcer leurs capacités d'agir.

Un diagnostic participatif a permis de construire avec les soignants/soignantes et les usagers/usagères, les actions à mettre en œuvre. Une étude baseline menée auprès de 841 jeunes et adolescents/adolescentes a confirmé le faible recours aux services et les connaissances limitées en SSR des adolescents/adolescentes et jeunes. Seuls 2,97% des personnes interrogées avaient fréquenté un centre au cours de l'année précédente. Les principales barrières mention-nées étaient le mauvais accueil et le manque de confidentialité. Un score de qualité défini avec les autorités sanitaires et utilisé dans les structures de santé a permis de mieux comprendre les enjeux en lien avec l'organisation, les pratiques et la relation de soins et a révélé un score moyen de base de 52/100.

Face à ces constats, le projet met en œuvre différentes actions pour améliorer la qualité des services, le recours aux soins et l'accès à l'information: ateliers d’échanges autour des représentations en lien avec la SSRAJ, sensibilisation aux inégalités de genre, renforcement de compétence des soignants/soignantes dans une approche globale du continuum de soins en SSR, stratégie communautaire et implication des adolescents/adolescentes et jeunes. Les premières for-mations se sont concentrées sur l'approche par les droits, les caractéristiques de services de SSRAJ adaptés, la contraception et la réponse aux violences basées sur le genre chez les adolescents/adolescentes et jeunes.

Le projet se déroule sur trois ans, les premières actions ont permis une augmentation de 6% du score de qualité dans les structures soutenues à Niamey. Des améliorations ont en particulier été observées sur les pratiques de soins et l'accueil des usagers/usagères.

# Research article: Promouvoir l'accouchement en maternité accréditée pour réduire la mortalité maternelle au Mali

**Published:** 2021-11-30

**Authors:** Hubert Balique

**Affiliations:** Faculté de Médecine de Marseille, 34 place Jean Jaurès, 13001 Marseille, France

**Keywords:** Mortalité maternelle, Accouchement sécurisé, Maison des mamans, Accès aux soins, Mali, Afrique subsaharienne

Depuis plus de 50 ans, des efforts considérables ont été entrepris dans la lutte contre la mortalité maternelle. Pourtant, les résultats en Afrique subsaharienne, dont a témoigné l’évaluation des OMD en 2015, ne sont à la hauteur ni de la multiplication des projets conduits par les Etats, à travers bien souvent l'engagement d'ONG, ni de l'importance des sommes mobilisées par la communauté internationale, malgré des résultats ponctuels certains. Pourtant, la protection de la vie des mères et de leurs enfants reste encore une des préoccupations majeures des familles, qui n'ont pas identifié dans les actions entreprises les réponses à leurs aspirations. L'analyse de cette situation conduit à mettre un accent particulier sur l'accouchement en maternité accréditée, qui nécessitera la maîtrise de la qualité des soins et la suppression de toute barrière financière à l'accès aux soins des usagers.

En zone rurale, ces deux mesures devront être complétées par la présence effective des femmes à proximité de la maternité avant le début du travail pour réduire les risques, déceler immédiatement la survenue de problèmes et intervenir avec l'efficacité requise que ce soit localement ou après référence. Dans cette perspective, doit être promue la création et la gestion appropriée de maisons des mamans gérées par une association locale de femmes, à condition que la maternité réponde aux critères de qualité requis et que le tarif des soins soit en rapport avec les capacités financières de toutes les familles, y compris les plus défavorisées. Cette mesure nécessitera une forte implication des femmes, avec l'appui de leurs maris, à la fois pour obtenir l'adhésion des familles et gérer l'accueil des femmes pendant la période, qui précède l'accouchement. Le succès d'une telle option stratégique nécessite (i) un engagement affirmé du ministre de la Santé, (ii) une déverticalisation de la lutte contre la mortalité maternelle et son insertion dans un programme national de renforcement du système de santé, (iii) un mouvement des professionnels de santé, en particulier des médecins et des sages-femmes en direction des zones rurales et des quartiers périurbains, (iv) la mise en place d'un mécanisme de financement basé sur les résultats, permettant notamment de réaliser l’équilibre des comptes de chaque structure de santé, quelle que soit sa localisation géographique. La maison des mamans de Boni au Mali montre la faisabilité technique et les résultats de cette nouvelle approche stratégique dans une des zones qui figurent parmi les plus défavorisées du pays.

# Research article: Quatre ans d'expérience d'un service de santé reproductive au Cameroun et encore une longue route à parcourir

**Published:** 2021-11-30

**Authors:** Sandrine Nyotue, Aimé Assigui, Laura Ciaffi, Franck Wanda, Eric Comte, Vanessa Christinet

**Affiliations:** Centre international de recherches, d'enseignements et de soins (CIRES), BP 11 Akonolinga, Cameroun

**Keywords:** Santé sexuelle, Cancer du col, District rural, Cameroun, Afrique subsaharienne

Depuis 2017, le CIRES (Centre international de recherche d'enseignement et de soins) a ouvert au sein de l'Hôpital de district d'Akonolinga (zone semi-rurale à 110 km de la capitale), un service de santé sexuelle ciblant les femmes du district pour tous les besoins au dehors de la grossesse: infections sexuellement transmissible (IST), planning familial, dépistage du cancer du col de l'utérus, dépistage du VIH et informations sur l'hygiène de menstruation.

En quatre ans de fonctionnement nous avons reçu un total de 3 275 de femmes venant de l'ensemble du district de santé parmi lesquelles 2 775 (85%) étaient en âge de procréer. Les raisons les plus fréquentes de consultation ont été le dépistage du VIH et le dépistage du cancer du col de l'utérus.

Nous avons dépisté 904 femmes pour le VIH et 1 038 pour le cancer du col. Un total de 593 MST ont été diagnostiquées et nous avons approvisionné 1 228 femmes en contraception (dont 349 méthodes à longue durée d'action).

Malgré l'offre variée et très accessible en termes de prix, le service est sous-utilisé avec une couverture qui ne dépasse pas le 31% de la population cible.

Notre expérience montre que:

1. Les activités décentralisées en communauté menées en coordination avec les responsables des aires de santé et leurs mobilisateurs communautaires proposant une offre de soins variée et intégrée a été la stratégie la plus efficace pour atteindre notre population cible.
2. Le dépistage du cancer du col n'est pas une priorité pour les femmes de la zone, la méconnaissance des possibilités de prise en charge avec un diagnostic précoce n’étant pas connues. Il y a encore un besoin énorme d’éducation, communication et information.
3. La gratuité des services est très importante pour augmenter l'acceptation des activités préventives.
4. Le personnel de santé est aussi peu informé sur l’évolution des modalités de dépistage et traitement des lésions précancéreuses de l'utérus
5. Une offre intégrée de plusieurs services est fondamentale pour répondre à des exigences très différentes de la population féminine et masculine sur la sexualité.

Au Cameroun en 2021 les besoins en termes de santé sexuelle pour la population rurale restent non couverts et les barrières à un service efficace et intégré sont nombreuses. Une coopération entre personnel de santé et communauté est nécessaire pour mieux adapter l'offre de soins à la demande la communauté.

# Research article: Intérêt des équipes médicales mobiles en santé materno-infantile

**Published:** 2021-11-30

**Authors:** Patrick Dewavrin, Ibrahima Faye, Francis Klotz

**Affiliations:** Le Kaïcedrat, 43 rue des épis d'or, 91210 Draveil, France

**Keywords:** Équipe mobile, Planning familial, Mortalité maternelle, Mortalité infantile, Sénégal, Afrique subsaharienne

Les populations enclavées d'Afrique sont celles qui présentent les plus mauvais indicateurs sanitaires. L'immensité des territoires, la dispersion de l'habitat et l'absence de réseau routier, rendent l'accès aux soins difficiles. Au-delà de 15 km d'un poste de santé (2 heures de charrette), la demande de soins chute de 50% (source Santé Sud).

Certaines zones très éloignées sont de véritables déserts sanitaires.

Pour répondre à ce défi, à côté de ses équipes mobiles médicales classiques, l'association Le Kaïcedrat a mis en place en 2015 au Sénégal oriental une équipe médicale mobile légère attachée à un poste de santé publique.

Une sage-femme avec un chauffeur en véhicule 4X4, part consulter tous les matins dans 20 villages distants de 15 à 60 km du poste de santé. Chaque village dispose d'une case de santé, construite gratuitement par la population et tenue par un agent de santé. Il diagnostique et traite le paludisme, prépare les consultations de la sage-femme et dispense des séances d’éducation sanitaire. Ce dispositif a été évalué à deux ans d'intervalle, en 2015 à son démarrage, puis en 2017, après deux ans de fonctionnement.

La population étudiée représente 5 000 habitants, 1 300 femmes en âge de reproduction et 1 000 enfants de moins de 5 ans.

Les résultats ont été les suivants:

- le nombre de femmes enceintes médicalement suivies a augmenté de 100%;
- la mortalité infantile a chuté de 60%, à 30/1 000, se situant en dessous du taux national;
- le nombre d'enfants complètement vaccinés est passé de 31% à 66%;
- le nombre d'enfants bénéficiant d'un allaitement maternel exclusif a augmenté de 170%;
- le nombre d'enfants inscrits à l’état civil est passé de 16 à 32%;
- le nombre de femmes adhérant au planning familial est passé de 15 à 42 (+ 180%); il est actuellement, en 2021, de 101 femmes (+ 500%);
- aucun de décès maternel n'a été constaté pendant ces deux années.

En revanche peu d'améliorations ont été obtenues sur le taux d'accouchements en maternité. 80% des accouchements ont continué à être effectués au village par des matrones.

Ce dispositif présente un coût de fonctionnement de 20 000 € par an soit 4 euros par an et par habitant.

Notre présentation, décrit le déroulement de cette étude et les enseignements que nous en avons retirés.

# Research article: Gestion des violences basées sur le Genre (VBG) lors de la riposte à la Covid-19 dans le grand Abidjan de mars 2020 à mai 2021

**Published:** 2021-11-30

**Authors:** Solange Amethier, Sita Kroman Savané, Baba Sangaré Baba, Djibril Chérif, Florence Kadjo, Daouda Coulibaly, Joseph Bénié Bi Vroh Joseph

**Affiliations:** Instance de coordination nationale de Côte d'Ivoire du Fond mondial pour la lutte contre le VIH, la tuberculose et le paludisme, CCM-CI, Abidjan, Cocody Riviéra Golf, Côte d'Ivoire

**Keywords:** Covid-19, Sujet contact, VBG, Multisectorielle, Abidjan, Côte d'Ivoire, Afrique subsaharienne

Le Centre des opérations d'urgence de santé publique (COUSP) a fait intervenir les travailleurs sociaux du ministère de la Femme, de la Famille et de l'Enfant dans la riposte à la pandémie à coronavirus Covid-19. Cette collaboration multisectorielle initiée dans le but d'assurer une bonne adhésion des populations aux implications sanitaires liées aux mesures sociales de santé publique a-t-elle fourni des résultats dans le suivi des contacts et la prise en charge psychosociale des sujets affectés par la Covid-19?

Nous avons mené une enquête transversale descriptive de mars 2020 à mai 2021 portant sur les activités de deux pools de travailleurs sociaux mis en place afin de collaborer avec les équipes d'intervention rapide et de renforcer les équipes de suivi des contacts au niveau des districts sanitaires d'Abidjan. Ces activités ont été coordonnées par un troisième pool chargé de la coordination et de l'appui technique des équipes.

Les activités menées ont porté essentiellement sur la sensibilisation des populations, les visites à domicile, les écoutes, la gestion des cas de violences basées sur le genre (VBG), la prévention et le traitement des cas de stigmatisation et de discrimination, l'accompagnement psychosocial et l'orientation des ONG et associations sur les mesures barrières et les idées reçues sur la Covid-19. De mars 2020 à mai 2021, concernant les violences basées sur le genre (VBG), les équipes ont enregistré et suivi 58 cas de viols, 7 agressions sexuelles et 177 agressions physiques, 125 situations de violence psychologique/émotionnelle, 35 cas de maltraitance et 7 mariages forcés. Une augmentation du nombre de violences a été relevée durant la pandémie à Covid-19. Les travailleurs sociaux ont assuré pour ces cas la prise en charge psychosociale qui repose sur l’écoute et l'identification des dangers imminents, l’évaluation des besoins et l’élaboration d'un plan d'action avec la survivante. Une prise en charge juridique et judiciaire a parfois été initiée. Cette activité a permis de relever des bonnes pratiques de collaboration et d'optimisation des ressources. Cependant, elle a été confrontée à des difficultés notamment l'insuffisance des moyens de communications pour le suivi des cas contacts, l'appui en vivres et non vivres aux familles Covid-19 en confinement et la coordination entre les structures de santé et sociales.

La pandémie à Covid-19 a été marquée par une augmentation des cas de violences dont la prise en charge adéquate a permis d'en limiter les conséquences.

# Research article: Prise en charge des fistules obstétricales au Burkina Faso de 2010 à 2018: un exemple de partenariat franco-burkinabé

**Published:** 2021-11-30

**Authors:** Gilles Karsenty, Jean Charles Alimi, Quentin Alimi, Jean Marie Colas, Fréderic Thoreau, Michèle Loubat, Antoine Ottomani, Valérie Pellegrin, Geraldine Rebuffat, Marie Karsenty Poisson, Laurence Bruder, Alice de Besse de La Roumiguiere, Patricia Sana, Régis Deretz, Olivier Paul, Aristide Fasnewinde Kaboré

**Affiliations:** Mères du monde en santé. MMS-France

**Keywords:** Fistules obstétricales, Santé maternelle, Suivi obstétrical, Accouchement voie basse, Césarienne, Incontinence urinaire, Burkina Faso, Afrique subsaharienne, France

Les fistules obstétricales (FO) sont endémiques dans les pays à faible revenus (<1 035 dollars/pers/an) malgré leur reconnaissance par l'OMS (Campagne End Fistula). Elles témoignent des carences des politiques de santé materno-infantile. Une ONG chirurgicale française (EFE-BF rebaptisé aujourd'hui MMS-France) a collaboré pendant 8 ans avec des soignants Burkinabés pour la prise en charge des patientes atteintes de FO dans la province des Bâlets (district de Bo-romo). Nous présentons les résultats obtenus et retraçons les étapes (succès et échecs) de ce parcours.

Nous avons analysé la base de données alimentées par chacune des missions organisées sur la période en termes de socio-démographie des patientes, de type de fistule, d’étiologie, de techniques chirurgicales proposées et de résultats. Nous avons retracé également les modes de financement utilisés, les aides non chirurgicales mises en œuvre et listé les projets non aboutis.

En 8 ans nous estimons avoir opéré 241 patientes majoritairement agricultrice ou femme au foyer et ne parlant peu ou pas le Français (langue officielle) n'ayant pas été scolarisées au-delà du primaire. Il s'agissait exclusivement de FO après AVB ou césarienne de type complexe selon l'OMS dans plus de la 2/3 des cas. Plus de 65% des réparations étaient des voies basses. Le taux global de fermeture de la fistule était de 69% (89% des simples 56% des complexes). Cependant le suivi des résultats était complexe avec un fort de taux de perdues de vues après l'ablation de la sonde (incertitude quant aux résultats tardifs). Les financements se faisaient essentiellement par des dons et des événements caritatifs (concerts, ventes aux en-chères). Une subvention d'une fondation nord-américaine a été obtenue. Trois infirmiers ont été financés pour des formations complémentaires dont un directement en lien avec la prise en charge des FVV. Nous avons accompagné et soutenu un jeune chirurgien académique devenu Professeur d'urologie à Ouagadougou et continuant la chirurgie des FVV. Ce chirurgien fait partie aujourd'hui de notre groupe de chirurgien expert et se déplace sur d'autres sites (Rwanda par exemple). Nous n'avons pas réussi à monter un centre pérenne de traitement des fistules avec une maison de réinsertion comme nous l'avions projeté. Nous n'avons pas réussi à établir de lien pérenne et porteur avec les autorités de santé Burkinabé. Notre action est suspendue depuis l'instabilité politique et sécuritaire advenue depuis la chute du Président Campaoré et les premiers attentats de Ouagadougou auxquels nos équipes ont été confrontées.

Une action solidaire et collaborative médico-chirurgicale peut aider à améliorer la prise en charge des FO dans un pays à faible revenu. Les blocages institutionnels sont fréquents, les enjeux locaux-régionaux autour des FO peuvent rendre inopérant voire contre-productif un partenariat basé exclusivement sur du soin chirurgical.

# Research article: La diphtérie: une maladie qui sévit encore à Mayotte en 2021

**Published:** 2021-11-30

**Authors:** Marion Subiros, A. Ahmed, M. Jean, P. Durasnel, L. Fonteneau, T. Benoit-Cattin, Edgar Badell

**Affiliations:** Santé publique France, Centre Hospitalier de Mayotte, Rue de l'hôpital, Mamoudzou 97600, Mayotte, France

**Keywords:** Diphtérie, Épidémiologie, Risque réémergence, Mayotte, Océan Indien

Grâce à une couverture vaccinale (CV) très élevée, la diphtérie due à *C. diphtheriae* a disparu en France métropolitaine. La quasi-totalité des cas déclarés depuis 20 ans sur l'ensemble du territoire sont des cas importés de pays où sévissent des épidémies. Depuis une dizaine d'année, la moitié des cas de diphtérie à *C. diphtheriae* déclarés sont localisés à Mayotte.

La surveillance épidémiologique de la diphtérie à Mayotte repose sur le dispositif de surveillance des maladies à déclaration obligatoire. Les signalements sont effectués par les cliniciens et biologistes de l’île, les cas déclarés sont les cas dus à des souches porteuses du gène tox. Des investigations sont menées systématiquement autour de chaque cas confirmé afin de détecter l'origine de la contamination, d'identifier l'ensemble des contacts et de mettre en œuvre les mesures de gestion (vérification des statuts vaccinaux, traitements prophylactiques). Le contexte local rend complexe le suivi des patients, souvent perdus de vue avant enquête. Depuis 2012, en lien avec l'identification régulière de cas sur le territoire, le laboratoire du CH de Mayotte dispose d'une procédure de diagnostic rapide de portage de *C. diphtheriae* et mise en évidence du gène tox.

Habituellement, 0 à 2 cas dus à des isolats porteurs du gène tox (tox+) sont signalés chaque an-née à Mayotte. Entre 2012 et 2019, la majorité des 16 cas confirmés à Mayotte était des cas de diphtérie cutanée dont la moitié importée des Comores.

La situation a évolué au cours de l'année 2019 avec six cas de diphtérie signalés dont cinq formes cutanées. Il s'agissait de quatre enfants (6 mois à 4 ans) et une femme de 40 ans. Un des cas était importé d'Anjouan (Comores). Les investigations n'ont pu être réalisées qu'auprès de trois cas, révélant chaque fois, une dizaine de contacts et des statuts vaccinaux incertains. Aussi, un cas de diphtérie ORL a été déclaré chez un enseignant qui n'avait jamais bénéficié d'une vaccination. Pour ce cas, 175 sujets contacts ont été identifiés. L'enquête a montré une CV insuffisante dans la population des contacts, notamment chez les collègues du cas.

Les données de l'année 2020 ont continué de montrer une situation préoccupante. Quatre cas de diphtérie cutanée tox+ ont été diagnostiqués chez trois adultes de 45, 58 et 63 ans dont les statuts vaccinaux étaient inconnus et chez un enfant de 11 ans a priori à jour de ses vaccinations.

Enfin, au cours du premier semestre 2021, deux nouveaux cas ont été déclarés: un cas de diphtérie cutanée tox+ chez un enfant de 7 ans, a priori à jour de ses vaccinations et un cas de diphtérie ORL tox+, survenu en avril 2021 chez un enfant de 4 mois, n'ayant reçu aucun vaccin du nourrisson. L'enfant est décédé d'un choc toxinique.

À Mayotte, les données de CV sont anciennes (InVS 2010). En 2021, sortiront les résultats de l'enquête de CV menée en population générale en 2019. En attendant, quelques études ciblées sur des populations spécifiques (enfants de moins de 6 ans, professionnels de santé) ont mis en évidence des niveaux de CV largement insuffisants pour assurer une immunité collective protectrice dans la population. Cette situation expose le territoire à l'apparition de nouveaux cas de diphtérie ainsi que d'autres maladies à prévention vaccinale telles que la coqueluche ou la rougeole. Ce risque est d'autant plus à craindre que les pays de la zone d’échange de Mayotte montrent une incidence non négligeable de ces maladies potentiellement mortelles.

Ces données confirment la place de la vaccination au cœur des enjeux de santé publique à Mayotte en 2021. Les interventions destinées à élever la CV de la population de Mayotte doivent être renforcées avec une attention portée aux populations vulnérables et/ou éloignées du recours aux soins.

# Research article: Prévention de l'infection par la Covid-19 chez les personnes atteintes de diabète au Mali et au Burkina Faso

**Published:** 2021-11-30

**Authors:** Stéphane Besançon

**Affiliations:** Santé diabète, 1 Avenue Malherbe, 38100 Grenoble, France

**Keywords:** Covid-19, Diabète, Comorbidités, Mali, Burkina Faso, Afrique subsaharienne

Les informations scientifiques disponibles le montrent: le diabète n'augmente pas les risques d'infection par la Covid-19. En revanche, elles montrent que les personnes atteintes de diabète dont la prise en charge de la maladie n'est pas optimale sont à risque de développer des formes plus graves de la Covid-19. En Afrique, cette inquiétude est renforcée pour les personnes atteintes de diabète car elles présentent des diabètes souvent moins bien équilibrés, ainsi que d'autres comorbidités associées comme l'obésité ou encore l'hypertension artérielle. Dans le but de prévenir l'infection par la Covid-19 et les complications liées au diabète, Santé diabète a mis en place au Burkina Faso et au Mali un programme spécifique de prévention Covid-19/diabète et de suivi des patients. Ce programme s'est décliné en trois axes stratégiques:

1. Mise en place de campagnes de prévention à destination des personnes « à risque ».
  Santé diabète a mis en place au Burkina Faso et au Mali un programme de suivi spécifique des personnes atteintes de diabète. Ce programme de prévention, entièrement gratuit pour les personnes, s'articule autour de plusieurs axes: l'accès à l'information pour plus de 2 500 personnes atteintes de diabète, à travers une campagne de SMS ciblant spécifiquement ces patients. En parallèle, une campagne de prévention dans les médias a eu pour but de toucher un plus grand nombre de patients.
2. Mise en place de téléconsultation à destination des personnes « à risque ».
  Ensuite, un programme de « téléconsultation » a été mis à disposition des personnes atteintes de diabète qui le souhaitent à travers des plateformes d’échanges whatsapp avec des médecins spécialistes diabétologues ou endocrinologues. Le but était de réaliser des téléconsultations permettant d'accentuer la prévention, de mieux suivre l’évolution de la maladie chronique, tout en évitant de se déplacer dans les structures de santé si le besoin n'est pas urgent.
3. Dotation des patients et sécurisation de l'approvisionnement en insuline pour éviter toute rupture.
  En parallèle, Santé diabète a mis en place, dans le cadre d'un partenariat avec Médecins Sans Frontières (MSF), des dons de masques pour tous les patients atteints de diabète. Enfin, main dans la main avec les autorités sanitaires burkinabés et maliennes, Santé diabète a travaillé à s'assurer que l'approvisionnement en insuline était sécurisé au moment où la majorité des pays du monde connaissait un lockdown.

# Research article: Complications chroniques du diabète de type 1 chez l'enfant à Dakar

**Published:** 2021-11-30

**Authors:** Modou Gueye, Amadou Sow, Djibril Boiro

**Keywords:** Diabète type 1, Enfant, Complications chroniques, Dakar, Sénégal, Afrique subsaharienne

Les complications chroniques du diabète de type 1 sont rares chez l'enfant. L'objectif de ce travail est de déterminer leur prévalence en milieu pédiatrique et les facteurs qui leurs sont associés.

Il s'agissait d'une étude prospective, menée dans deux services de référence dans la prise en charge du diabète de l'enfant au CHU de Dakar. Nous avons inclus tous les patients diabétiques de type 1 et dont l’âge était inférieur à 20 ans selon les recommandations de l'ISPAD 2009.

Sur les 67 patients de notre cohorte, 46 seulement répondaient aux critères d'inclusion. Il y avait 19 garçons et 27 des filles. L’âge moyen était de 11,6 ans +/- 4,3 ans L’âge moyen de découverte était de 8,7 ans+/-3,8 ans. La durée moyenne d’évolution était de 34 mois. Près de la moitié des patients avaient une hémoglobine glyquée moyenne supérieure à 9%. Le retard de crois-sance était sévère dans 17% et modéré dans 11% des cas. Cinq patients (20%) ont présenté une microalbuminurie significative et 5 (20%) avaient une rétinopathie dont 3 avec un œdème maculaire et les 2 autres avec des microhémorragies diffuses.

L'analyse multivariée des différents paramètres étudiés montrait que la rétinopathie était associée aux valeurs élevées de l'HbA1c (p=0,043) et à la mauvaise observance thérapeutique (p=0,021). Quant à la néphropathie, elle était seulement associée à la mauvaise observance thérapeutique avec p=0,0025.

Les complications chroniques du DT1 ne sont pas rares surtout dans nos régions où la prise en charge n'est pas optimale. Nous suggérons de les dépister plutôt vers 10-11 ans quelle que soit la durée d’évolution du diabète.

# Research article: De la nécessité d'aborder les inégalités de santé pédiatriques en Guyane

**Published:** 2021-11-30

**Authors:** Lindsay Osei, Célia Basurko, Mathieu Nacher, Nicolas Vignier, Narcisse Elenga

**Affiliations:** Centre hospitalier de Cayenne, Avenue des flamboyants, Cayenne 97306, Guyane française, France

**Keywords:** Pédiatrie, Inégalités sociales de santé, Guyane française

La Guyane est une région française d'Outre-Mer située en Amérique du Sud, frontalière du Brésil et du Suriname, et qui fait face à des inégalités socio-économiques marquées. Plusieurs études ont montré que les adultes les plus précaires de ce territoire étaient plus sujets aux maladies, tant aiguës que chroniques. Les femmes enceintes sont également plus susceptibles d’être sujettes à des complications obstétricales et les naissances prématurées sont une préoccupation majeure dans cette région. Si pour les adultes, les femmes enceintes et les nouveau-nés, l'impact des inégalités sociales de santé sont identifiées, les conséquences de ces inégalités sociales de santé chez les enfants et les adolescents vivant en Guyane ont été peu décrites. De plus, les crises économiques successives accentuent cette situation déjà préoccupante, notamment avec la pandémie de Covid-19.

Un état des lieux des données disponibles a été réalisé pour mieux appréhender la question des inégalités sociales pédiatriques en Guyane.

Une revue narrative des données disponibles a été effectuée en utilisant les moteurs de recherches traditionnels et en sollicitant les acteurs de référence de Guyane.

Certains indicateurs de santé pédiatriques sont moins bons que ceux retrouvés en France hexagonale. Cette région concentre un fort taux de malformations congénitales et anomalies chromosomiques avec pour conséquence un taux de mortalité infantile qui est le triple de ce qui est rapporté en France hexagonale. Les maladies infectieuses sont également d'intérêt notable, avec une couverture vaccinale insuffisante pour ce territoire; de plus celles-ci représentent la première cause de décès des enfants de moins de 15 ans, avec notamment des méningites dues à des agents pathogènes pour lesquels des vaccins existent et sont recommandés dans le calendrier vaccinal français. L'accès à l'eau et à l'alimentation est problématique pour une partie de la population avec des épidémies rapportées dans certaines zones avec un accès à l'eau potable difficile. Des cas de saturnisme sont également rapportés, principalement dans des zones géographiques précaires.

Cette présentation se propose d'aborder ce sujet émergent des inégalités sociales de santé en Guyane et de présenter des axes d'amélioration de cette situation.

# Research article: Deux technologies simples et faciles à mettre en œuvre pour réaliser des formes pédiatriques de médicaments à partir de comprimés ou de gélules aux dosages adultes.

**Published:** 2021-11-30

**Authors:** Thierry Breul, Laurène Blanc, Nadia Kasmi

**Affiliations:** Institut Nord – Sud de Coopération Biopharmaceutique (INSCB) Residence Les Marines, Apt 146, Impasse des plaisanciers, 34110 Frontignan, France

**Keywords:** Formes pédiatriques, Formulation galénique, Médecine tropicale

Ce travail présente deux technologies simples et faciles à mettre en œuvre pour réaliser des formes galéniques de médicaments à des dosages pédiatriques à partir de comprimés broyés ou de gélules aux dosages adultes. Ces technologies sont particulièrement adaptées aux conditions tropicales où les formes pédiatriques sont parfois manquantes.

La première technologie présentée est une technologie d'injection moulage. Elle consiste au broyage de comprimés aux dosages adulte, puis au mélange et à l'homogénéisation du broyat obtenu à chaud avec une solution à base de sorbitol fondu dans une seringue graduée. Après refroidissement du mélange obtenu dans la seringue, on procède à l'injection moulage du mélange dans des moules en silicone. En fonction des dosages désirés on peut par exemple remplir 16 moules qui chacun contiennent 1/16 de la dose adulte. Après refroidissement des moules, la solution du mélange injectée et moulée forme des pastilles d'environ un gramme, qui sont soit directement administrables à l'enfant, soit à dissoudre dans un verre d'eau.

La deuxième technologie est une technologie d'extrusion à froid. Elle consiste aussi à mélanger les comprimés broyés ou le contenu des gélules aux dosages adulte avec une suspension de farine et d'eau, à homogénéiser le mélange à chaud dans une seringue, à laisser refroidir le mélange, puis à placer la seringue dans un pousse seringue automatique de façon à extruder le mélange pour obtenir un jonc (ou spaghetti) régulier.

À partir du jonc (ou spaghetti) obtenu, on peut le couper à une longueur prédéfinie de façon à obtenir une forme galénique au dosage désiré. Le jonc découpé peut-être administré directement à l'enfant.

Ces deux technologies d'injection moulage et d'extrusion à froid sont simples et rapides à mettre en œuvre, elles ne nécessitent pas de matériels sophistiqués ou onéreux, et s'adaptent à tous types de médicaments. Elles permettent d'obtenir des formes galéniques pédiatriques à des dosages très précis qui sont des fractions des dosages des médicaments adultes.

Les formes obtenues sont stables et directement administrables aux enfants.

# Research article: La fabrique d'un label autour d'une dénomination: phyto-médicament vs médicament traditionnel amélioré (MTA)

**Published:** 2021-11-30

**Authors:** Adèle Poukom Yameogo, Eugénie D'Alessandro, André Kamba Soubeiga

**Affiliations:** Département de sociologie, Université Joseph Ki-Zerbo, 03 BP 7021 Ouagadougou, Burkina Faso

**Keywords:** Phyto-médicaments, MTA, Médecine traditionnelle, Burkina Faso, Afrique subsaharienne

En prenant pour objet d’étude une enquête sociologique sur les médicaments issus de la médecine traditionnelle africaine, cette communication questionne les enjeux qui émergent autour de la fabrique d'un label pour la dénomination « phyto-médicament » contrairement à celle de « médicament traditionnel amélioré » (MTA) par des « hommes de sciences » - producteurs de médicaments à base de plantes au Burkina Faso. La préférence pour l'appellation « phyto-médicamen » par ces derniers décline leurs perceptions ainsi que les contours qu'ils délimitent pour chaque appellation. Dans certains référentiels thérapeutiques comme la liste nationale des médicaments essentiels et autres produits de santé, c'est le vocable « médicament traditionnel amélioré » qui est usité. Pourquoi cette qualification apparaît comme problématique pour certains acteurs? « Médicament traditionnel amélioré » est un nom générique introduit par l'OMS, qui, aujourd'hui, l'a substitué à médicament issu de la médecine et pharmacopée traditionnelles. À partir du sens attribué à un nom de médicament, c'est tout un corpus de pratiques d'acteurs - producteurs et prescripteurs - qui peuvent émerger. La dénomination, en elle-même renvoie à une dimension certes abstraite mais aussi symbolique. L'entrée par la dénomination réintroduit à nouveau la question de la légitimité de la médecine traditionnelle. Dans le contexte africain, les médicaments issus de la médecine traditionnelle, même après avoir subi des transformations et acquis une autorisation de mise sur le marché, gardent l’étiquette « traditionnel » dans leur caractérisation. Ce constat renvoie à l'image toujours véhiculée sur cette médecine, décrite comme une médecine empirique. Contrairement à la médecine chinoise ou la médecine ayurvédique, qui, elles sont présentées comme des médecines savantes; on fait moins référence au terme « traditionnel » pour caractériser la médecine et les médicaments issus de ces contrées. Cette communication propose d'analyser comment des logiques d'acteurs autour de la dénomination d'une catégorie de médicaments structurent des pratiques et mettent en lumière plusieurs enjeux.

# Research article: Vers l’élimination du paludisme à Mayotte?

**Published:** 2021-11-30

**Authors:** Jean-François Lepère

**Affiliations:** Centre Hospitalier de Mayotte, Rue de l'hôpital, Mamoudzou 97600, Mayotte, France

**Keywords:** Paludisme, Élimination, Mayotte, Océan Indien

Mayotte, département français de l'Océan Indien, est l'une des quatre îles de l'archipel des Comores, zone de transmission permanente du paludisme, quasi exclusivement à *Plasmodium falciparum.*

Confrontées à une augmentation régulière de la morbidité et de la mortalité palustre les autorités sanitaires de l’île ont mis en place depuis fin 2001 plusieurs mesures successives de renforce-ment de la lutte antipaludique. Les moyens diagnostiques et thérapeutiques ont été améliorés selon les préconisations de l'OMS, la surveillance épidémiologique mise en place et la lutte anti-vectorielle renforcée. Ce programme de lutte contre le paludisme est-il efficace?

De 2002 à 2020, 4819 cas acquis localement ont été rapportés à Mayotte où l'incidence annuelle a diminué de 10,3‰ en 2002 (1649 cas) à moins de 0,01‰ en 2020 (2 cas). Durant cette période, 1879 cas importés ont été observés. Ils proviennent principalement de l'Union des Comores (85,8%), de Madagascar (8,6%) et d'Afrique subsaharienne (5,6%).

Depuis 2010, l'incidence des cas acquis localement est inférieure à 1 ‰. En 2014 l'OMS classe Mayotte parmi les territoires en phase d’élimination du paludisme. À partir de 2017, le nombre annuel de cas acquis localement est inférieur à 10 et décroît régulièrement (9 cas en 2017, 5 en 2018, 4 en 2019 et 2 en 2020).

La répartition temporo-spatiale de ces rares cas acquis localement suggère qu'il s'agit de cas introduits et non de cas indigènes. L’étude du profil génotypique des souches plasmodiales des cas localement acquis observés en 2017 et 2018 confirme qu'il s'agit certainement de cas introduits en lien avec des cas importés de l'Union des Comores voisine.

Le dernier cas acquis localement a été notifié à Mayotte en juillet 2020. Eu égard aux caractéristiques du cycle des plasmodies, à l'absence d'immunité palustre de la population mahoraise et à la longévité des vecteurs anophéliens, il est légitime à ce jour de penser que la transmission indigène du paludisme est interrompue à Mayotte.

L'arrêt de la transmission indigène du paludisme semble être un fait acquis à Mayotte, mais l’île reste sous la menace d'une réintroduction via des cas importés des pays voisins. Il est temps d’élaborer un plan local de prévention de la réintroduction et de mettre en œuvre une politique volontariste de coopération régionale en matière de lutte contre le paludisme.

# Research article: Analyse des données de surveillance épidémiologique du paludisme au Mali (2017-2019)

**Published:** 2021-11-30

**Authors:** Ousmane Boua Togola, Yacouba Kone, Pauline Kiswensida Yanogo, Djibril Barry, Mariame Laurent, Ken Kayembe, Bouyagui Traore, Hanine Keita, Jean Kabore, Ibrahima Berthe, Fadima Diallo, Nicolas Meda

**Affiliations:** Direction générale de la santé et de l'hygiène publique, BP 232 Koulouba, Bamako, Mali

**Keywords:** Paludisme, Surveillance, Morbidité, Mortalité, Mali, Afrique subsaharienne

En 2018, le paludisme de la femme enceinte a entraîné 900 000 cas d'insuffisance pondérale dans 38 pays d'Afrique subsaharienne. Cinq pays d'Afrique francophone dont le Mali, supportent le quart de la charge mondiale du paludisme où un enfant y meurt toutes les deux minutes. Au Mali, le paludisme constitue la première cause de mortalité des enfants de moins de cinq ans. L'objectif de notre étude était d'analyser les données de surveillance du paludisme pour décrire les caractéristiques en temps lieu et personne, la qualité des données et les moyens diagnostics.

Nous avons conduit une étude transversale descriptive du 1^er^ septembre au 27 décembre 2020 sur les données de surveillance du paludisme de 2017 à 2019. Nous avons inclus tous les cas enregistrés dans le District Health Information Software version 2 (DHIS2) et calculé les proportions et les ratios à l'aide d'Epi-Info 7.2.

Une discordance entre les cas traités et les cas confirmés biologiquement de paludisme était marquée à Sikasso et Mopti. Le ratio femme/homme était de 1,03. La prévalence nationale du paludisme était respectivement de 9,53; 8,96 et 10,05 en 2017, 2018, 2019. Le test de diagnostic rapide était le plus utilisé avec 80,31%, 64,97%, 79,48% respectivement en 2017, 2018, 2019. Les faibles taux de confirmation du paludisme étaient enregistrés en 2017 à Taoudénit (32%), en 2018 à Koulikoro et Ménaka avec 36% et, en 2019 à Kidal et Taoudénit avec 32%. La morbidité est passée de 36,23% à 31,95% et la mortalité de 78,35% à 71,18% de 2017 à 2019 chez les enfants de moins de 5 ans. Les fortes létalités étaient observées à Sikasso (7 ‰), Mopti (7 ‰) et Tombouctou (9 ‰) respectivement en 2017, 2018, 2019.

Notre étude a relevé une stabilité de la prévalence du paludisme, un usage inapproprié des moyens diagnostics et des médicaments antipaludiques. Nous recommandons une supervision formative des agents de santé sur les normes et procédures de la gestion des intrants antipaludiques à tous les niveaux de la pyramide sanitaire.

# Research article: *Plasmodium ovale wallikeri* et *Plasmodium ovale curtisi*: du développement d'une méthode de différenciation à l'analyse rétrospective des cas du Centre national de référence du paludisme, période 2013-2018

**Published:** 2021-11-30

**Authors:** Valentin Joste, Justine Bailly, Véronique Hubert, Cécile Pauc, M. Gendrot, Emilie Guillochon, M. Madamet, M. Thellier, E. Kendjo, N. Argy, Bruno Pradines, S. Houzé

**Affiliations:** Centre national de référence du Paludisme, Unité Parasitologie et entomologie, Institut de recherche biomédicale des armées, IHU Méditerranée Infection, 19-21 Bd Jean Moulin, 13005 Marseille, France

**Keywords:** Paludisme, *Plasmodium ovale wallikeri*, *Plasmodium ovale curtisi*, qPCR-HRM, Test de diagnostic rapide

*Plasmodium ovale* spp est une des 5 espèces de *Plasmodium* pouvant infecter l'homme. Une de ses caractéristiques notables est sa capacité à donner des accès de reviviscence, qui sont définis par la réapparition de formes asexuées de *Plasmodium ovale* spp dans le sang périphérique après un traitement antipaludique adapté et bien suivi, sans nouvelle contamination. Depuis 2010, cette espèce a été séparée en *Plasmodium ovale curtisi* et *Plasmodium ovale wallikeri* sur la base de séquences génétiques distinctes.

Nous avons développé une méthode de qPCR-HRM permettant de les distinguer. Grâce à cette méthode, nous avons identifié 368 isolats de *Plasmodium ovale wallikeriet* 309 isolats de *Plasmodium ovale curtisi* reçus au CNR du Paludisme entre janvier 2013 et décembre 2018. Les données épidémiologiques, cliniques et biologiques recueillies ont permis de mettre en évidence une thrombopénie plus sévère (94 G/L [70-130] vs 111 G/L [84-145], p<0,001) et un délai de latence plus court (34 jours [10-95] vs 72 jours [18-208], p<0,001) lors des infections à *Plasmodium ovale wallikeri.* De plus, les patients infectés par *Plasmodium ovale wallikeri* ont été plus souvent traités par une combinaison thérapeutique à base d'artémisinine (29,2% vs 17,1%, p<0,001). Bien que non statistiquement significatif, les patients infectés par *Plasmodium ovale wallikeri* ont une tendance à être hospitalisés plus fréquemment en unités de soins intensifs/unités de soins continus (p=0,134) et à présenter une thrombopénie sévère (p=0,123) que les patients infectés par *Plasmodium ovale wallikeri.*

Concernant les méthodes diagnostiques, les techniques immunochromatographiques détectant l'aldolase sont plus sensibles que celles détectant la pLDH (47,8% vs 10,6%, p<0,001).

Enfin, nous avons analysé les séquences du gène potra chez 90 isolats de *Plasmodium ovale* spp et mis en évidence que ce gène n’était pas suffisamment polymorphique pour être utilisé dans un objectif de typage génétique des reviviscences qui sont définies de façon clinique et épidémiologique.

# Research article: Résistance aux pyréthrinoïdes et au DDT chez la population d'Anopheles arabiensis de Marovoay, Madagascar

**Published:** 2021-11-30

**Authors:** Tsiriniaina Rakotondranaivo, Ousmane Ndiath, Milijaona Randrianarivelojosia

**Affiliations:** Institut Pasteur de Madagascar, BP 1274 Ambatofotsikely Avaradoha, 101 Antananarivo, Madagascar

**Keywords:** *Anophele*, Lutte antivectorielle, Résistance, Marovoay, Madagascar, Océan Indien

À Madagascar, la lutte contre le paludisme est essentiellement basée sur la prise en charge des cas, les campagnes de pulvérisation intra-domiciliaires d'insecticides et les campagnes de distribution de moustiquaires imprégnées d'insecticides. Il est impératif de surveiller la sensibilité des anophèles aux insecticides. Dans cette optique, nous avons évalué la sensibilité aux différents insecticides des populations *d'Anopheles arabiensis* dans le district de santé de Marovoay en mars 2017. Des larves et nymphes d'anophèles collectées dans des gîtes à Tsararano ont été élevées pendant 10 à 12 jours. Après une identification morphologique, 650 anophèles femelles adultes de deux à cinq jours, appartenant au complexe *Anopheles gambiae,* ont été utilisées pour les tests de sensibilité à la lamda-cyhalothrine (0,05%), à la deltaméthrine (0,05%), au dichlorodiphényltrichloroéthane ou DDT (4%), au malathion (5%), au fénitrothion (1%) et au bendiocarbe (0,1%) selon le protocole recommandé par l'OMS. Des tests PCR ont été effectués pour identifier les membres du complexe *An. gambiae* parmi ces anophèles testés. Les individus testés ont été de l*‘An. arabiensis* selon les résultats des PCR. La population d*‘An. arabiensis* de Tsararano a été résistante aux lamda-cyhalothrine et Deltaméthrine (pyréthrinoïdes) et au DDT, avec des taux de mortalité respectifs de 51% (IC95%: 43-57,7%), 49% (CI95%: 44,1-57%) et 42% (CI95%: 37,5-45,3%). Par contre, elle a été sensible (100%) au carbamate (bendiocarbe) et aux organophosphates (malathion et fénitrothion). Nos résultats démontrent ainsi la résistance d*‘Anopheles arabiensis* à Tsararano, Marovoay. Cette situation est alarmante car les pyréthrinoïdes sont recommandés dans l'imprégnation des moustiquaires. C'est une menace potentielle au programme de lutte antivectorielle dans cette région. Aussi, comme nos données ont montré la résistance d*‘An. arabiensis* au DDT et aux pyréthrinoïdes, l’éventualité d'une résistance de type *knockdown resistance* (kdr) qui n'a jamais été observé à Madagascar jusqu’à présent sera abordée lors de notre présentation. Nous avons généré des données utiles et utilisables pour améliorer la politique de lutte contre le paludisme à Madagascar où le recours aux moustiquaires de troisième génération (pyréthrinoïdes + synergistes) pour lutter contre les vecteurs résistants aux pyréthrinoïdes est à prendre en compte.

# Research article: Revue de résistance multiple aux insecticides chez *Anopheles gambiae* s.l et défis liés à la lutte antivectorielle en Afrique de l'Ouest

**Published:** 2021-11-30

**Authors:** Abdoul-Aziz Maiga, Athanase Badolo

**Affiliations:** Laboratoire d'entomologie fondamentale et appliquée, Université Joseph Ki-Zerbo, 03 BP 7021 Ouagadougou, Burkina Faso

**Keywords:** Paludisme, Résistance multiple, *Anopheles gambiae*, Afrique de l'Ouest, Afrique subsaharienne

Le paludisme reste la maladie la plus mortelle en Afrique subsaharienne et en Afrique de l'Ouest en particulier. En absence d'un vaccin efficace, la lutte antivectorielle (utilisation de moustiquaires imprégnées d'insecticide de longue durée d'action-MILDA- et Pulvérisation intra-domiciliaire-PID) reste fondamentale dans le contrôle de la maladie. Cependant, l’émergence de résistance multiple aux insecticides chez *Anopheles gambiae* s.l, principal vecteur dans la région, peut réduire considérablement l'efficacité des interventions de lutte. Ainsi, l'objectif de cette revue est de i) Faire un état de lieu sur le niveau d'expansion de cette résistance en région Ouest Africaine ii) Révéler le défi lié à la lutte antivectorielle iii) Discuter l'implémentation de stratégies et méthodes alternatives innovantes.

Les bases de données bibliographiques en ligne Pubmed, Google Scholar ont été utilisées en insérant des mots clés « Malaria », « insecticide resistance », « *Anopheles gambiae* » et « Africa ». La période concernée était de 2000 à 2018. Les informations comme: année & sites d’études, espèces de moustiques, insecticides utilisés, types de résistance et mécanismes impliqués ont été extraits et filtrés. Le critère d'inclusion était: présence de résistance phénotypique associée à la fois à la mutation de cible et à l'implication des enzymes métaboliques.

Au total 20 articles ont été inclus. Les résultats révèlent une large expansion de cette résistance multiple chez le vecteur du paludisme *An. gambiae* s.l dans les différentes zones écologiques (Forestières et Savanicoles) et même en zone urbaine de la région de l'Afrique de l'Ouest. Peu de données sont disponibles sur *An. melas* en zone mangrove.

L'expansion de cette résistance multiple chez *An. gambiae* s.l compromet l'effort de lutte contre le paludisme et son élimination. Le gap révélé est l'absence d'harmonisation des stratégies de lutte antivectorielle en zones transfrontalières inter pays et le peu d'intérêt porté sur le statut de résistance et mécanismes impliqués chez *An. melas*, principal vecteur du paludisme en zone mangrove. Il est urgent de rendre applicable les directives sur la gestion de résistance au sein des programmes nationaux de lutte afin de maintenir l'efficacité des interventions de lutte. Des méthodes alternatives basées sur le contrôle biologique et génétique: infection au *Wolbachia* spp, lâcher de mâles stériles et moustiques transgéniques doivent être envisagées. Les communautés doivent être sensibilisées afin de s'approprier la gestion de l'environnement.

# Research article: Améliorer l'accès aux services de santé sexuelle et reproductive des personnes en situation de handicap au Sénégal et en Sierra Leone

**Published:** 2021-11-30

**Authors:** C. Guinard, B. Thurston, E. Duffy, S. Palmer

**Affiliations:** ThinkPlace Sénégal, Lot 10, Route Ngor a Almadies, Les Almadies, Dakar, DA 10200

**Keywords:** Santé sexuelle et reproductive, Handicap, *Human centred design*, Approche qualitative, Inclusivité, Sénégal, Sierra Leone, Afrique subsaharienne

Le programme WISH (*Women's Integrated Sexual Health*) est un programme visant à renforcer les services intégrés de santé et de droits sexuels et reproductifs (SDSR) dans plusieurs pays d'Afrique et d'Asie d'ici 2021. En tant que partenaire expert en conception centrée sur l'humain (*Human Centred Design*) et en partenariat avec Leonard Cheshire Disability et Marie Stopes International (MSI), ThinkPlace a mené un processus de co-création en Sierra Leone et au Sénégal afin développer des interventions qui s'attaquent à ces obstacles et augmentent l'inclusion dans les services de SSR des personnes vivant avec un handicap.

En Afrique de l'Ouest, les personnes handicapées font face à des barrières à la fois structurelles, sociales, culturelles et économiques pour accéder aux services de SSR.

En raison des restrictions dues à la Covid-19, ThinkPlace a utilisé une approche de «Centre-Périphérie» pour s'assurer de créer des interventions rigoureusement conçues et testées par les designers expérimentés de ThinkPlace, puis localement adaptées à d'autres contextes où nous n'avons pas pu nous rendre. Dans ce projet, les prototypes (potentielles solutions à tester) ont été développés et testés au Sénégal, puis, pour l'adaptation en Sierra Leone, les aspects centraux ont été reproduits à l'identique tandis que les éléments périphériques plus malléables ont été adaptés aux besoins et contextes des communautés locales.

Pendant deux semaines, ThinkPlace a travaillé avec une organisation de personnes handicapées (OPH) et des équipes mobiles locales au Sénégal pour tester et itérer cinq interventions visant à améliorer l'inclusivité des services de SSR pour les personnes ayant des handicaps auditifs, visuels, physiques et psychologiques. Une fois la première série de tests menée au Sénégal, un ensemble de trois prototypes de fidélité moyenne ont été transférés en Sierra Leone pour être testés et affinés.

Grâce au renforcement des capacités et au mentorat à distance de ThinkPlace, l’équipe locale de MSI en Sierra Leone a effectué trois semaines de tests, qui ont abouti à trois prototypes localement adaptés.

ThinkPlace a ensuite développé des guides simples et facile d'utilisation pour la mise en œuvre, la diffusion et la mise à l’échelle de ces outils parmi les partenaires du consortium WISH dans les pays concernés par le projet, mais aussi dans les autres pays d'Afrique dans lequel WISH intervient (RDC, Niger, Nigeria, entre autres).

# Research article: Analyse des données de la prévention de la transmission mère-enfant du VIH (Mali, 2010-2019)

**Published:** 2021-11-30

**Authors:** M.S. Diarra, Y. Ballayira, P.K. Yanogo

**Affiliations:** Burkina Field Epidemiology and Laboratory Training Program (BFELTP), Ouagadougou, Burkina Faso

**Keywords:** Analyse, Prévention transmission mère-enfant, VIH/sida, Mali, Afrique subsaharienne

Dans le monde, le nombre de femmes enceintes vivant avec le VIH était de 1,4 millions dont 93% en Afrique subsaharienne selon l'ONUSIDA en 2016. Les moins de 15 ans vivant avec le VIH représentaient 1,8 millions dans le monde avec 89% en Afrique subsaharienne. Au Mali, le sexe ratio est de 1,63 femmes pour un homme pour le VIH/sida. L'objectif de cette étude était d'analyser les données de prévention pour la transmission mère-enfant du VIH au Mali du 1^er^ janvier 2010 au 31 décembre 2019.

Nous avons conduit une étude transversale de février à mars 2021 sur les données de PTME 2014 à 2018. Nous avons effectué une revue documentaire et un formulaire d'extraction des données des rapports mensuels. Nous avons analysé les données en temps, lieu et personnes en utilisant Excel 2016. Nous avons exprimé nos résultats en proportions et nombres dans des tableaux.

Le taux de complétude était à 91%. Les femmes enceintes de 25-34 ans étaient les plus représentées avec 48,6% parmi 1 715 625 femmes enceintes vues en consultations prénatales. Le district de Bamako a dépisté le plus grand nombre de femmes enceintes soit 38,6%. Le taux moyen d'enfants avec *Polymerase Chain Reaction* positive était de 4,4% et confirmés à 18 mois de 11,2%. La proportion d'enfants nés de mères séropositives ayant bénéficié de la prophylaxie antirétrovirale était de 91,0% par région en moyenne. Le taux d'allaitement maternel chez les nouveau-nés était de 88,5% en moyenne. Le taux moyen de dépistage du VIH était de 74,4%. Le taux de séropositivité était de 0,8%.

Notre étude a permis de détecter qu'un des problèmes majeurs est la complétude des données (canevas non identiques 2010-2013 et 2014-2018) et la qualité de la prévention de la transmission mère-enfant au Mali. La promptitude des données n'a pas pu être appréciée car non disponible dans les rapports. Cette analyse a permis d'avoir un taux d'enfants séronégatifs à 84,4% d'où la nécessité d'augmenter ces sites de prise en charge.

# Research article: Évaluation du système de surveillance des lésions précancéreuses et cancéreuses du col de l'utérus au Niger de 2017 à 2019

**Published:** 2021-11-30

**Authors:** M. Mahamadou Yacouba, A. Ibrahim, S. Tiendrebeogo, P. Yanogo, J. Kabore, A. Yacouba, M. Meda

**Affiliations:** Burkina Field Epidemiology and Laboratory Training Program (BFELTP), Ouagadougou, Burkina Faso

**Keywords:** Col de l'utérus, Lésion précancéreuse, Cancer, Surveillance, Niger, Afrique subsaharienne

Le cancer en général et celui du col de l'utérus en particulier constituent une cause importante de morbidité et de mortalité parmi les maladies non transmissibles dans le monde. Le cancer du col de l'utérus est une tumeur maligne d'origine épithéliale qui se forme dans les tissus du col utérin. Il survient le plus souvent chez les femmes de 15 ans et plus. En Afrique de l'Ouest, environ 4,3% des femmes sont estimées à risque de développer ce cancer. En 2018 au Niger, les estimations ont indiqué que 543 femmes ont été diagnostiquées du cancer du col de l'utérus dont 476 décès. D'où le choix de cette pathologie pour évaluer son système de surveillance afin de contribuer à améliorer le dépistage des femmes à risque et leur prise en charge précoce.

Il s'agit d'une évaluation du système de surveillance des lésions précancéreuses et cancéreuses du col de l'utérus réalisé sur les données de 2017 à 2019. Les données ont été collectées par revues documentaires et administration d'un questionnaire à l'endroit de tous les acteurs impliqués dans cette surveillance. L'analyse des données a été faite avec Epi Info 7.2.3.1 et Excel. Les résultats ont été présentés sous forme de tableaux et figures.

Entre 2017 et 2019, 25 854 clientes ont bénéficié du dépistage des lésions précancéreuses du col de l'utérus au niveau du centre national de la santé de la reproduction dont 775 étaient positives à l'IVA/IVL soit 2,99%. 150 d'entre elles soit 19,87% étaient âgées de 50 ans à plus. Notre évaluation a jugé ce système d'une qualité moyenne puisqu'il est moyennement sensible (Se=59,22%), simple et représentatif.

Fondée sur un processus incluant de nombreuses dimensions, la démarche d’évaluation d'un système de surveillance est utile et nécessaire afin d'optimiser la qualité des informations produites. Au Niger la surveillance des lésions précancéreuses et cancéreuses du col utérin est de type passif intégrée dans le paquet minimum d'activités des structures de santé.

# Research article: Grossesse des adolescentes au Sénégal: quelles complications pour le nouveau-né?

**Published:** 2021-11-30

**Authors:** M. Gueye, A. Sow, D. Boiro

**Affiliations:** Faculté de médecine de l'Université Cheikh Anta Diop de Dakar (UCAD), Dakar 10700, Sénégal

**Keywords:** Adolescente, Grossesse précoce, Complication, Nouveau-né, Dakar, Sénégal, Afrique subsaharienne

La grossesse précoce est associée à un risque élevé de complications maternelles et néonatales avec une morbimortalité néonatale importante surtout dans les pays en développement. L'objectif était d’évaluer la morbidité et d'analyser les causes de mortalité néonatale liées à ces grossesses précoces.

Il s'agissait d'une étude prospective et descriptive qui s'est déroulée du 1^er^ août 2016 au 31 janvier 2017 dans un centre hospitalier à Dakar. Etaient inclus les nouveau-nés issus de mères âgées de 19 ans et moins ayant accouché à la maternité.

Deux-cent-neuf parturientes ont été colligées sur un total de 2073 parturientes soit 10,08%. L’âge moyen était de 17,59 ans. On notait une maigreur dans 7,3% et un surpoids dans 19%. La grossesse était mal suivie dans 32,1%. Les complications obstétricales les plus rencontrées étaient la menace d'accouchement prématuré, la rupture prématurée des membranes (RPM), l'hypertension artérielle et la prééclampsie. Les indications des césariennes étaient dominées par les anomalies du bassin (28,4%) et la RPM (11,4%). Les complications néonatales les plus rencontrées étaient la prématurité 39,1%, la détresse respiratoire 12,7%, les infections materno-fœtales 12,3% et l'asphyxie périnatale 8,2%. La mortalité était de 8,2%. Le nombre de CPN inférieur à 4 était significativement associé aux décès néonataux.

La grossesse des adolescentes est pourvoyeuse de beaucoup de complications notamment chez le nouveau-né. Il est nécessaire de mener des campagnes de communications afin de les endiguer.

# Research article: Quels indicateurs de la surveillance de la grossesse et de l'accouchement à Oran (Algérie) en 2019?

**Published:** 2021-11-30

**Authors:** N. Heroual, N. Boukfoussa, Z. Yehia Zoubir, Z. Ahmed Fouatih, K. Terki

**Affiliations:** Faculté de médecine de l'Université Oran 1 Ahmed Ben Bella, BP 1510 El M'Naouer 31000 Oran (Ex: INESSM - Sis à Saint-Hubert), Algérie

**Keywords:** Grossesse, Suivi, Accouchement, Santé périnatale, Oran, Algérie, Afrique du Nord

La surveillance de la santé périnatale est une composante essentielle de la gestion du système de santé permettant ainsi d'améliorer l’état de santé des femmes enceintes, des mères et des nourrissons. En Algérie, des programmes de lutte contre la mortalité maternelle et infantile constituent une priorité de santé publique et des progrès considérables ont été réalisés pour atteindre les objectifs du millénaire. L'objectif de ce travail est de décrire les pratiques médicales pendant le suivi de la grossesse et l'accouchement.

Les données sont issues d'une étude multicentrique transversale réalisée pendant une semaine dans l'ensemble des maternités publiques de la wilaya d'Oran (13) incluant les parturientes ayant accouché d'un enfant né vivant et/ou mort-né. Les données sociodémographiques de la mère, le déroulement de la grossesse et l'accouchement ont été relevés.

Cinq-cent-quarante-sept femmes ont été enquêtées durant la période d’étude. L’âge moyen des parturientes était de 30 ans ± 6 [18 - 53 ans]. Plus de la moitié (58,8%) avait un niveau d'instruction moyen/secondaire et 22,3% un niveau supérieur. La moitié des parturientes avait consulté au moins une fois durant la grossesse et 4% des femmes n'ont eu aucun suivi. Sept femmes sur dix avaient reçu une supplémentation en fer et 58% en folates. Les grossesses à haut risque représentaient 6,5% et le taux de césariennes était de 41%.

Il s'agit d'une première enquête sur la santé périnatale réalisée dans la région. Elle a permis de décrire et de quantifier différents indicateurs d’évaluation des programmes de santé mis en place.

# Research article: E-Santé et la santé sexuelle et reproductive

**Published:** 2021-11-30

**Authors:** Corine Maurice Ouattara

**Affiliations:** Pass Santé Mousso, 11 BP 2428 Abidjan 11, Côte d'Ivoire

**Keywords:** Santé sexuelle, Femme, Innovation, Education, E-santé, Côte d'Ivoire, Afrique subsaharienne

Depuis quelques décennies, l’école ivoirienne est minée par les grossesses des jeunes filles. Dans la totalité des cas, il s'agit de grossesses non désirées dues à une précocité de l'activité sexuelle chez les élèves, les rapports sexuels non protégés, le manque d’éducation sexuelle à l’école, l'insuffisance de communication entre parents et enfants en matière de santé sexuelle.

Selon un rapport statistique consigné dans l'Annuaire statistique 2018-2019, Le nombre de grossesses en milieu scolaire est passé de 390 cas de 2017-2018 à 442 cas au titre de l'année académique 2018-2019, soit une hausse de 13,3%. Ces chiffres demeurent en constante progression plombant ainsi la réussite scolaire des filles.

La pandémie a perturbé l'accès aux soins, détourné l'attention des décideurs et des médias loin des priorités en matière de santé sexuelle et reproductive. Les confinements et autres mesures visant à ralentir la propagation de la Covid-19 ont eu des effets secondaires qui affectent négativement la santé des femmes et de la jeune fille, tels que l'augmentation de la violence domestique, des violences basées sur le genre, ou des interruptions dans l'accès aux soins contraceptifs et l'avortement sécurisé.

De nombreuses femmes et jeunes filles en milieu scolaire font face à des grossesses non-désirées.

Il est question de savoir comment la E-santé peut aider à faire changer les choses au niveau information et sensibilisation et améliorer la situation des femmes, jeunes filles et des élèves.

# Research article: Renforcement de la qualité des soins en santé sexuelle et reproductive: l'approche de Santé Sud

**Published:** 2021-11-30

**Authors:** Aurélie Dentan

**Affiliations:** Santé Sud, 200 bd National, Le Gyptis Bat N, 13003 Marseille, France

**Keywords:** Santé sexuelle et reproductive, Approche droits, Qualité, Monitoring

Santé Sud est une organisation qui a pour mission d'améliorer la santé dans le monde. Depuis plus de 30 ans, nous avons développé une connaissance fine des territoires sur lesquels nous opérons – l'Afrique de l'Ouest, le Maghreb, Mayotte et Madagascar. Nous n'opérons ni dans l'urgence, ni en remplacement mais construisons avec nos partenaires des systèmes de santé plus efficaces et vertueux, au niveau national et international.

La thématique santé sexuelle et reproductive (SSR) et lutte contre les violences basées sur le genre est l'une des priorités stratégiques de Santé Sud. L'organisation met en œuvre ses projets selon une approche féministe fondée sur les droits et l’égalité de genre, avec une méthodologie de santé publique visant la promotion d'un continuum de soins de qualité.

La présentation mettra en lumière:

1) D'une part la stratégie SSR de Santé Sud en détaillant:
  - Les éléments d'une stratégie de santé publique;
  - Comment mettre en œuvre une approche droit et genre;
  - Quelles interventions mettre en œuvre pour renforcer la qualité des soins en SSR.
2) D'autre part, Santé Sud s'est donnée pour objectif de renforcer les outils et pratiques internes de suivi-évaluation (monitoring) en développant des indicateurs précis, fiables et mesurables et en développant des outils de monitoring et de collecte de données permettant de mesurer en continu ces indicateurs. Notre communication inclura une présentation de l'outil de monitoring SSR développé par Santé Sud.

# Research article: Prévalence et facteurs associés à l'hépatite B chez les femmes ayant accouché dans le district sanitaire de Sotouboua au Togo (avril 2019 - mars 2020)

**Published:** 2021-11-30

**Authors:** P.K. Yanogo, P. Kpaikpai, N. Meda

**Affiliations:** Burkina Field Epidemiology and Laboratory Training Program (BFELTP), Ouagadougou, Burkina Faso

**Keywords:** Hépatite B, Prévalence, Facteurs associés, Sotouboua, Togo, Afrique subsaharienne

L'infection par le virus de l'hépatite B demeure un problème majeur de santé publique en Afrique. Au Togo, au moins 20% de la population sont infectés par l'hépatite B avec des préfectures dans le nord du pays dont la prévalence de l'infection est de l'ordre de 25 à 30%. Nous nous proposons de déterminer la prévalence et les facteurs associés à l'hépatite B chez les femmes ayant accouché dans le district sanitaire de Sotouboua avril 2019 à mars 2020.

Nous avons effectué une étude transversale analytique chez 179 femmes ayant accouché dans cinq formations sanitaires du district. Le sang veineux a été prélevé dans un tube sec et des données épidémiologiques ont été collectées. Les sérums ont été testés au REALY TECH et ECLIA électrochimiluminescence. La prévalence globale de l'hépatite B et la prévalence de l'hépatite B selon des caractéristiques socio démo graphique ont été calculées et testées par un test de Khi2. En analyse étiologique, nous avons, dans une régression logistique univariée puis multivariée, recherché les variables indépendamment associées à l'hépatite B; des OR ajustés avec IC95% ont été calculés. Les analyses ont été effectués par les logiciels Epi-info version 7.2.1 et Stata.

Sur 179 femmes ayant accouché d'avril 2019 à mars 2020 et testées, la prévalence de l'hépatite B était de 22,9%. L'antécédent familial d'hépatite B (ORa = 4,12 [1,28-13,28], p = 0.020), la scarification/tatouage (ORa = 3,28 [1.43-7.52], p = 0,01), et la profession ménagère (ORa = 5,31 [1,06-26,66], p = 0,03) étaient les facteurs indépendamment associés à l'hépatite B.

Notre étude montre que l'hépatite B demeure un problème de santé majeur dans le District sanitaire. Il est nécessaire de mener des campagnes de sensibilisation et d'agir sur ces facteurs associés pour réduire la prévalence.

# Research article: Évaluation de la disponibilité et de la gestion des médicaments vitaux prioritaires destinés aux femmes: étude transversale dans le district sanitaire de Guédiawaye (Sénégal)

**Published:** 2021-11-30

**Authors:** O. Bassoum, N.M. Sougou, A. Faye, M.M.M. Leye, I. Seck

**Affiliations:** Université Cheikh Anta Diop (UCAD), Dakar 10700, Sénégal

**Keywords:** Médicament vital prioritaire, Santé reproductive et maternelle, Guédiawaye, Sénégal, Afrique subsaharienne

Les médicaments vitaux prioritaires destinés aux femmes peuvent prévenir et traiter les principales causes des décès maternels tels que l'hémorragie du post-partum et la pré-éclampsie/éclampsie. Cette étude évaluait la disponibilité et la gestion de ces médicaments.

L’étude était transversale. Elle s'est déroulée dans les treize structures sanitaires publiques du district sanitaire de Guédiawaye. Ce dernier est situé dans la capitale sénégalaise. Quatorze produits de santé reproductive et maternelle étaient sélectionnés. Leur disponibilité le jour de l'enquête et durant les trois derniers mois était déterminée. La gestion des stocks était évaluée à travers la disponibilité des fiches de stocks et la présence de stocks périmés.

Les contraceptifs et les médicaments de santé maternelle étaient disponibles dans 82,1% et 24% des structures sanitaires. La durée de rupture était respectivement estimée à 12,1 et 34,5 jours. La disponibilité des fiches de stocks était de 100% pour les contraceptifs et 98,6% pour les médicaments de santé maternelle. Seuls des échantillons périmés de la pilule contraceptive d'urgence étaient trouvés dans 15,4% des structures sanitaires.

Cette étude a montré que la disponibilité des produits de santé reproductive était élevée contrairement à celle des médicaments de santé maternelle. La gestion des stocks était marquée par une bonne disponibilité des fiches de stocks et la faible présence d’échantillons périmés. Le renforcement des bonnes pratiques de gestion de stocks et de la chaîne de distribution seraient nécessaires.

# Research article: Ethnographie des logiques et pratiques structurant la sexualité des jeunes en milieu universitaire à Abomey-Calavi (Bénin)

**Published:** 2021-11-30

**Authors:** R.A. Houngnihin

**Affiliations:** Laboratoire d'anthropologie médicale appliquée (LAMA), Université d'Abomey-Calavi, Bénin

**Keywords:** Logiques, Pratiques sexuelles, Université, Jeunes, Abomey-Calavi, Bénin, Afrique subsaharienne

Longtemps traitée comme un sujet tabou, la sexualité fait actuellement l'objet d'un regain d'intérêt dans les pays africains. Elle est au cœur de plusieurs préoccupations sociétales: précocité des rapports sexuels, grossesses non désirées, infections sexuellement transmises, violences sexuelles, etc. Au Bénin, elle s'inscrit dans une pluralité de logiques et de pratiques. C'est dans ce cadre que la présente recherche a été initiée pour interroger les facteurs socioculturels, économiques et institutionnels qui interfèrent dans la sexualité des jeunes dans le contexte spécifique de l'Université d'Abomey-Calavi.

Le dispositif méthodologique repose sur une démarche qualitative ayant mobilisé les techniques telles que l'entretien semi-directif approfondi et l'observation. Au total, cinquante personnes appartenant à trois catégories socioprofessionnelles ont été interviewées: les étudiants, les parents et les enseignants.

Les travaux ont permis d'examiner le caractère pluridimensionnel du phénomène de la sexualité des jeunes, indépendamment des approches de santé publique ou de démographie. Cette sexualité s'intègre dans plusieurs logiques et de pratiques, généralement déterminées aussi bien par les normes et valeurs socioculturelles (perceptions sociales des relations sexuelles et du rôle assigné à l'activité sexuelle) que par le milieu dans lequel vivent les acteurs. De façon générale, les pratiques sexuelles à l'Université d'Abomey-Calavi reposent sur plusieurs logiques, allant de la recherche du bien-être psychologique à la quête économique. L'activité sexuelle est une stratégie de survie chez maints étudiant(e)s. La précarité semble donc avoir une interférence non négligeable dans les pratiques sexuelles qui subissent, par ailleurs, l'influence des rumeurs autour du sexe et de ses bienfaits, sans compter le rôle déterminant des pairs. Le contexte de sous-information ou de détention d'informations de mauvaise qualité par la plupart des jeunes, mérite d’être souligné. L'incitation à l'engagement des jeunes dans les rapports sexuels est renforcée par l'influence de la diffusion des modes de vie occidentale et le fait que le contrôle des parents et des aînés ait perdu un peu de son importance. Ces différents contextes font émerger une diversité de pratiques sexuelles telles que: la fellation et le cunnilingus (pour « fidéliser » le partenaire) et le recours à plusieurs partenaires sexuels à la fois. Dans cette perspective, l'utilisation du préservatif masculin oscille-t-elle entre oubli et dédain. Enfin, dans un contexte de famille en crise et en l'absence d'une politique étatique favorable à la sexualité responsable, les jeunes sont inaptes à maîtriser leur sexualité.

# Research article: Les achats stratégiques de soins dans l'amélioration des soins maternels dans trois districts sanitaires au sud du Tchad

**Published:** 2021-11-30

**Authors:** D. Manoufi, W.C. Kabore, C.N. Yahannon, A. Dumont, V. Ridde

**Affiliations:** Bureau d'appui santé et environnement (BASE), 80, rue 3044 Klemat 3^e^ Ard. BP. 1442 - N'Djamena, Tchad

**Keywords:** Santé maternelle et infantile, Santé communautaire, Exemption de paiement, Évaluation des effets, Séries chronologiques interrompues, Afrique subsaharienne

Le Tchad figure parmi les pays où la mortalité maternelle et infantile est la plus élevée du monde. Une intervention a été mise en œuvre de 2016 à 2019 dans trois districts sanitaires ruraux au Sud du Tchad, avec une importante collaboration des communautés, pour améliorer la santé des mères et des enfants en renforçant l'offre et la demande de soins de manière concomitante. L'objectif de cette étude est d’évaluer les effets de cette intervention sur l'utilisation des services de santé.

Des analyses de séries chronologiques interrompues avec groupe contrôle ont été utilisées pour mesurer les effets de l'intervention selon une approche quasi-expérimentale. Les données mensuelles de fréquentation ont été collectées à partir des registres de 18 centres de santé ayant participé à l'intervention et de 18 centres sans intervention extérieure (groupe contrôle), avant (18 mois) et après (24 mois) le début de la mise en œuvre de l'intervention.

En moyenne, il y a eu 10,98 consultations pédiatriques (IC à 95%: 6,57-15,39, p<0,001) et 0,68 accouchements (IC à 95%: 0,42-0,95, p<0,001) supplémentaires chaque mois dans le groupe d'intervention comparé à l’évolution dans le groupe contrôle. L'implication des communautés a eu un rôle déterminant dans cette évolution.

Au cours des 24 mois de mise en œuvre, l'intervention a permis d'accroître significativement l'utilisation des services de santé essentiels à la santé des mères et des enfants au Tchad. Cette étude met en évidence les avantages d'un fort partenariat avec les communautés formées et impliquées dans le système de santé pour promouvoir la couverture santé universelle.

# Research article: Contribution à la lutte contre les violences faites aux femmes des couples infertiles dans les populations vulnérables du Burundi

**Published:** 2021-11-30

**Authors:** C. Fabin, C. Joffin, J.-N. Joffin, M. Hakizimana

**Affiliations:** ASSITEB, 33 Rue de Metz, 94170 Le Perreux-sur-Marne, France

**Keywords:** Infertilité, Violence, Femme, Laboratoire, Prévention, Formation, Burundi, Afrique subsaharienne

Dans un pays, comme le Burundi où le nombre moyen d'enfants par femme est de plus de 5, un couple sans enfant n'est pas la norme et la responsabilité de l'infertilité du couple est habituellement attribuée à la femme. Afin de contribuer à réduire le rejet, voire la répudiation des femmes de ces couples, le consortium composé de l'Association Internationale des technologistes biomédicaux, ASSITEB-BIORIF, de l'Association nationale des technologistes biomédicaux du Burundi, ANTEBBU, et de l'Association burundaise des sages-femmes, ABUSAFE, prévoit de sensibiliser les populations vulnérables burundaises sur le rôle du laboratoire dans la prise en charge des couples infertiles.

En effet, selon l'OMS, l'infécondité concerne des millions de personnes en âge de procréer et la proportion de couples infertiles serait entre 15 à 30% en Afrique. Dans 40% des cas, l'origine de l'infertilité est infectieuse.

Si le rôle du laboratoire, dans la lutte contre l'infertilité des couples, est évident dans la réussite de la naissance d'enfants par fécondation in vitro (plus d'un millier en Afrique depuis Tommy en 1998), au quotidien le laboratoire est un appui dans la démarche préventive, diagnostique et curative d'aide à la procréation.

Après une formation des personnels de laboratoire venant de toutes les provinces du Burundi en biologie sexuelle et reproductive, le consortium diffusera une information sur «biologie et fertilité» selon les voies suivantes:

- affiches dans les laboratoires et dans les centres de santé en français et en kirundi
- causeries dans les villages
- interventions auprès des media, journaux, radios et télévision.

Parmi les messages seront valorisées les informations scientifiques sur les causes masculines et/ ou féminines de stérilité, les explorations biologiques et le rôle du laboratoire dans les modalités de prise en charge préventives et curatives.

Une information large relayée par des professionnels de santé formés devrait contribuer à déculpabiliser des couples sans enfant, surtout les femmes, à admettre que l'origine de l'infertilité d'un couple n'est pas automatiquement due à la femme, qu'une prise en charge du couple est possible et que fécondité et sexualité ne sont pas équivalentes.

De plus, l'information des communautés, notamment des belles familles et l'exemple au plus haut niveau de l’état burundais tant politique que sanitaire devraient permettre un renforcement de ces messages, y compris pour les populations vulnérables, pour contribuer à lutter contre les violences faites aux épouses sans enfant et inciter à des consultations et prises en charge le plus précocement pour les couples infertiles.

# Research article: Étude descriptive sur le cancer du col de l'utérus dans la wilaya d'Oran: résultats de 22 ans d'enregistrement (1996-2017)

**Published:** 2021-11-30

**Authors:** F.Z. Besbes, H.B.M. Messid, F. Ammour, M. Raiah, L. Benrabah, N. Guetarni, N. Heroual, N. Hebali, A. Bouhraoua, F. Chouaf, N. Bensirat, S. Adda, M.H. Kadiri, A. Benjoudi, A. Lounis, Z. Ahmed Fouatih, K. Terki

**Affiliations:** Service d’épidémiologie et de médecine préventive, CHU Oran, 1 place Roux - Oran, Algérie

**Keywords:** Cancer du col de l'utérus, Incidence, Registre du cancer, Oran, Algérie, Afrique du Nord

Dans le monde, le cancer du col de l'utérus est le deuxième cancer féminin après le cancer du sein. C'est la troisième cause de mortalité par cancer chez la femme. Les carcinomes épidermoïdes représentent la morphologie la plus fréquente des cancers du col de l'utérus. En Algérie, le cancer du col se place en deuxième position après celui du sein. C'est un véritable problème de santé publique. Les répercussions de cette néoplasie sont lourdes au plan socio-économique et sanitaire. Les cancers du col sont le plus souvent vus à des stades avancés et impliquent de ce fait une prise en charge importante, très coûteuse et souvent inefficace.

Les objectifs de cette étude sont de: (i) décrire les caractéristiques épidémiologiques du cancer du col de l'utérus à Oran; (ii) évaluer l'incidence du cancer du col de l'utérus à Oran de 1996-2017.

Population et méthodes: Étude descriptive rétrospective; registre du cancer d'Oran; recueil actif; années d'enregistrement: 1996-2017; logiciels Canreg version 4 et Epi Info 6.

À Oran, le cancer du col de l'utérus est le deuxième cancer gynécologique. L’âge moyen est d'environ 55 ans. La tranche d’âge la plus touchée est celle des 50-54 ans, suivie de celle des 45-49 ans. Le taux d'incidence brut = 9,7/100 000 habitant. La morphologie la plus fréquente est le carcinome épidermoïde.

# Research article: Deugueur Bop ! Le podcast des femmes audacieuses

**Published:** 2021-11-30

**Authors:** T. Cosnier, C. Gilardoni

**Affiliations:** Ambassade de France, 1 rue El Hadji Amadou Assane Ndoye, Dakar, Sénégal

**Keywords:** Hygiène menstruelle, DSSR, Violences basées sur le genre, Féminisme, Dakar, Sénégal, Afrique subsaharienne

En onze épisodes, Deugueur Bop, la « Tête Dure », présente des témoignages et récits de vie de femmes sénégalaises inspirantes.

Engagées, drôles, fortes, passionnantes, chacune à sa manière nous dévoile sa vision de la place des femmes dans la société sénégalaise et propose des nouvelles manières de la réinventer.

Un voyage dans Dakar et dans le quotidien des femmes au Sénégal, loin des idées reçues.

Trois épisodes sont consacrés à la santé sexuelle et reproductive au Sénégal:

- Un épisode sur l'hygiène menstruelle, « Changer les Règles », avec trois jeunes activistes engagées dans la promotion de la SSRAJ, Madame Rose Dièmè de Youth Women for Action, Madame Christine Kiné Sarr, coordinatrice nationale de SheDecides et Madame Seynabou Sène. Cet épisode a été enregistré avec des jeunes filles de l'Ecole Kléber de Dakar.
- Un épisode sur la lutte contre les violences basées sur le genre, avec la psychiatre Khaira Thiam, la sociologue Laïty Ndeye et Yacine Diouf, fondatrice d'un centre d'accueil dédié aux femmes victimes de violences et de privation de DDS – Kayam.
- Un épisode sur la masculinité positive enregistré au Réseau des Jeunes contre l'excision et les mariages d'enfants, ardemment engagé dans la promotion de la SSRAJ.

D'autres épisodes promeuvent la SSR au Sénégal, comme l’épisode sur la dépigmentation et les dangers des canons de beauté importés.

Les épisodes ont été enregistrés dans le cadre de la préparation du Forum génération égalité, dont la France copréside la coalition d'action « DSSR » avec le Burkina Faso.

# Research article: Détection et génotypage des papillomavirus humains à haut risque dans des prélèvements génitaux au niveau de la région d'Alger

**Published:** 2021-11-30

**Authors:** L. Boublenza, H. Mebarki, I. Lallama, N. Sadouki, D. Mohammedi

**Affiliations:** LAMAABE, Département de biologie, Faculté SNV STU, Université de Tlemcen, BP 230, 13000 Chetouane Tlemcen, Algérie

**Keywords:** HPV-HR, Génotypage, Lésion précancéreuse, Alger, Afrique du Nord

Le papillomavirus humain à haut risque (HPV-HR) est l'agent étiologique du cancer du col de l'utérus, troisième cancer de la femme en Algérie, ce qui constitue un véritable problème de santé publique au moment où son incidence diminue dans les pays développés.

L'objectif de cette étude a été porté sur la détection et le génotypage des HPV à haut risque (HPV-HR) dans des lésions précancéreuses, réalisée au niveau du laboratoire herpes virus, papillomavirus et autres de l'institut Pasteur d'Algérie.

Un total de 43 prélèvements cervicaux ont fait l'objet de cette étude. La mise en évidence de l'ADN viral des HPV-HR avec génotypage partiel (HPV 16 et 18) a été réalisée par l'utilisation du système Cobas^®^ 4800 suivie par un génotypage par le kit Linear Array^®^ des autres HPV-HR détectés par le premier test.

La moyenne d’âge des participantes à cette étude était de 49 ans présentant principalement des frottis de types LSIL, ASC-US avec des pourcentages de 73%, 23% respectivement. Au total trois prélèvements étaient positifs en HPV-HR, soit un taux de 7%. Les types viraux oncogènes trouvés ont été le HPV 18, 56 et 58.

Ces résultats préliminaires nous incitent à mettre en place une étude portant sur un échantillonnage plus large de patientes, incluant l'examen cytologique et le test HPV; ce qui pourrait être utile pour évaluer le rapport coût-efficacité du vaccin contre l'HPV et son utilisation lors d'un programme national de prévention contre le cancer du col.

# Research article: Analyse du profil épidémiologique et clinique des patients consommateurs d'alcool suivis au centre de prise en charge intégrée des addictions de Dakar (2015-2018) (Sénégal)

**Published:** 2021-11-30

**Authors:** M. Leye, M. Makhtar, B.Y. Ndiaye, I. Ba, I. Seck

**Affiliations:** Université Cheikh Anta Diop (UCAD), Dakar 10700, Sénégal

**Keywords:** Prévention, Alcool, Dépendance, Addiction, Dakar, Sénégal, Afrique subsaharienne

La consommation d'alcool ne cesse d'augmenter dans le monde. Le Sénégal n'est pas épargné par ce fléau. L'objectif de cette étude est de déterminer le profil épidémiologique et clinique des usagers d'alcool chez les patients suivis au centre de prise en charge intégrée des addictions de Dakar (CEPIAD).

Une étude transversale descriptive et analytique a été menée du 13 juin au 20 août 2019.

La population d’étude était l'ensemble des dossiers des patients suivis pour usage d'alcool au niveau du CEPIAD du Sénégal. Un recrutement exhaustif de l'ensemble des dossiers répondant aux critères d'inclusion de 2015 à 2018 a été mené. Les données ont été analysées à l'aide des logiciels Epi Info et R.

L’étude portait sur 245 patients suivis au CEPIAD pour usage d'alcool. Les patients de sexe masculin prédominaient (95,10%). L’âge des patients variait entre 17 et 69 ans avec une moyenne d’âge de 39,68 ans. Près de la moitié des patients étaient sans enfant (48,1%). La quasi-totalité des patients 97,7% était instruite. La majorité des patients étaient au chômage les 6 derniers mois (37,14%). Près de la moitié des patients (48,98%) n'avaient pas donné d'information concernant leur enfance, 46,83% des patients ont eu à séjourner à l’étranger. L'addiction était sévère chez 86,1% patients. Les facteurs associés à l'addiction sévère à l'alcool étaient le fait d’être au moins deuxième de la fratrie ORa 2,9 = [1,3-7,0], le fait de boire au moins quatre verres par jour ORa 3,4 = [1,4-8,5], le fait d'avoir une pathologie organique ORa 5,3 = [1,2-23,7].

La survenue de l'addiction sévère chez les consommateurs d'alcool doit pousser les autorités sanitaires à mener des stratégies efficaces pour y faire face dans une approche multidisciplinaire. La sensibilisation de la population aux méfaits de l'alcool, couplée à des mesures réglementaires sur la vente de l'alcool seraient des armes majeures contre ce fléau.

# Research article: Brûlures graves, plaies étendues et recouvrement cutané au Tchad. Présent et perspectives

**Published:** 2021-11-30

**Authors:** H. Lefort, P. Mulumba, O. Ngaringuem, G. Thomas, N.A. Mahamat, D.N. Ndifusah, H.T. Tyau

**Affiliations:** Hôpital d'instruction des armées Laveran, 34 Boulevard Laveran, 13384 Marseille, France

**Keywords:** Brûlure, Plaies, Greffe, Prise en charge, Tchad, Afrique subsaharienne

Le Tchad appartient à la région sahélo-saharienne, situé au cœur de l'Afrique. Le risque de plaie et de brûlure y est particulièrement présent. Les étiologies sont diverses et peu originales. Le défaut d’éducation à l'hygiène, un réseau de soins peu dense ainsi qu'un recours aux tradipraticiens sont autant d'exemples de causes à l'origine d'un retard de prise en charge, de tableaux cutanés délabrés extensifs source d'une morbimortalité non évaluée à ce jour. Les soignants sont ainsi régulièrement confrontés, chez l'adulte comme l'enfant, à des plaies et brûlures graves qui ne peuvent trouver d'issue qu'après des soins de cicatrisation dirigée lourds et longs, cela d'autant plus si les solutions de recouvrements cutanées sont rares et les parcours de soins non identifiés.

Nous rapportons la synthèse d'un travail original, réalisé en quelques semaines, guidé par les problématiques rencontrées dans l'exercice quotidien des soins aux populations, parfois face à des situations cutanées catastrophiques engageant le pronostic vital à court terme du patient. Nous décrivons les moyens humains, logistiques, les techniques à disposition des acteurs principaux de la prise en charge des plaies et brûlures graves sur le territoire tchadien. Nous rapportons comment la construction d'un réseau de soins a démontré sa pertinence immédiate, à moindre coût, mais aussi ses avantages et limites. In fine, notre discussion ouvre sur les perspectives envisageables pour le Tchad, en termes de réseau de soins, de formation des soignants et de santé publique. Un tel travail d'expertise pourrait trouver sa pertinence dans d'autres pays d'Afrique.

# Research article: Associations entre le risque de cancer gastrique et l'infection virale: étude sur une population de l'ouest algérien

**Published:** 2021-11-30

**Authors:** D. Behar, L. Boublenza, N. Chabni, B. Dahmani, N. Masdoua, A. Nahet, K. Meguenni

**Affiliations:** LAMAABE, Département de biologie, Faculté SNV STU, Université de Tlemcen, BP 230, 13000 Chetouane Tlemcen, Algérie

**Keywords:** Cancer gastrique, Virus du papillome humain, Virus d'Epstein-Barr, Facteur de risque, Algérie, Afrique du Nord

Le cancer gastrique est l'un des types de cancer les plus courants et l'une des principales causes de mortalité associée au cancer dans le monde. Selon les données de GLOBOCAN 2018, le cancer de l'estomac est le 5^e^ néoplasme le plus courant et le 3^e^ cancer le plus mortel, avec environ 783 000 décès. La répartition géographique du cancer gastrique est caractérisée par une large variation internationale; les zones à haut risque comprennent l'Asie de l'Est (Chine, Japon), l'Europe de l'Est et certaines parties de l'Amérique centrale et du Sud. Plusieurs facteurs de risque ont été identifiés pour le cancer gastrique La cause la plus importante et la plus fréquente est la consommation excessive de sel, le tabagisme, la sujétion à long terme aux nitrosamines dans les aliments et l'eau potable, ainsi que les infections microbiennes, telles que *Helicobacter pylori* et certains virus, dont Le virus d'Epstein-Barr (EBV) et le virus du papillome humain (HPV).

Le virus d'Epstein-Barr est très courant dans la population générale et est également connu pour être présent dans une petite proportion des tissus du cancer gastrique. Certaines méta-analyses ont conclu que le cancer gastrique EBV-positif pourrait apparaître chez 6,9% à 8,8% de tous les patients atteints d'un cancer gastrique, ainsi que certaines études ont étudié l'infection par le HPV dans le cancer gastro-intestinal, mais seules des recherches sporadiques ont rapporté la prévalence du HPV dans le cancer gastrique.

Une efficacité de 31 blocs de cancer gastrique fixés au formol et inclus en paraffine a été étudiée. L'extraction de l'ADN a été réalisée à l'aide d'un kit QIAGEN. Après l'extraction de l'ADN de chaque bloc de paraffine, nous avons effectué une PCR suivie d'une électrophorèse.

Notre étude montre que les infections par le papillomavirus humain et le virus Epstein Barr ne sont pas un facteur de risque de développement d'un cancer gastrique, d'autres études à grande échelle sont importantes pour confirmer nos résultats.

# Research article: Profil épidémiologique du VIH/sida dans l'ouest de l'Algérie: 5 années de notification (2014-2018)

**Published:** 2021-11-30

**Authors:** F. Ammour, N. Guetarni, N. Hebali, N. Mouffok, F.Z. Ahmaed, K. Terki

**Affiliations:** Université Oran 1 Ahmed Ben Bella, BP 1510 El M'Naouer 31000 Oran (Ex: INESSM - Sis à Saint-Hubert), Algérie

**Keywords:** VIH, Sida, Épidémiologie, Notification, Oran, Algérie, Afrique du Nord

L’épidémie de sida est très dynamique, elle continue à croître et se modifie au fur et à mesure que le virus exploite de nouvelles possibilités de transmission. En matière d'infection à VIH, l'Algérie est placée parmi les pays peu actifs avec une prévalence inférieure à 0,1%. De 2010 à 2014, le nombre de nouveaux cas d'infections à VIH diagnostiqués dans notre pays est relativement stable évoluant entre 700 et 800 cas en moyenne par an. Au CHU d'Oran, le centre de référence pour le diagnostic et la prise en charge couvre toute la région ouest et sud-ouest, cependant depuis 2014, d'autres centres de prise en charge ont été créés dans la région (Sidi Bel Abbes et Tlemcen).

L'es objectifs de l’étude sont de connaître le nombre de cas incidents identifiés et pris en charge par le service des maladies infectieuses du CHU d'Oran (service de référence) et d’étudier les caractéristiques des patients infectés.

L’étude est de type descriptif rétrospectif. Elle a porté sur les cas de VIH/sida déclarés à notre service durant la période de 2014 à 2018 par le service référent de la région ouest de l'Algérie (service des maladies infectieuses du CHUO). La base de données des MDO a servi comme source d'information. L'analyse est faite sur le logiciel Epi-info V3.5.3.

Entre 2014 et 2018, 3035 nouveaux cas ont été déclarées au SEMEP du CHU d'Oran, la femme est plus touchée avec un sex-ratio F/H de 1,1, la tranche d’âge la plus infectée par cette maladie est de 30 à 34 ans voir entre 25 et 44 ans, on constate une nette augmentation du nombre de cas chaque année: de 424 cas en 2014 à 584 en 2018, dans l'Ouest algérien, la Wilaya d'Oran reste la plus touchée par cette épidémie suivie de Mascara et Tiaret.

Le nombre annuel de nouveaux cas déclarés dans notre région semble élevé. Le Laboratoire national de référence VIH/sida (LNR) a rapporté 845 nouveaux cas au niveau national en 2014, alors que 412 nouveaux cas ont été déclarés durant la même année par le service des maladies infectieuses du CHUO qui couvre l'ouest du pays. Ceci peut être expliqué par l'adhésion de la population au dépistage suite aux actions de sensibilisation qui se font sur le terrain. Le nombre de cas de VIH est sous-estimé, des enquêtes de séroprévalence au sein de la population générale peuvent produire de meilleures estimations.

# Research article: Profil épidémiologique de la rougeole au Niger(2010-2019)

**Published:** 2021-11-30

**Authors:** H. Ide, P.K. Yanogo, I. Djibo, D. Barry, O.B. Togola, F. Diallo, N. Meda

**Affiliations:** Burkina Field Epidemiology and Laboratory Training Program (BFELTP), Ouagadougou, Burkina Faso

**Keywords:** Rougeole, Surveillance épidémiologique, Base de données, Niger, Afrique subsaharienne

La rougeole est une maladie en forte progression dans le monde avec 869 770 cas et 207 500 décès enregistrés en 2019. Le Niger continue de faire face à des flambées épidémiques malgré les actions entreprises par l’État et ses partenaires. L'objectif de notre étude était d'analyser la base de données nationale de surveillance de la rougeole sur la période de 2010 à 2019 afin de caractériser l’épidémiologie de la rougeole au Niger et d'identifier les facteurs associés.

Nous avons conduit une étude transversale analytique incluant tous les cas suspects et positifs de rougeole de la base de données de la Direction de la Surveillance et de la Riposte aux Epidémies durant 10 ans. Nous avons utilisé le logiciel Epi-Info 7.2.3.1 pour l'extraction et l'analyse des données. Les fréquences et proportions ont été calculées et, nous avons dans une régression logistique estimé les OR d'association avec leur intervalle de confiance à 95%, les facteurs indépendants à la rougeole au seuil de p<0,05.

Nous avons trouvé 11784 cas suspects de rougeole notifiés de 2010 à 2019 dont 37,2% positif aux IgM. L'incidence de la rougeole était de 23,81 cas pour 1.000.000 habitants. Le sex-ratio Femme/Homme était de 1.1. Le groupe d’âge 1 à 5 ans était le plus représenté (44,44%). Toujours parmi les cas suspects, 28,3% ont reçu au moins une dose de vaccin, 62,22% vivaient en milieu urbain, 66,14% des districts ont notifié au moins un cas suspect avec prélèvement de sang dans l'année et 70,38% des échantillons sont reçus au laboratoire dans les 72 heures. Le nombre de décès enregistré pendant 10 ans était de 225 soit une létalité de 1,9%. En analyse étiologique, les facteurs associés à la rougeole parmi les cas confirmés étaient l’âge OR= 1.16 [1.07-1.24] p<0,001, le milieu de résidence OR= 1.8 [1,4-2,4] p<0,001 et le statut vaccinal OR= 0.45 [0.40-0.52] p<0,001.

L'incidence de la rougeole est élevée au Niger avec des cas et des décès enregistrés dans toutes les régions surtout chez les moins de 5 ans avec un faible taux de vaccination à 28,3%. L'amélioration de la riposte et des stratégies de vaccination sont recommandés en vue de réduire le fardeau lié à la rougeole.

# Research article: La charge des épidémies du choléra au Niger: une analyse des données de la surveillance nationale du choléra (2011-2016)

**Published:** 2021-11-30

**Authors:** M. Mahamadou Yacouba, A. Ibrahim, S. Tiendrebeogo, P. Yanogo, J. Kaboré, A. Yacouba, N. Meda

**Affiliations:** Burkina Field Epidemiology and Laboratory Training Program (BFELTP), Ouagadougou, Burkina Faso

**Keywords:** Choléra, Épidémie, Surveillance, Niger Afrique subsaharienne

Le choléra constitue un problème de santé publique majeur dans les pays en développement. La charge et les conséquences des épidémies de choléra sont très importantes en Afrique subsaharienne. Au Niger depuis 1990, des épidémies importantes du choléra ont été enregistrées presque chaque année. La plus récente remontait en 2018. Plusieurs stratégies ont été retenues dans la lutte contre ces épidémies du choléra au Niger. Ces stratégies se sont révélées inefficaces et considérées comme insuffisantes. Au regard de la résurgence des flambées épidémiques du choléra et des stratégies de lutte inefficaces mais aussi et surtout pour compléter des études qui ont été menées dans certaines régions du Niger nous avons décidé d'analyser la base de données du choléra sur le plan national.

Nous avons mené cette étude transversale descriptive sur la base des cas de choléra enregistrés au Niger de 2011 à 2016. Le diagnostic de ces cas a été posé sur la base des tests de diagnostic rapide du choléra au début de chaque épidémie et cliniquement à travers le lien épidémiologique par la suite. La plupart de ces cas ont été notifiés à la Direction de la surveillance et de la riposte aux épidémies (DSRE) et confirmés par le Centre de recherche médicale et sanitaire (CERMES). Ces données ont été collectées puis apurées avec ExcelR pour une assurance qualité. Une analyse temporo spatiale de ces données a été faite à l'aide du logiciel Epi-infoR et ExcelR. Les cartes ont été confectionnées à l'aide du logiciel QGISR (https://www.qgis.org). Les résultats ont été présentés sous forme de tableaux, figures et cartes.

Au total, 10 423 cas de choléra ont été enregistrés au Niger de 2011 à 2016 avec 268 décès soit une létalité de 2,57%. Des fortes incidences ont été enregistrées sur cette période allant parfois à plus de 50 cas pour 100 000 habitants. De 2011 à 2016, 856 échantillons de selles ont été prélevés et envoyés pour culture au CERMES. Sur les 856 selles cultivées, 384 *Vibrio cholerae* ont été isolées dont 370 (43,22%) *Vibrio cholerae* sérogroupe O1 sérotype Ogawa et 14 (1,63%) Vibrio cholerae sérogroupe O1 sérotype Inaba.

Le choléra constitue une affection endémo-épidémique au Niger. La souche responsable de cette maladie est en grande partie le *Vibrio cholerae* sérogroupe O1 sérotype Ogawa.

# Research article: Investigation des cas de rougeole, aire de santé de Diouna, district sanitaire de Ségou (Mali, septembre 2020)

**Published:** 2021-11-30

**Authors:** M.S. Diarra, Y. Ballayira, P.K. Yanogo

**Affiliations:** Burkina Field Epidemiology and Laboratory Training Program (BFELTP), Ouagadougou, Burkina Faso

**Keywords:** Investigation, Épidémie, Rougeole, Diouna, Ségou, Mali, Afrique subsaharienne

En 2018, le Mali a notifié 1 485 cas de rougeole dont 4 décès chez les enfants de moins de 5 ans. Un nombre anormalement élevé de cas d’éruptions cutanées non vésiculeuses a été enregistré dans un hameau de l'aire de santé de Diouna dans le district sanitaire de Ségou, Mali d'où l'intérêt d'une investigation pour comprendre le phénomène, stopper l’épidémie de rougeole et mettre en place de contrôle et prévention.

Une étude transversale descriptive a été effectuée dans l'aire de santé de Diouna du 27 juillet au 5 octobre 2020. Les données ont été saisies et analysées avec les logiciels Epi-info version 7.2 et Excel 2016 en temps, lieu et personnes Les résultats ont été résumés en proportions pour les données qualitatives et médiane pour les données quantitatives.

Trente-trois cas suspects ont été notifiés par l'aire de santé avec 18 échantillons prélevés. Au total, 26 cas de rougeole ont été diagnostiqués dans le hameau de Konokounwèrè, village de Tiécourabougou, zéro décès dont 11 confirmés au laboratoire et 15 par lien épidémiologique. Les enfants de moins de 5 ans représentaient 58% avec un âge médian de 4 ans [1; 12] ans et 73,1% pour le sexe masculin. La plupart des cas a été observé à la 37^e^ semaine 2020. Aucun enfant n’était vacciné dans le hameau. La couverture vaccinale administrative dans l'aire de santé en vaccin antirougeoleux1 en 2019 était en dessous de 95%.

L'investigation a permis de confirmer l’épidémie de rougeole, de constater que la couverture vaccinale était à 0% confortant une évidence qu'en absence d'immunisation, les épidémies seront récurrentes au sein de ces populations.

# Research article: Changement climatique et défis de santé des populations de la région de l'extrême-nord du Cameroun

**Published:** 2021-11-30

**Authors:** P. Njouonkou, S. Mvoa, T. Kandem, P. Mache, E. Ndougsa

**Affiliations:** Riposte au Covid-19, District de santé de la Cité Verte, VFGQ+FV5, Yaoundé, Cameroun

**Keywords:** Changement climatique, Santé humaine et animale, Défis, Cameroun, Afrique subsaharienne

Le changement climatique qui constitue de plus en plus une nouvelle menace importante pour la santé publique pourrait entraîner 250 000 décès supplémentaires par an entre 2030 et 2050 selon l'OMS (2017). Cette étude s'intéresse à l'influence négative des changements climatiques sur l’état de santé des populations de la région de l'extrême-nord du Cameroun entre 2015 et 2019. Un climat chaud et semi-aride, ainsi qu'une pluviométrie élevée (760 mm de pluie par an) y règne. Ce qui engendre les inondations, les canicules, les sécheresses extrêmes et les tempêtes. Défis des changements climatiques auxquels fait face la région de l'extrême-nord du Cameroun. Selon l'OMS, la température globale augmente d'environ 0,18oC par décennie entraînant des conséquences qui s'intensifient avec le temps tant sur la santé humaine qu'animale. Toutes ces manifestations physiques des changements climatiques se multiplient et leur impact socio économique est à explorer. Fort de ces constats, cette étude s'intéresse à déterminer l'influence négative des changements climatiques sur l’état de santé des populations de la région de l'extrême-nord du Cameroun entre 2015 et 2019. Plus spécifiquement, il est question de dresser un état des lieux climatique de la région de l'Extrême-Nord du Cameroun; d'analyser les rapports de l'Observatoire national sur les changements climatiques et enfin de dégager les conséquences de ce changement climatique sur la santé des populations de la région de l'extrême-nord du Cameroun. Cette étude est descriptive rétrospective, avec une approche mixte alliant les méthodes quantitatives et qualitatives. Les outils de collecte utilisés ont été la base des données des MAPE et la revue documentaire des rapports mensuels et bulletins décadaires et saisonniers. Les résultats quantitatifs ont ressorti dix maladies considérées comme problème de santé de santé publique. Il s'agissait du choléra (583 cas confirmés, 38 décès); les diarrhées sanglantes (9 496 cas,13 décès), de la fièvre Jaune (1 253 cas confirmés, 13 décès), de la fièvre typhoïde (115.414 cas confirmés, 27 décès), des GEAS (34.587 cas confirmés, 59 décès), du paludisme (1 638 881 cas confirmés, 2 865 décès), du syndrome grippal (75 685 cas confirmés, 24 décès), MAS (157 468 cas, 368 décès), des cas de MAS avec complications (23 725 cas, 506 décès), et enfin des Méningites (1 751 cas confirmés, 90 décès).

# Research article: Fixation et circulation de *Plasmodium vivax* à Madagascar

**Published:** 2021-11-30

**Authors:** D. Randriamiarinjatovo, S. Razanatsiorimalala, M. Randrianarivelojosia

**Affiliations:** Institut Pasteur de Madagascar, Antananarivo, Madagascar

**Keywords:** *Plasmodium, P. vivax*, Diagnostic, PCR en temps réel, Madagascar, Océan Indien

Le paludisme à *Plasmodium falciparum* est prédominant dans l’île de Madagascar. Cependant, une meilleure compréhension de la répartition géographique des autres espèces plasmodiales dont *P. vivax*, productrice d'hypnozoïte aide dans l'amélioration de la stratégie de diagnostic et de traitement en vue de l’élimination de cette maladie. Ainsi, nous avons détecté par PCR en temps réel, avec amplification du gène ARNr 18s, les infections à *P. vivax* et à *P. falciparum* dans différentes zones: sur la marge est des hautes terres centrales (village de Saharevo, suivi communautaire de 1996 à 2005), dans la région nord-ouest (commune d'Antanimbary, patients symptomatiques en 2017) et dans le sud (district d'Ivohibe, enquête transversale en population en 2019). L'ADN a été extrait à partir des échantillons de sang archivés à l'Institut Pasteur de Madagascar, en utilisant le kit Qiagen. L'infection plasmodiale a été confirmée dans 402/557 échantillons à Saharevo [72.2%; IC95%: 68.2 - 75.8%], dans 176/372 échantillons à Antanimbary [47,3%; IC95%: 42,1 - 52,5%] et dans 225/724 échantillons à Ivohibe [31.1%; IC95%: 27.7 - 34.6%]. Nos résultats démontrent que *P. vivax* est présent dans 97/803 des cas de paludisme confirmé [12.1%; IC95%: 9.9 - 14.6%]. Dans l'ensemble, 62.9% des infections plasmodiales ont été détectées chez les enfants de 5 mois à 14 ans. En guise de conclusion, si la fixation de *P. vivax* dans le moyen ouest de Madagascar est connue (pour ne citer que les foyers à *P. vivax* dans les districts de santé à potentiel agricole et minier de Miandrivazo et de Maevatanana), nos résultats confirment la circulation de cette espèce dans des régions aux faciès de transmission différents à Madagascar. Ces données sont utiles et utilisables pour améliorer la prise en charge du paludisme. En plus de l’étude génétique de *P. vivax* dans notre perspective, le choix des tests de diagnostic rapide et des antipaludiques adaptés à la situation à Madagascar sera discuté dans notre présentation.

# Research article: Vecteurs de *Plasmodium* spp et indicateurs entomologiques six mois après aspersion intra-domiciliaire de pyrimiphos-méthyl (Actellic 300 CS) à Ankililoaka (Toliara II, Madagascar)

**Published:** 2021-11-30

**Authors:** T. Rakotondranaivo, G.T. simihefozafy, A. Indriambelo, J.D. Mahatombo, N. Tiava, M. Randrianarivelojosia

**Affiliations:** Institut Pasteur de Madagascar, Antananarivo, Madagascar

**Keywords:** *Anopheles*, Pyrimiphos-methyl, Insecticide, Ankililoaka, Madagascar, Océan Indien

La politique nationale de lutte contre le paludisme à Madagascar repose essentiellement sur la distribution de moustiquaires imprégnées d'insecticides à longue durée d'action, et les campagnes de pulvérisation intra-domiciliaire d'insecticides (CAID). À Ankililoaka une CAID utilisant pyrimiphos-methyl (Actellic 300 CS) a eu lieu en janvier 2018. Il s'agit d'un village dans le pôle rizicole du district de santé de Toliara II, dans le sud subaride du pays avec des gîtes larvaires permanents favorables aux *Anopheles.* Les anophèles vecteurs cibles étaient ceux connus comme endophiles et endophages comme *Anopheles funestus.* Ainsi, l'efficacité de la CAID peut se traduire par la disparition de cette espèce. Des travaux ont été entrepris à Ankililoaka pour détecter la présence d'anophèles vecteurs dans la faune résiduelle en juin et en août 2018. Les captures de moustiques ont été effectuées par aspersion de pyréthrine à l'intérieur des habitations entre 6 et 9 heures du matin pendant trois jours dans 18 maisons tirées au sort. Parmi les moustiques collectés, les individus du groupe *An. funestus* ou du complexe An gambiae ont été identifiées morphologiquement; et la PCR a permis de confirmer les espèces. L'ELISA a été utilisée pour détecter les anophèles infectés par *Plasmodium* spp et pour identifier l'origine des repas sanguins chez les anophèles gorgés. Sur les 147 anophèles collectés 45 (30,2%) sont des *An. funestus funestus* et 101 (69,8%) sont des *An. arabiensis.* Deux des 24 *An. funestus* gorgés ont pris leurs repas sanguins sur des humains (Human Blood Index = 8,3%; IC95%: 1,5 - 28,5%). Le taux d'infection à Plasmodium spp était de 2,2% [IC à 95%: 0,4 - 11,6%] chez *An. funestus.* Nos résultats ont montré qu*‘An. funestus*a persisté jusqu’à huit mois après la CAID utilisant Actellic 300 CS. En plus, même si nous n'avions pas trouvé d*‘An. arabiensis* infectés par à *Plasmodium* spp durant cette étude, le rôle de cette espèce dans la transmission est une certitude dans cette région. Des cas de paludisme ont été détectés parmi les villageois symptomatiques lors de nos missions sur site. En conclusion, *An. funestus funestus* persiste six mois après aspersion intra-domiciliaire de Pyrimiphos-methyl à Ankililoaka. Le contact Homme-vecteur est maintenu et appuyé par An. arabiensis, cela contribue à la persistance d'une transmission résiduelle du paludisme. L'impact de la création des barrages pour développer les activités agricoles irriguées sur la transmission du paludisme dans notre zone d’étude sera discuté dans notre présentation.

# Research article: Test de diagnostic rapide du paludisme (mRDT) au niveau communautaire à Madagascar: faut-il multiplier les réalisateurs?

**Published:** 2021-11-30

**Authors:** S. Razanatsiorimalala, E.N. Raholimalala, H. Tseheno, M. Ravaoarimanga, D. Randriamiarinjatovo, M. Randrianarivelojosia

**Affiliations:** Institut Pasteur de Madagascar, Antananarivo, Madagascar

**Keywords:** Paludisme, mRDT, Agent communautaire, Mananjary, Madagascar, Océan Indien

Dans la lutte contre le paludisme, diagnostiquer avant de traiter est l'idéal. Différentes méthodes sont utilisées à Madagascar pour détecter les infections plasmodiales dont le test de diagnostic rapide du paludisme (mRDT). Afin de sauver des vies et d’éviter le paludisme grave, la prise en charge du paludisme au niveau communautaire est à promouvoir. Dans le cadre du projet PECADOM à Mananjary, un dépistage actif du paludisme par mRDT détectant panLDH et pfHRP2 a été réalisé dans 10 communes de décembre 2016 à février 2017. Notre présentation porte sur les infections plasmodiales sur les individus de tout âge. Sur les 47.754 sujets examinés, 3172 (6.6%; IC 95%: [6.4-6.8] mRDT ont été positifs. La prévalence de l'infection plasmodiale variait de 1,9% à 19.1% selon les communes. Deux communes étaient très infectées: Namorona et Antsenavolo avec des prévalences d'infection plasmodiale à (19.1%; IC 95%: [17.8-20.5]) et (17.8%; IC 95% :[16.5-19.1]) respectivement. Les enquêteurs qui ont réalisé les mRDT ont été formés à l'Institut Pasteur de Madagascar; et leur performance dans la réalisation des mRDT était excellente. Le mRDT répond aux réalités dans les localités éloignées, sans eau courante ni électricité à Madagascar. Par contre, pour diverses raisons, les agents communautaires des sites d’étude qui devaient travailler avec l’équipe du projet n’étaient pas forcement disponibles. Au-delà de la réalisation d'un projet, afin de réaliser tout le temps les mRDT au niveau des communautés, ne faut-il pas prendre en considération l'implication d'autres réalisateurs qui ont un niveau d’éducation acceptable comme les instituteurs ou les religieux. On tient à rappeler que lors de la riposte à l’épidémie meurtrière de paludisme des années 1980 à Madagascar, des enseignants, des épiciers, des volontaires de la Croix-Rouge, etc. ont été sollicités. La place des mRDT au pied du lit des malades dans les hôpitaux sera discutée dans notre présentation.

# Research article: Campagne de distribution de masse des médicaments (mébendazole) contre les géohelminthiases chez les enfants âgés de 5 à 14 ans au Gabon en février 2020

**Published:** 2021-11-30

**Authors:** Julienne Atsame

**Affiliations:** Programme de lutte contre les maladies parasitaires, ministère de la Santé, Libreville, Gabon

**Keywords:** Helminthiases, Chimioprévention, Mébendazole, Traitement de masse, Gabon, Afrique subsaharienne

Les vers intestinaux (géo-helminthiases) appartiennent au groupe des maladies tropicales négligées (MTN). L'OMS, à travers sa feuille de route relative à la lutte contre les MTN, s'est engagée à éliminer les géo-helminthiases comme problème de santé dans les pays endémiques d'ici 2030, grâce à l'organisation de la distribution de masse de médicaments (DMM).

Le Gabon est endémique aux géo-helminthiases dans les 52 départements sanitaires (DS) que compte le pays. Le Gabon s'est engagé à réaliser la DMM aux populations cibles, qui sont les enfants âgés de 5 à 14 ans des zones endémiques. C'est dans ce processus que s'inscrit la présente campagne réalisée en février 2020.

Au total 277.093 enfants ciblés, la DMM dans les écoles, fut celle privilégiée dans les départements sanitaires, à cause du taux national de scolarisation élevé. Avec une couverture thérapeutique nationale (CT) obtenue de 63%, 68% des DS endémiques ont atteint ou dépassé la performance escomptée qui est ≥ 75%. La campagne nationale de distribution de masse du mébendazole a été menée, grâce à la mobilisation respective des équipes à tous les niveaux, à l'implication des autorités politico-administratives et à l'appui surtout technique et financier de l'OMS malgré la faible appropriation des activités par les acteurs scolaires.

Ainsi, beaucoup d'efforts reste à conjuguer pour atteindre l'objectif global que nous assigne la feuille de route de l'OMS. Cela ne sera possible que lorsque le gouvernement (en collaboration avec ses partenaires techniques et financiers notamment l'OMS) réaffirmera une fois de plus son leadership à la mise en œuvre d'une feuille de route consensuelle permettant de lever tous les goulots d’étranglement liés aux DMM.

# Research article: Fréquence et facteurs associés à la prescription du métronidazole dans les formations sanitaires d'Abomey-Calavi (Bénin): étude rétrospective

**Published:** 2021-11-30

**Authors:** Z.H. Tankpinou, J.-F. Faucher, D. Affolabi

**Affiliations:** Université de Limoges, 33 rue François Mitterrand, BP23204, 87000 Limoges, France

**Keywords:** Métronidazole, Facteurs associés, Prescriptions, Bénin, Afrique subsaharienne

Les habitudes de consommation des antibiotiques, varient selon les continents. Malgré l'absence de surveillance de la consommation des antibiotiques en Afrique, plusieurs études ont montré que le métronidazole était un antibiotique qui y était très consommé à l'instar de l'amoxicilline ou de la ciprofloxacine. Notre étude visait à évaluer la fréquence de prescription de ce médicament en ambulatoire et les déterminants liés à son utilisation dans les formations sanitaires d'Abomey-Calavi.

Il s'agissait d'une étude rétrospective et transversale. Nous avons inclus dans notre étude 11 centres de santé publics et privés dans lesquels deux cents consultations ont été sélectionnées entre le 1^er^ janvier 2020 et le 31 décembre 2020. Les données socio-démographiques, cliniques, paracliniques, thérapeutiques ont été collectées. Notre critère de jugement principal était la prescription de métronidazole.

Au total, 2200 consultations, ont été inclus dans l’étude. La population féminine était prédominante. L’âge médian était de 19 ans avec un intervalle interquartile de 6 à 32 ans. La fréquence de prescriptions du métronidazole était de 18% dans l'ensemble de l’étude et était plus élevée dans le secteur privé (20%). Les facteurs associés étaient nombreux. Les symptômes digestifs avec diarrhée (OR=43,02; IC 95% :24,10-78,74; p<0,001), les diagnostics d'infections digestives (OR=3,5; IC 95%: 2,06-5,94; p<0,001), d'ulcères gastriques (OR=9,81; IC 95%: 3,21-31,59; p<0,001) et d'infections génito-urinaires étaient les facteurs qui augmentaient considérablement le risque de prescription du métronidazole.

Le métronidazole est un médicament très consommé dont la prescription peut être fortement influencée par la diarrhée qui est généralement d'origine virale. Il semble être beaucoup plus prescrit dans le secteur privé.

# Research article: Facteurs associés à la tuberculose multirésistante en Guinée-Bissau du 1^er^ janvier 2018 au 31 décembre 2019

**Published:** 2021-11-30

**Authors:** P.K. Yanogo, A.L. Papique, N. Meda

**Affiliations:** Burkina Field Epidemiology and Laboratory Training Program (BFELTP), Ouagadougou, Burkina Faso

**Keywords:** Tuberculose multirésistante, Facteurs associés, Guinée Bissau, Afrique subsaharienne

La résistance aux médicaments antituberculeux est un problème majeur de santé publique qui compromet les succès remportés en matière de lutte contre la tuberculose. La tuberculose multirésistante apparaît le plus souvent quand les antibiotiques sont mal utilisés chez des patients dont la tuberculose est sensible.

L'objectif de cette étude est de déterminer les facteurs associés à la tuberculose multirésistante en Guinée-Bissau. Il s'agit d'une étude cas-témoins non appariés menée du 1^er^ janvier 2018 au 31 décembre 2019. Les variables utilisées ont été regroupées en caractéristiques sociodémographiques, profil clinique et biologique. Une analyse par régression logistique a estimé les OR d'association avec la tuberculose multirésistante ainsi que leur intervalle de confiance à 95%. Le seuil de significativité retenue était de 0,05.

L’échec du traitement antérieur [ORa = 14,93; IC95% (5,22-42,64); p = 0,0001] et l'interruption de traitement momentanée [ORa=5,26: IC95% (1,88-14,75); p=0,0016] étaient associés au développement de la tuberculose multirésistante. Le respect des heures de prise de médicaments [ORa =0,18; IC95% (0,06-0,57) p= 0,0033] protégeait contre la tuberculose multirésistante.

Malgré les progrès importants dans la lutte contre la tuberculose, un ensemble d'actions articulées est nécessaire pour faire barrière au développement des résistances aux antituberculeux. Le suivi rigoureux ainsi que des mesures de protection et de soutien sociaux aux patients.

# Research article: Vaccination contre la tuberculose, la poliomyélite et l'hépatite B à la naissance: couverture vaccinale et facteurs associés au délai de vaccination dans le district sanitaire de Podor (Sénégal)

**Published:** 2021-11-30

**Authors:** O. Bassoum, M. Anne, M.F. Ba, M. Bocoum, A. Dieye, C. Sokhna, A. Tal-Dia

**Affiliations:** Institut de santé et développement, Université Cheikh Anta Diop de Dakar (UCAD), Dakar 10700, Sénégal

**Keywords:** Vaccination, Couverture vaccinale, Tuberculose, Poliomyélite, Hépatite B, Podor, Sénégal, Afrique subsaharienne

Au Sénégal, les études portant spécifiquement sur la couverture vaccinale par le vaccin bacille de Calmette et Guérin (BCG), la dose de naissance du vaccin antipoliomyélitique oral (VPO zéro) et la dose de naissance du vaccin anti-hépatite B (DN du HepB) sont insuffisantes. L'objectif était de mettre en évidence des couvertures vaccinales et les facteurs associés à la vaccination en temps voulu dans le district sanitaire de Podor.

Une étude transversale analytique était réalisée du 19 au 22 juin 2020. La population d’étude était constituée des enfants âgés de 12 à 23 mois parmi lesquelles 832 ont été inclus. Un sondage stratifié en grappes à deux degrés était réalisé. Les sources des données étaient la fiche de santé conservée à domicile, le registre de vaccination et la déclaration de la mère ou gardienne. La vaccination en temps voulu désigne toute vaccination ayant eu lieu dans les 24 heures suivant la naissance. Des analyses descriptives, le test du chi-deux et la régression logistique étaient réalisés.

Les couvertures vaccinales brutes par le BCG, le VPO zéro et la DN du HepB étaient respectivement égales à 95,2%, 88,3% et 88,1%. Les couvertures vaccinales dans les 24 heures suivant la naissance sont respectivement estimées à 13,9% %, 30% et 42,1%. Les facteurs associés à l'administration de la DN du HepB en temps voulu sont l'accouchement en structure sanitaire (ORa=1,55; IC95%=1,02-2,40), l'accès à la télévision (ORa=1,63; IC95%=1,16-2,29), la pesée (ORa=3,92; IC95%=1,97-8,53) et l'hospitalisation du nouveau-né immédiatement après la naissance (ORa=0,42; IC95%=0,28-0,62).

L'administration des doses de naissance en temps voulu est un défi dans le district sanitaire de Podor. Les solutions seraient l'amélioration de l'accès géographique aux structures sanitaires, l'implication des agents de santé communautaire, la sensibilisation et l'intégration des services de santé.

# Research article: Baisse de la mortalité précoce chez les patients atteints de Covid-19 suivis à Brazzaville: impact de la CPAP (*Continuous Positive Airway Pressure*)

**Published:** 2021-11-30

**Authors:** M. Amona, R. Oboba Ondze, A.G. Aloumba, T. Adoua Doukaga, M. Ekat, B.R. Ossebi Ibara, J. Dzunu-Kinuani, M. Sapoulou, A. Elira Dokeikias, H.G. Monabeka, P. Ibata

**Affiliations:** Ministère de la Santé du Congo, Commission nationale de prise en charge des cas de Covid-19, Brazzaville, Congo

**Keywords:** Covid-19, Baisse de la mortalité, CPAP (Continuous Positive Airway Pressure), Pression positive continue, Brazzaville, Congo, Afrique subsaharienne

Les thérapies spécifiques pour obtenir une rémission virologique complète soutenue dans la Covid-19 ne sont pas encore disponibles. Cependant certaines expériences montrent leur efficacité. C'est dans cette mesure que nous avons réalisé cette étude observationnelle pour montrer l'impact de l'introduction de l'oxygénothérapie en pression positive continue (CPAP) sur la réduction de la mortalité chez les patients atteints de Covid-19 symptomatiques suivis au centre de référence de Brazzaville.

Il s'est agi d'une étude observationnelle à visée descriptive menée au centre de référence de prise en charge des cas de Covid-19 de Brazzaville entre mars et juin 2020, soit une période de trois mois. Ont été inclus, tous les patients admis avec une saturation partielle en oxygène inférieure à 90% quel que soit le tableau clinique.

Un total de 226 cas confirmés de Covid-19 a été enregistré soit une moyenne d'admission mensuelle de 75,3 cas. La majorité des cas était âgée de plus de 31 ans dans 74,30% des cas. Le sexe masculin était majoritaire dans 58,40% des cas.

Les comorbidités fréquemment retrouvées étaient représentées par l'hypertension artérielle dans 24,33%, l'obésité dans 10,61%, le diabète dans 9,73%, la tuberculose pulmonaire active dans 2,65%, la grossesse dans 1,77% des cas, l'infection par le VIH dans 2,65% des cas, la BPCO dans 3,54% et l'asthme dans 3,98%.

La classification selon le stade de gravité retrouve 33,19% des cas au stade modéré, 55,31% des cas au stade sévère et 11,50% des cas au stade critique. La CPAP a été pratiquée chez 69,91% des patients et 30,09% des patients n'ont pas bénéficié de la CPAP pour les raisons logistiques liées à la rupture de l'oxygène et aux problèmes d'adaptation du début de l’épidémie.

Le délai de récupération était court de 3 jours chez les cas sans comorbidité ayant bénéficié d'une CPAP dans 18,14%; de 5 jours chez ceux avec comorbidité ayant bénéficié d'une CPAP dans 51,80%. Il était moyen de 5 à 10 jours chez les patients sans comorbidités n'ayant pas bénéficié de la CPAP dans 92,48%. Le délai était long de plus de 10 jours chez les patients avec comorbidités n'ayant pas bénéficié de la CPAP dans 7,52%. Cette série a enregistré 8 décès, soit un taux de létalité hospitalière de 2,65%.

L'oxygénothérapie par CPAP fait la preuve d'une efficacité certaine dans la gestion des cas de Covid-19 avec un impact perceptible chez les patients. Mais une étude randomisée à grande échelle est nécessaire pour prouver cette efficacité.

# Research article: Expérience de prise en charge hospitalière de la Covid-19 au Centre hospitalier régional Georges Rawiri, Lambaréné (Gabon)

**Published:** 2021-11-30

**Authors:** M. Diallo, D. Eyamame, E.G. Rossatanga

**Affiliations:** Centre hospitalier régional Georges Rawiri, Lambaréné, Gabon

**Keywords:** Covid-19, Prise en charge, Hôpital, Traitement, Lambaréné, Gabon, Afrique subsaharienne

Dès l'apparition des premiers cas de Covid-19 à Libreville au Gabon en mars 2020, la province du Moyen-Ogooué en raison de sa situation géographique au centre du Gabon, s'est sentie menacée. C'est ainsi que sous la présidence du gouverneur de province, a été créé un Comité provincial du plan de veille et de riposte contre l’épidémie à coronavirus.

Le cadre de l’étude est la région sanitaire centre (Province du Moyen Ogooué). Population desservie de la région sanitaire centre: 69 287 habitants. Le service de maladies tropicales situé au Centre hospitalier régional Georges Rawiri de Lambaréné (CHRGRL), structure de référence de la région sanitaire, a été choisi pour abriter le site de prise en charge hospitalière Covid-19, avec une capacité hospitalière de 19 lits dédiés dont deux réservés aux soins intensifs.

Il y avait 19 lits, dont deux réservés pour les soins intensifs. La prise en charge des patients atteints de Covid-19 hospitalisés a été assurée par une équipe composée d'un médecin, de 10 Infirmiers, de 5 psychologues, d'un hygiéniste et de deux techniciennes de surface.

Présentation de la prise en charge des cas de Covid-19 sur une période de 12 mois allant de mars 2020 à février 2021. Critères diagnostiques: RT-PCR positif

Sur un total de 495 cas positifs, nous avons hospitalisé 96 patients.

Les critères d'hospitalisation étaient: (i) comorbidités; (ii) signes modérés et sévères (détresse respiratoire; (iii) altération de l’état général; (iv) syndrome infectieux. La tranche d’âge la plus atteinte était celle de 40 à 49 ans (34% des cas). Le sex-ratio était de 78% d'hommes contre 22% de femmes.

Parmi les comorbidités on relevait: HTA (16), diabète (5), HTA + Diabète (4), drépanocytoses (3), maladies respiratoires chroniques (2), VIH (2), autres (2)

Les protocoles thérapeutiques suivants ont été mis en place:

Type 1: azithromycine + chloroquine + vitaminothérapie: 27 patients
Type 2: azithromycine + amoxicilline-clavulanique + vitaminothérapie: 23 patients
Type 3: azithromycine + cefixime + vitaminothérapie, oxygénothérapie: 8 patients
  - Traitements symptomatiques, vitaminothérapie: 38 patients ayant des signes modérés

L’évolution des cas a été la suivante:

- Guérison: 88 patients, deux tests PCR négatifs successifs avant la sortie de l'hôpital
- Sortie avant guérison pour poursuite du confinement à domicile: 7 patients pour des raisons familiales
- Décès enregistrés: un décès (causes du décès: VIH/choc septique/insuffisance respiratoire)

Les difficultés rencontrées ont été: (i) déni de la maladie par les patients et leurs familles; (ii) la stigmatisation; (iii) le long séjour à l'hôpital (durée moyenne d'hospitalisation 21 jours); (iv) absence de tomodensitomètre; (v) manque de réanimateur; (vi) ruptures de stick de matériel de protection (EPI) et d'intrants; (vii) burnout du personnel soignant.

# Research article: Vaccination rVSV-Zebov des personnels des organisations non gouvernementales: expérience française

**Published:** 2021-11-30

**Authors:** C. Ficko, V. Lamand, P.-L. Conan, M. Gominet, M. Cabon, L. De Moissy, F. Manificat, N. Massy, D. Andriamanantena

**Affiliations:** Hôpital d'Instruction des Armées Bégin, 69 Av. de Paris, 94160 Saint-Mandé, France

**Keywords:** Ebola, Vaccination, rVSV-Eebov, Pharmacovigilance, ONG, France

En France, la vaccination Ebola est recommandée en prévention pour les professionnels susceptibles d’être exposés, et en post-exposition dans les suites d'un contact auprès d'un cas confirmé. Du fait de l'absence d'autorisation de mise sur le marché (AMM) jusqu'en mars 2020, elle a été réalisée dans le strict respect de son protocole temporaire d'utilisation.

Notre hôpital a été sollicité afin de mettre en œuvre cette vaccination au profit des personnels des ONG (organisations non gouvernementales) se rendant en République démocratique du Congo dans le cadre de la riposte à l’épidémie.

L'objectif de cette étude est de décrire le circuit d'approvisionnement, de délivrance, d'administration et de traçabilité de la vaccination rVSV-Zebov, la population vaccinée et les effets indésirables sur la période du 20/12/2018 au 31/12/2020.

Il s'agit d’étude rétrospective observation nelle descriptive monocentrique.

En amont de chaque séance, les ONG sollicitaient la Direction générale de la santé qui validait et priorisait les demandes. Les doses vaccinales étaient livrées avant chaque séance par l’établissement de ravitaillement de Santé publique France, afin de tenir compte du délai de conservation après décongélation (14 jours). Les personnels, volontaires pour être vaccinés, signaient un consentement après information orale et écrite. Une pharmacovigilance renforcée était mise en place. Deux cent quarante-trois personnels ont été vaccinés, 218 travaillant au profit d'une ONG (90%). Le sex-ratio était de 0,9 (117 hommes pour 126 femmes), l’âge médian de 36,8 ans (ET 10.7, extrêmes 21.2-71.2). Les personnels venaient de France, d'Europe et d'autres continents dans respectivement 72.2, 9.5 et 18.3% des cas. Cinquante-trois pour cent n'ont présenté aucun effet indésirable immédiat. La plupart des effets indésirables étaient limités au site d'injection (douleur et fasciculation chez respectivement 31.3% et 19.3% des cas). Sept personnels présentaient un malaise, dont un avec perte de connaissance, d’évolution dans tous les cas favorable. Un effet indésirable retardé était rapporté chez 70 vaccinés (28.8%), principalement des syndromes pseudo-grippaux (65 cas) survenant dans les 24 heures et disparaissant dans les 72 heures. Dix sujets rapportaient une arthralgie survenue entre le 3^e^ et le 11^e^ jour post-vaccinal, dont 5 cas évocateurs d'arthrite. L’évolution était favorable chez 9 sujets, inconnue pour le dernier.

La vaccination Ebola a été rendue possible en France du fait de la mobilisation de nombreux acteurs et des interactions entre plusieurs structures sanitaires.

L'obtention d'une AMM en 2020, du fait de données d'efficacité et de tolérance favorables, devrait permettre de faciliter son administration.

# Research article: Impact de la Covid-19 sur la vaccination de routine en milieu hospitalier au Sénégal

**Published:** 2021-11-30

**Authors:** M. Gueye, A. Sow, D. Boiro

**Affiliations:** Faculté de médecine de l'Université Cheikh Anta Diop de Dakar (UCAD), Dakar 10700, Sénégal

**Keywords:** Covid-19, Vaccination, Hôpital, Impact, Dakar, Sénégal, Afrique subsaharienne

La pandémie Covid-19 a poussé le monde à mettre en œuvre des méthodes de prévention drastiques basées sur la limitation des mouvements des populations ayant un impact sur les politiques de santé publique comme la vaccination. L'objectif de ce travail était d’évaluer l'impact de ces mesures de prévention sur la vaccination de routine en milieu hospitalier depuis l'avènement de cette pandémie au Sénégal.

Il s'agit d'une étude transversale rétrospective réalisée en août 2020 dans l'unité de vaccination du centre hospitalier Abass NDAO. Nous avons comparé les données de l'unité de vaccination durant la période allant de mars à août des trois dernières années (2018, 2019 et 2020). Le paramètre étudié était le nombre de doses de vaccins administrées pour les différentes périodes selon le programme élargi de vaccination.

Pour les vaccins administrés à la sixième semaine en avril, le nombre de doses était de 36 en 2018, 29 en 2019 et 15 en 2020 soit une baisse de 50%. Au mois de juillet le nombre de doses administrées était de 40 en 2018, 35 en 2019 et 15 en 2020 soit une réduction de 42% par rapport à 2019.

Les mesures de lutte contre cette pandémie ne devraient pas faire oublier la vaccination de routine surtout dans nos pays à ressources limitées. Il est indispensable de poursuivre la vaccination pour les enfants et d'identifier les enfants qui ont raté des doses vaccinales pour un rattrapage.

# Research article: Investigation des cas de Covid-19 dans la région de Zinder (Niger)

**Published:** 2021-11-30

**Authors:** M. Mahamadou Yacouba, A. Ibrahim, S. Tiendrebeogo, P. Yanogo, J. Kaboré, A. Yacouba, N. Meda

**Affiliations:** Burkina Field Epidemiology and Laboratory Training Program (BFELTP), Ouagadougou, Burkina Faso

**Keywords:** Covid-19, Investigation, Décentralisation, Zinder, Niger, Afrique subsaharienne

Le 30 janvier 2020, face à la propagation rapide de l’épidémie de la Covid-19 dans le monde, l'OMS décrète l'urgence de santé publique de portée internationale. L'Afrique a commencé à être touchée après l'Asie et l'Europe. Le premier cas signalé de Covid-19 en Afrique a eu lieu le 14 février 2020 en Égypte. Malgré les mesures de contrôle aux frontières, le Niger n'est pas parvenu à s'isoler du reste du monde et le 19 mars 2020 le Niger a enregistré son premier cas de Covid-19. Après Niamey, ce fut la région de Zinder au Niger qui a enregistré son premier cas de Covid-19 le 02/04/2020.

Cette étude transversale analytique a été effectuée du 3 avril 2020 au 2 septembre 2020. La population d’étude est constituée de tous les cas suspects et contacts de Covid-19 pendant la période d’étude au niveau de 11 districts sanitaires de la région de Zinder. Les données ont été collectées à partir des fiches d'investigation et prélèvement des cas. L'analyse a été faite à l'aide du logiciel Epi-info version 7.2.3.1 et du tableur Excel 2013. Des proportions, des fréquences, des Odds Ratio avec leurs intervalles de confiance à 95% et la P valeur ont été calculés. Les résultats ont été présentés sous forme de tableaux et de figures.

Au total, 426 cas suspects de Covid-19 ont été prélevés et testés par RT-PCR au niveau de 11 districts sanitaires de la région de Zinder. Parmi eux, 135 (31,69%) étaient positifs et 24 (5,63%) patients étaient décédés. L’âge moyen des cas était de 44,89 ± 19,25 ans, le sex-ratio H/F est de 1,63. La toux et la fièvre dominaient le tableau clinique avec respectivement 79,48% et 73,58%. Le résultat de la RT-PCR variait significativement avec l’âge (P=0,0004 OR=3,58 IC95% [1,80-7,10]); le sexe (P=0,04 OR=1,55 IC95% [1,01-2,39]) et la notion de contact avec un cas confirmé de covid-19 (P=0,02 OR=2,49 IC95% [1,10-5,61]).

Cette étude fournissait les caractéristiques socio-démographiques des premiers patients de Covid-19 dans la région de Zinder au Niger. La létalité de cette maladie était élevée (17,77%) à Zinder. Les résultats de cette étude ont démontré l'intérêt de la décentralisation de la prise en charge des patients atteints de Covid-19, au début réalisée uniquement au niveau de Niamey, la capitale du Niger.

# Research article: Le pagne: un outil de sensibilisation original pour la prévention du VIH utilisable contre la Covid-19

**Published:** 2021-11-30

**Authors:** Jean-Loup Rey, François Deniaud

**Affiliations:** GISPE (Groupe d'intervention en santé publique et épidémiologie), 82 boulevard Tellène, 13008 Marseille, France

**Keywords:** Covid-19, Sensibilisation, CCC, pagne, Afrique de l'Ouest

En Afrique, surtout de l'Ouest, le pagne, pièce de tissu en coton imprimé, est un objet de consommation très apprécié des femmes. À la sortie de l'usine il a une dénomination commerciale, mais quand il est utilisé, les femmes lui donnent un surnom. Ce surnom est, dans environ la moitié des cas, du domaine des relations femmes-hommes, le pagne devient alors un mode de communication spontané, plus ou moins codé: « Œil de ma rivale », « les jaloux vont maigrir », « Ton pied, mon pied », « tu sors, je sors », etc.

Dans les années 90 nous avions mis en route des projets utilisant le pagne comme moyen de sensibilisation pour la prévention du sida, un pagne créé pour cela a beaucoup été utilisé par les jeunes, un autre a été surnommé « sida » par les utilisatrices.

Dans le cadre de la Covid-19, nous proposons de reprendre cette démarche pour promouvoir les gestes de prévention et/ou le port du masque.

# Research article: Connaissances, attitudes et pratiques sur la Covid-19 des professionnels de santé des centres d'accueil et de prélèvement Covid-19 en Côte d'Ivoire (2020)

**Published:** 2021-11-30

**Authors:** M.-N. Ano Ama Kounangui, L.N. Aka, F.K. Ekou, E.M. Ahoussou, C. Akani Bangaman, P.S. Sablé, E. Kouassi, D. Ekra Kouadio, S. Dagnan N'Cho

**Affiliations:** Institut national d'hygiène publique (INHP), Bp V14 Abidjan, Treichville, Rue Pasteur

**Keywords:** Connaissance, Attitude, Pratique, Covid-19, Professionnels de santé, Abidjan, Côte d'Ivoire, Afrique subsaharienne

La Covid-19 est une urgence de santé publique de portée internationale à l'origine d'une pandémie depuis le 11 mars 2020. Face à ce problème, en Côte d'Ivoire des mesures administratives couplées à des mesures sanitaires ont été promulguées mettant en première ligne les professionnels de santé.

La présente étude nous a permis d’étudier les connaissances, attitudes et pratiques des professionnels de santé et d'identifier les facteurs associés.

Il s'agit d’étude transversale à visée analytique. Cette étude a eu pour cadre les centres d'accueil et de prélèvement Covid-19 des deux régions sanitaires d'Abidjan. Le nombre de personnes à enquêter dans les centres d'accueil et de prélèvement a été déterminé grâce à l'utilisation de la formule de Stuart Pocock. Les données ont été saisies à l'aide du logiciel EPI data et analysées à l'aide du logiciel R.

L’âge moyen des professionnels de santé était de 32,4 ± 8,9, dominé par les hommes sex-ratio 1,96. La majorité des professionnels étaient des médecins. L'expérience professionnelle médiane était de 2 ans.

Au niveau des connaissances, 47,89% des médecins et 32,63% des paramédicaux ont donné de bonnes réponses. Pour ce qui était des attitudes, la majorité des professionnels avaient des attitudes correctes. Et 62,8% ont exprimé des sentiments de peur et d'inquiétudes face à la Covid-19.

Au niveau des pratiques seulement 26,88% des médecins et 20,22% des paramédicaux avaient de bonnes pratiques à l’égard due la Covid-19. Une association significative entre le fait d'avoir un enfant en charge et les pratiques a été trouvé avec un p=0,021.

Notre étude a montré que des efforts restaient à faire pour améliorer la sensibilisation des professionnels de santé en vue d'une meilleure connaissance, attitude et pratique sur la Covid-19.

# Research article: Étude de la persistance des anticorps anti-SARS-CoV2 chez le personnel de santé du CHU d'Oran atteint de Covid-19

**Published:** 2021-11-30

**Authors:** N. Guetarni, N. Heroual, S. Zouagui, F. Ammour, N. Hebali, F.Z. Besbes, Bendjoudi Kadiri, H.M. Adda, N. Berramdane, K. Ziat, A. Feghoul, Z. Akabi, H. Messid, K. Terki

**Affiliations:** Centre hospitalier universitaire d'Oran, Boulevard Doctor Benzerdjeb, Oran, Algérie

**Keywords:** Covid-19, Anticorps anti-SARS-CoV2, Séroprévalence, Immunité, Oran, Algérie, Afrique du Nord

Durant cette épidémie, le centre hospitalier universitaire d'Oran est désigné pour la prise en charge des cas de Covid-19 à Oran. L'objectif de l’étude est d'estimer, la proportion des patients présentant des anticorps IgM et IgG à différentes périodes après l'infection, d’étudier la relation de la persistance des anticorps avec certains facteurs tels que les symptômes, les antécédents médicaux des patients.

Il s'agit d'une étude descriptive de type transversale qui s'est déroulée au mois de mai 2021. Les patients enquêtés sont tirés au sort à partir d'une base de données (liste de personnels ayant contracté la Covid-19). Sont inclus dans l’étude, les cas probables et les cas confirmés. Les patients dont la Covid-19 remonte à moins de 3 mois ne sont pas inclus. Pour chaque patient, un questionnaire préétabli est renseigné et une recherche des anticorps est faite avec le test rapide d'orientation diagnostique (TROD).

L'analyse préliminaire montre un âge moyen de 40,4±10,3 ans chez la population d’étude. Le sexe féminin est prédominant avec 76,3%. Les antécédents médicaux sont retrouvés chez 40% des patients. L'hypertension artérielle vient en 1e position (17%), suivie de l'asthme (12%), l'obésité (8%). Les patients ont présenté une symptomatologie de covid-19 dans 98,7%. Soixante pour cent des patients avaient des IgG au moment de l’étude et seulement 5,3% avaient des IgM. Notre étude a montré la persistance des IgG au-delà de 5 mois après la contraction de l'infection due au Covid-19 chez 60,4% des patients.

# Research article: Facteurs associés au non-port de masques durant la pandémie de Covid-19 dans le district sanitaire de Ratoma (Guinée, avril 2020)

**Published:** 2021-11-30

**Authors:** P.K. Yanogo, T.J. Millimouno, N. Meda

**Affiliations:** Burkina Field Epidemiology and Laboratory Training Program (BFELTP), Ouagadougou, Burkina Faso

**Keywords:** Covid-19, Non-port de masques, Facteurs associés, Comportements, Ratoma, Guinée, Afrique subsaharienne

En Guinée, le taux d'attaque de la Covid-19 est passé de 0,13 à 11,25 cas/100 000 habitants de mars en avril 2020, malgré les efforts soutenus par le président du pays, rendant obligatoire le port de masque par la population toute entière comme le recommande l'OMS, la recommandation n’était pas suivie à Ratoma. L'objectif de notre étude était d'identifier les facteurs associés à ce non-port de masques afin de mieux prendre des actions ciblées pour contrôler la Covid-19.

Nous avons, dans une étude transversale analytique interviewé 1624 personnes. En analyse étiologique, nous avons, dans une régression logistique univariée puis multivariée recherché les variables indépendamment associées au non-port du masque; des OR ajustés avec IC95% ont été calculés. Les analyses ont été effectués par les logiciels Epi-info version 7.2.1 et Stata.

Des 1 624 personnes l’âge médian était de 37 ans. Plus de 50% d'entre eux n'avaient pas porté de masques et 44% ne connaissaient pas la nécessité de les utiliser. L’âge [20-39 ans (OR=0,61; IC95%=[0,45 – 0,82]; 40-59 ans (OR=0,37; IC95%=[0,25 – 0,54]; 60 ans et plus (OR=0,39; IC95%=[0,19 – 0,81]; p=0,003], l'intolérance du masque (OR=5,32; IC95%=[1,02 – 10,84] p=0,007) et le coût élevé du masque (OR=16,2; IC 95% = [10,14 – 20,17]; p<0.0001) ont été identifiés comme facteurs significativement associés au non-port de masques dans le district de Ratoma.

L'intolérance au masque, le prix élevé des masques étaient des facteurs modifiables indépendamment associés au non-port de masques. Nous recommandons de fournir des masques à prix abordables pour la population, d'accentuer la sensibilisation sur les pratiques de port de masques et de distribuer les masques.

# Research article: Contribution du Service de santé des armées au suivi des sujets contacts dans la riposte à la Covid-19 dans le Grand Abidjan (Côte d'Ivoire) de juin à décembre 2020

**Published:** 2021-11-30

**Authors:** F. Makonga, F. Kadjo, S. Kroman Savané, E. Kangah, B.S. Seri, D. Coulibaly, N. Yao, J. Bénié Bi Vroh

**Affiliations:** Direction de la santé et de l'action sociales des armées, Abidjan, Côte d'Ivoire

**Keywords:** Covid-19, Epidémiologie, Sujet contact, Armée, Multisectoriel, Grand Abidjan, Côte d'Ivoire, Afrique subsaharienne

Dans la gestion multisectorielle de la Covid-19, la Direction de santé et de l'action sociale des armées de Côte d'Ivoire (DSASA) du ministère de la Défense a collaboré avec le Centre des opérations d'urgences de santé publique (COU SP) en vue de limiter le risque de propagation de l’épidémie dans les unités, services et casernes des Forces armées. L'une des activités essentielles, en plus de la mise en place de l’équipe d'intervention rapide des armées, était le suivi des sujets contacts dans les zones signalant des cas ou ayant les paramètres à haut risque. Les districts sanitaires d'Abidjan qui cumulent 95% des cas confirmés de Covid-19 ont été prioritairement ciblés pour cette activité. Quels ont été les résultats des activités menées par les équipes du Service de santé des armées?

Nous avons mené une analyse descriptive des données de suivi de contacts de juin à décembre 2020 correspondant à la période inter-épidémique entre la première et la seconde vague épidémique, marquée par une réduction du nombre de cas.

La DSASA a été impliquée avec 20 sous-officiers du Service santé des armées et du personnel civil sous la supervision de 2 officiers médecins. Les équipes ont réalisé quotidiennement des visites à domicile pour la sensibilisation des ménages des sujets contacts, la prise de la température et la recherche des symptômes dans la période de 14 jours après la date du dernier contact avec un cas confirmé. Les données étaient transmises de façon hebdomadaire à l'INHP pour une synthèse de l'ensemble des districts.

De juin à décembre 2020, 103 cas de Covid-19 positifs ont été enregistrés au niveau du personnel de l'armée avec 721 sujets contacts soit une moyenne de 7 contacts par cas positifs.

L’âge des sujets contacts variait de 8 mois à 80 ans. La tranche d’âge la plus touchée concerne la population active des 15 à 45 ans soit 52% (375/721). La proportion de sujets contacts devenus positifs est de 13% (94/721). Cette activité communautaire a été marquée par des difficultés notamment le délai du rendu de résultat parfois trop long. Certains sujets contacts étaient injoignables et d'autres peu coopératifs.

Cette activité de suivi de contact a contribué à limiter la propagation de la maladie au sein des ménages et unités des Forces armées à travers une collaboration multisectorielle.
